# LINE-1 and *Alu* methylation signatures in autism spectrum disorder and their associations with the expression of autism-related genes

**DOI:** 10.1038/s41598-022-18232-6

**Published:** 2022-08-17

**Authors:** Thanit Saeliw, Tiravut Permpoon, Nutta Iadsee, Tewin Tencomnao, Valerie W. Hu, Tewarit Sarachana, Daniel Green, Chanachai Sae-Lee

**Affiliations:** 1 The Ph.D. Program in Clinical Biochemistry and Molecular Medicine, Department of Clinical Chemistry, Faculty of Allied Health Sciences, Chulalongkorn University, Bangkok, Thailand; 2 Research Division, SiMR, Faculty of Medicine Siriraj Hospital, Mahidol University, Bangkok, Thailand; 3 Natural Products for Neuroprotection and Anti-Ageing Research Unit, Chulalongkorn University, Bangkok, Thailand; 4 Department of Clinical Chemistry, Faculty of Allied Health Sciences, Chulalongkorn University, Bangkok, Thailand; 5 Department of Biochemistry and Molecular Medicine, School of Medicine and Health Sciences, The George Washington University, Washington, DC USA; 6 SYstems Neuroscience of Autism and PSychiatric Disorders (SYNAPS) Research Unit, Department of Clinical Chemistry, Faculty of Allied Health Sciences, Chulalongkorn University, Bangkok, Thailand; 7 Institute of Life Course and Medical Sciences, Faculty of Health and Life Sciences, University of Liverpool, Liverpool, UK

**Keywords:** Epigenetics, Autism spectrum disorders

## Abstract

Long interspersed nucleotide element-1 (LINE-1) and *Alu* elements are retrotransposons whose abilities cause abnormal gene expression and genomic instability. Several studies have focused on DNA methylation profiling of gene regions, but the locus-specific methylation of LINE-1 and *Alu* elements has not been identified in autism spectrum disorder (ASD). Here we interrogated locus- and family-specific methylation profiles of LINE-1 and *Alu* elements in ASD whole blood using publicly-available Illumina Infinium 450 K methylation datasets from heterogeneous ASD and ASD variants (*Chromodomain Helicase DNA-binding 8* (*CHD8*) and 16p11.2del). Total DNA methylation of repetitive elements were notably hypomethylated exclusively in ASD with *CHD8* variants. Methylation alteration in a family-specific manner including L1P, L1H, HAL, *AluJ*, and *AluS* families were observed in the heterogeneous ASD and ASD with *CHD8* variants. Moreover, LINE-1 and *Alu* methylation within target genes is inversely related to the expression level in each ASD variant. The DNA methylation signatures of the LINE-1 and *Alu* elements in ASD whole blood, as well as their associations with the expression of ASD-related genes, have been identified. If confirmed in future larger studies, these findings may contribute to the identification of epigenomic biomarkers of ASD.

## Introduction

Autism spectrum disorder (ASD) is a complex neurodevelopmental disorder characterized by two behavioral impairments: (i) deficits in social interactions and communication, and (ii) restricted interests and repetitive behaviors, according to the Diagnostic and Statistical Manual of Mental Disorders, Fifth Edition criteria^1^. According to the Centers for Disease Control and Prevention (CDC), the prevalence of ASD has risen dramatically over the last decade due to better screening methods. In 2018, ASD affected approximately one out of every 44 children in the United States^2^. ASD is currently understood as a multifactorial disorder, with the precise causes remaining unknown. Over the last two decades, research has attempted to elucidate the genetic origin of the disorder. However, genetic aberration is only found in 10–20% of ASD cases. In total, more than 60% of people with ASD are idiopathic^3^. Several studies have shown that ASD clinical phenotypic heterogeneity is influenced by a combination of genetic and environmental factors^4–6^. This evidence has highlighted non-genetic factors such as epigenetics (DNA methylation (DNAm)) and environmental interactions as key players in ASD progression. Additionally, some genetic factors that increase the risk of ASD, but only a few loci have a high impact on ASD^7^. The 16p11.2 deletion (16p11.2 del) and *Chromodomain helicase DNA-binding domain 8* (*CHD8*) variants are high genetic risk factors for ASD^8,9^. People with 16p11.2del are usually characterized by developmental delay, intellectual disability, or ASD^9^. *CHD8* is strongly associated with ASD and other neurodevelopmental disorders including schizophrenia and intellectual disability^10,11^.

Epigenetics is a family of heritable mechanisms that elicit control of gene expression without modification to DNA sequences^12^. Examples of epigenetic mechanisms are DNAm, RNA modification, and histone modifications^13^. DNAm, the most frequently studied epigenetic modification, involves the addition of methyl groups to DNA. Depending on its genomic location, the addition of a methyl groups to the 5th carbon atom of cytosine can have repressive or inductive effects on the gene expression. When DNAm is not properly maintained or established, methylation abnormalities can manifest in disease development. DNAm patterns are well known to show tissue-specific differentially methylated regions (DMRs). However, most loci present similar DNAm levels across a wide variety of tissue types. Interestingly, recent work has demonstrated the utility of blood as a surrogate for human brain tissue CpG methylation^14^. Therefore, blood-based epigenetic biomarkers have the potential to serve as non-invasive biomarkers for otherwise inaccessible tissues. For instance, some epigenetic markers in blood have been identified as biomarkers in early stages of Alzheimer’s disease^15^. Similar findings have also arisen in ASD, a recent meta-analysis of blood-based DNA demonstrated evidence of the associations between blood-based and brain samples in comparison between ASD and controls^16^.

Long interspersed nucleotide element-1 (LINE-1) and *Alu* elements are known as non-long terminal repeat retrotransposons that can replicate and insert themselves into different locations within the host genome. LINE-1 and *Alu* elements make up more than 25% of the human genome and have a copy number of over one million elements^17^. These repetitive elements (REs) can affect the expression of host or neighboring protein-coding genes through introducing alternative promoters or enhancers, novel splicing sites, and epigenetic alteration through DNAm^17^. Subfamilies of LINE-1 and *Alu* elements can be subcategorized by identifying variants in their sequences that have accumulated in the evolutionary heritage^18,19^. LINE-1 has been classified into three main subfamilies during early primate evolution including L1M (mammalian-specific, oldest), L1P (primate-specific, intermediate), and L1H (human-specific, youngest) subfamilies^20^. *Alu* elements have been classified into three main subfamilies including *AluJ* (oldest), *AluS* (intermediate), and *AluY* (youngest)^18^. The ability to transposition has been lost in the oldest subfamilies of both LINE-1 and *Alu*, whereas the intermediate and young subfamilies (L1PA, L1H, *AluS*, and *AluY*) are active and capable of jumping^21^.

Current evidence suggests that aberrant DNAm of LINE-1 and *Alu* elements links to several diseases: ASD^22^, pre-symptomatic dementia in type 2 diabetes^23^, and chronic lymphocytic leukemia^24^. Whole-genome sequencing investigation of the brains of individuals with ASD revealed that LINE-1 and *Alu* elements have a larger number of insertions than in normal brain tissues^25^. The binding of methyl-CpG binding protein 2 (MeCp2), transcriptional repressor, to the LINE-1 promoter was dramatically reduced, and this was related to LINE-1 overexpression in ASD brains^26^. The functional impact of LINE-1 and *Alu* elements in the ASD is currently unknown. One possibility is that LINE-1 and *Alu* elements act as enhancers or alternative promoters for host genes. Our recent study discovered associations between LINE-1/*Alu* elements and gene expression in blood transcriptome, implying that LINE-1 and *Alu* may influence the expression of host genes in ASD^22,27^. Additionally, we also found changes in global methylation of LINE-1 and *Alu* elements in the lymphoblastoid cell line of the ASD subgroup based on clinical phenotypes^22,27^. According to a recent study using blood samples from ASD, one of CpG sites within the LINE-1 sequence showed a slight decrease of methylation levels in ASD compared to unaffected controls but its methylation level was highly significant in ASD with mental regression^28^. However, locus- and family-specific methylation patterns of LINE-1 and *Alu* elements in ASD whole blood have not been reported.

Here, we intended to investigate the DNAm profiles of LINE-1 and *Alu* elements, as well as their associations with the expression of genes located nearby these elements. Using Illumina Infinium 450 K annotation, CpG sites mapping to LINE-1 and *Alu* families were identified from DNAm data (GSE80017, GSE113967, and GSE131706) obtained from the NCBI Gene Expression Omnibus (GEO) database. Differential methylation of LINE-1 and *Alu* elements was examined in a total, locus- and subfamily-specific manner for each ASD variant, including heterogeneous ASD (n = 52), ASD with 16p11.2 del (n = 7), and ASD with *CHD8* variants (n = 15). Biological functions and interactome networks of genes located nearby LINE-1 and *Alu* elements were predicted by ingenuity pathway analysis (IPA). We subsequently identified these genes that were reproducibly differentially expressed in transcriptome data obtained from multiple ASD cohorts.

## Results

### DNAm profile of LINE-1 and *Alu* elements in the heterogeneous ASD

**Figure 1:**
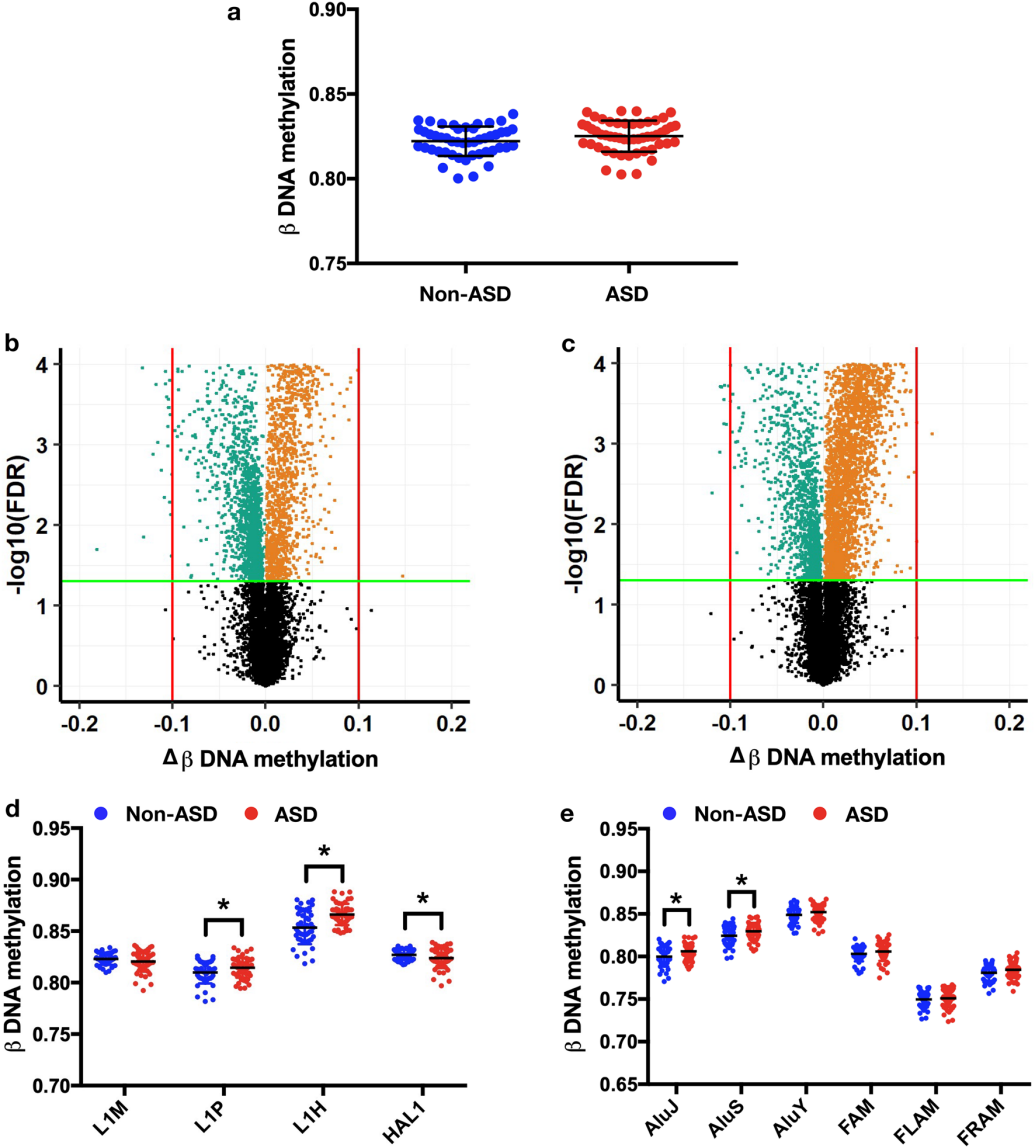
Methylation of repetitive elements (LINE-1 and *Alu*) in non-ASD (n = 48) and ASD (n = 52). Total methylation of REs (**a**). Volcano plots of mean change in methylation (Δβ) of LINE-1 (**b**) and *Alu* (**c**) against − log10 FDR-adjusted *p* value (P_FDR_) of ASD compared with non-ASD; the green line represents P_FDR_ = 0.05, the red line represents 10% of methylation changes, green dots represent hypomethylation loci, and orange dots represent hypermethylation loci. Changes in DNA methylation (Δβ) of ASD compared with non-ASD by a subfamily of LINE-1 (**d**) and *Alu* (**e**). Mean ± SD. *p < 0.05.

**Figure 2:**
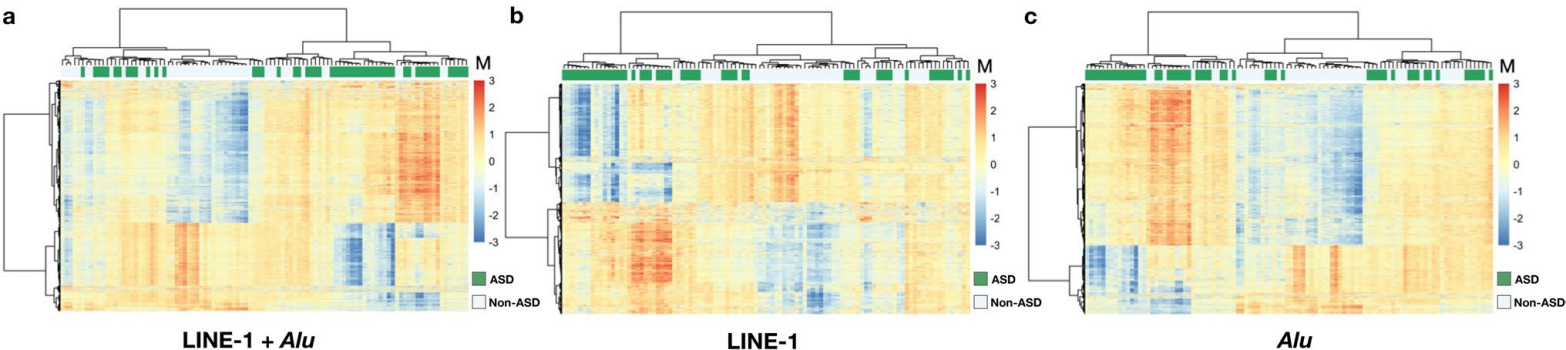
Unsupervised hierarchical cluster heatmap of the significant differentially methylated loci of the repetitive elements in non-ASD and ASD. Clustering of the 7165 significant differentially methylated loci (combining of LINE-1 and *Alu* loci) (**a**). Clustering of the 2802 significant differentially methylated loci (LINE-1) (**b**). Clustering of the 4363 significant differentially methylated loci (*Alu*) (**c**). The color scale indicates methylation level (M value), from low (blue) to high (red). Green color represents ASD and white color represents non-ASD.

A total of 22,352 probes mapping to LINE-1 and *Alu* elements were identified on the Infinium 450 K platform for differential DNAm analysis. The analyses were performed for heterogeneous ASD (n = 52) versus non-ASD (n = 48). Firstly, we measured the total methylation by combining all positions mapping to LINE-1 and *Alu* elements as the total of CpGs. In the comparison of heterogeneous ASD against non-ASD (Fig. 1a), there was no significant difference in total methylation of REs between these cohorts (Δβ = 0.003, *p* = 0.098). However, when we performed the methylation profile of REs by which LINE-1 and *Alu* positions were analyzed separately, we found that 2802 (LINE-1) and 4363 (*Alu*) differentially methylated positions (DMPs) were significantly differentially methylated (P_FDR_ < 0.05) in the heterogeneous ASD compared to non-ASD (Figs. 1b,c and 2a–c). All these loci included 2471 hypomethylated loci (LINE-1: 1437 loci, *Alu*: 1304 loci; P_FDR_ < 0.05) and 4424 hypermethylated loci (LINE-1:1365 loci, *Alu*: 3059 loci; P_FDR_ < 0.05). Due to the different activity of subfamilies of RE, LINE-1 and *Alu* elements were clustered by evolution age into three categories including old age (L1M, *AluJ*), intermediate age (L1P, L1PB, *AluS*), young age (L1HS, L1PA, *AluY*), and related (HAL1, FAM, FLAM, FRAM). The methylation of LINE-1 and *Alu* elements were changed in a subfamily-specific manner. We discovered that LINE-1 was considerably hypermethylated in young and intermediate age families, including L1H (∆β = 0.013, *p* = 0.00001) and L1P (∆β = 0.005, *p* = 0.027), but HAL1 was hypomethylated (∆β =  − 0.003, *p* = 0.03) (Fig. 1d). Methylation of *Alu* elements was significantly hypermethylated in the old and intermediate age families: *AluJ* (∆β = 0.006, *p* = 0.004) and *AluS* ($\Delta$β = 0.005, *p* = 0.016), respectively (Fig. 1e). These findings indicated that methylation of LINE-1 and *Alu* elements in the heterogeneous ASD was altered in family- and locus-specific manner rather than globally.

### LINE-1 and *Alu* methylation signatures in the homogeneous ASD (16p11.2del and *CHD8* variants)

Due to the heterogeneity in the ASD population, we also intended to investigate the methylation profile of LINE-1 and *Alu* elements in genetically homogeneous ASD, as identified in the original article of GSE113967, including ASD individuals with 16p11.2del (n = 7) and *CHD8* variant (n = 15). As for the results of ASD with 16p11.2del compared with non-ASD, we found no significant changes in the total methylation of REs compared with non-ASD (∆β =  − 0.002, *p* = 0.771, Supplementary Fig. S1a). However, the analyses identified 70 significantly locus-specific DMPs of REs in ASD with 16p11.2del including 27 DMPs at LINE-1 (5 hypomethylated loci, 22 hypermethylated loci, Supplementary Fig. S1b) and 43 DMPs at *Alu* elements (23 hypomethylated loci, 20 hypermethylated loci, Supplementary Fig. S1c). When LINE-1 and *Alu* positions were categorized into families, there was no significant difference in methylation of LINE-1 and *Alu* elements by family (Supplementary Fig. S1d,e).

**Figure 3:**
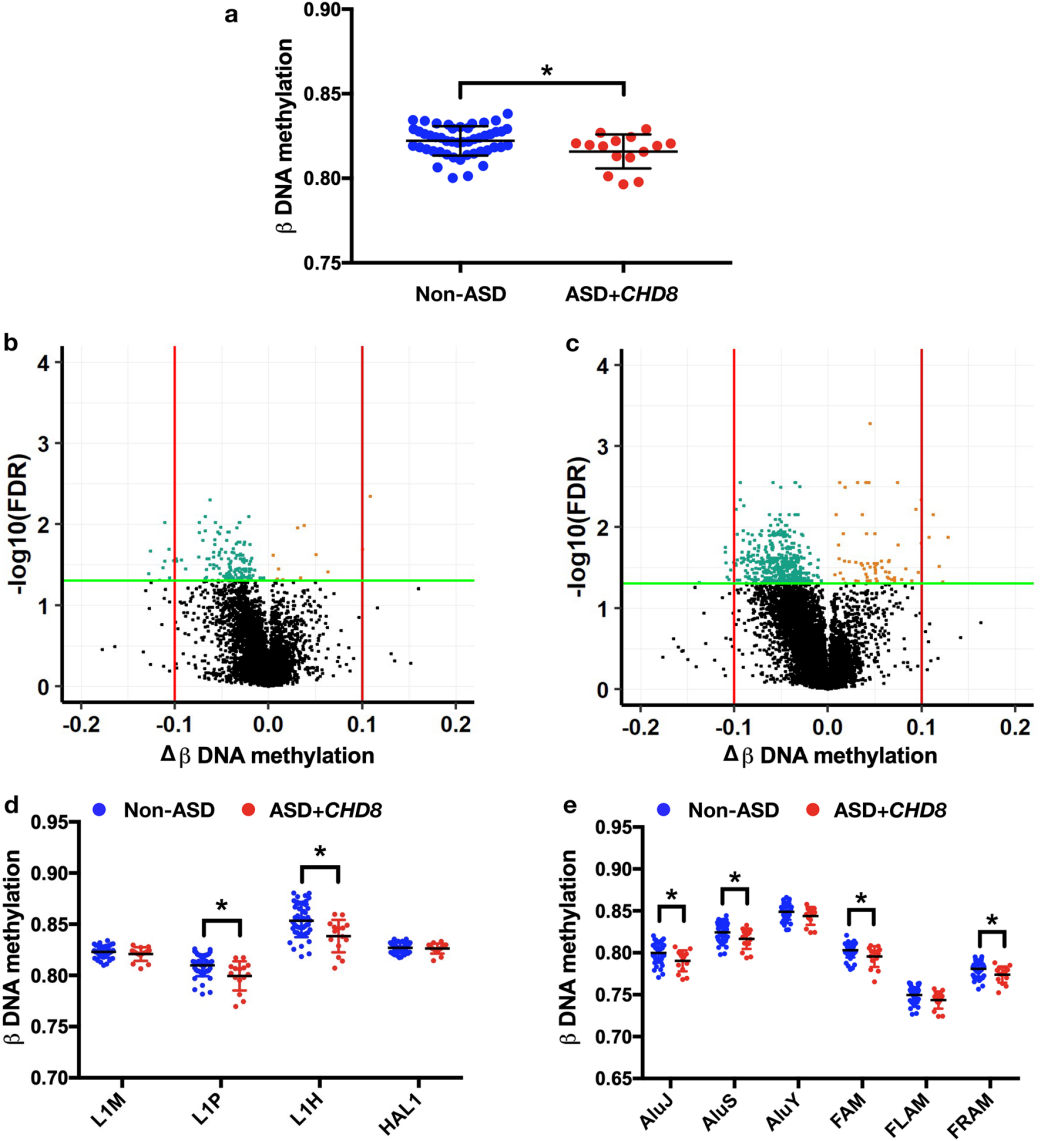
Methylation of repetitive elements (LINE-1 and *Alu*) in non-ASD (n = 48) and ASD patients who carry *CHD8* variants (n = 15). DNA methylation (**a**). Volcano plots of mean change in methylation (Δβ) of LINE-1 (**b**) and *Alu* (**c**) against − log10 FDR-adjusted *p* value (P_FDR_) of ASD compared with non-ASD; the green line represents P_FDR_ = 0.05, the red line represents 10% of methylation changes, green dots represent hypomethylation loci, and orange dots represent hypermethylation loci. Changes in DNA methylation (Δβ) of ASD with *CHD8* compared with non-ASD by a subfamily of LINE-1 (**d**) and *Alu* (**e**). Mean ± SD. *p < 0.05.

Subsequently, we analyzed data for ASD with *CHD8* variants by using the same approach. We found that total methylation of REs was exclusively hypomethylated in the ASD with *CHD8* variants (∆β =  − 0.006, *p* = 0.042, Fig. 3a). Analyzing by the position, the majority of DMPs at LINE-1 and *Alu* elements were hypomethylated (616 loci or 88.63%, P_FDR_ < 0.05) of the total identified 695 DMPs observed (Fig. 3b,c). Among all significant DMPs in the ASD with *CHD8* variants, 528 DMPs were mapped to LINE-1, while 167 DMPs were *Alu* elements. Moreover, changes in LINE-1 and *Alu* methylation regarding to their families were observed in ASD with *CHD8* variants. In contrast to the differences found in the heterogeneous ASD, young and intermediate age LINE-1 families were significantly hypomethylated including L1H (∆β =  − 0.015, *p* = 0.0038) and L1P (∆β =  − 0.010, *p* = 0.0186) (Fig. 3d). Hypomethylation of *Alu* elements was also observed in old age, intermediated age, and related families: *AluJ* (∆β =  − 0.010, *p* = 0.0154), *AluS* (∆β =  − 0.008, *p* = 0.0443), FAM (∆β =  − 0.008, *p* = 0.0399), FRAM (∆β =  − 0.007, *p* = 0.02551) respectively (Fig. 3e). These findings suggest that DNAm signatures were a widespread reduction in LINE-1 and *Alu* regions which occurred at a specific family in the ASD with *CHD8* variants but not in ASD with 16p11.2del.

### Genomic distribution of LINE-1 and *Alu* methylation in heterogeneous and homogenous ASD

To determine the differential DNAm of LINE-1 and *Alu* elements by genomic features, we performed enrichment analysis using Fisher’s exact test. CpG positions at LINE-1 and *Alu* elements were categorized to 1500 and 200 within the transcriptional start site (TSS1500 and TSS200, respectively), the 5’ untranslated region (5’UTR), the first exon (1st exon), gene body (Body), and 3’ untranslated region (3’UTR). In the heterogeneous ASD signatures, CpG sites at LINE-1 were significantly enriched in TSS1500 (*p* = 0.0005) and Body (*p* < 0.0001) (Supplementary Fig. S2a). Whereas *Alu* elements were significantly enriched in TSS1500 (*p* < 0.0001), 5’UTR (*p* < 0.0001), Body (*p* < 0.0001), and 3’UTR (*p* = 0.0086) (Supplementary Fig. S3a). However, DNAm across all retrotransposons by genomic location did not significantly differ between non-ASD and heterogeneous ASD (Supplementary Figs. S2b and S3b). DNAm signatures of the ASD with 16p11.2del and *CHD8* variants were significantly enriched in Body (*p* = 0.04) and TSS1500 (*p* < 0.0001) respectively (Supplementary Fig. S4b,c). This result shows that the changes of probes mapping to TSS1500 and gene bodies are more likely to have an association with gene expression in ASD in both heterogeneous and homogenous ASD.

### Biological functions and pathways of LINE-1 and *Alu* methylation signatures in ASD and ASD variants

To determine the biological significance of LINE-1 and *Alu* methylation signatures identified in each ASD cohort, we predicted the biological function and pathway of genes located nearby DMPs of LINE-1 and *Alu* elements using IPA software. We found that neurological diseases were significantly enriched among genes associated with LINE-1 and *Alu* methylation signatures in the heterogeneous ASD (*p* range: 0.00495–3.33E-26, 2274 genes) and ASD with *CHD8* variants (*p* range: 0.0258–0.000117, 302 genes) as shown in Supplementary Tables S1 and S2. The categories ASD and intellectual disability were exclusively associated with LINE-1 and *Alu* methylation signatures in the heterogeneous ASD (*p* = 2.56E−06, 253 genes). Whereas Huntington's disease, familial encephalopathy, and brain lesion were commonly associated with both ASD signatures. For ASD with 16p11.2del variant, LINE-1 and *Alu* methylation signatures in this cohort were significantly associated with developmental disorders (*p* range: 0.0393–0.00222, 9 genes) (Supplementary Table S3). However, only one gene was associated with the disease, possibly caused by a small number of genes associated with LINE-1 and *Alu* methylation of this ASD variant. Additionally, we discovered that several canonical pathways linked to ASD were associated with genes located nearby LINE-1 and *Alu* methylation signatures in each ASD cohort. More precisely, we found that the α-adrenergic signaling pathway was significantly associated in the heterogeneous ASD (*p* = 0.00269, 28 genes) and ASD with *CHD8* variants (*p* = 0.00646, 7 genes). Axonal guidance signaling pathway involved in nervous system development was significantly associated with LINE-1 and *Alu* methylation signatures of ASD with 16p11.2del and *CHD8* variants. These results indicate that genes associated with LINE-1 and *Alu* methylation signatures in ASD were involved with neurological diseases and ASD-comorbid disorders as well as canonical pathways known to be implicated in ASD. The list of all significant biological functions and pathways in each ASD variant is shown in Supplementary Tables S1, S2, and S3.

Interactome networks or gene regulatory networks revealed the interaction of genes located nearby LINE-1 and *Alu* methylation signatures of each ASD variant. The functions and pathways implicated in ASD were highlighted in the networks. The interactome of the heterogeneous ASD was associated with ASD and mental retardation, as well as canonical pathways implicated in ASD such as retinoic acid receptor (RAR) and AMP-activated protein kinase (AMPK) signaling (Supplementary Fig. S5). In ASD with 16p11.2del, we found that the interactome related to axonal guidance and sirtuin signaling pathway (Supplementary Fig. S6a). The interactome of ASD with *CHD8* was related to familial encephalopathy and movement disorder which conditions found in ASD individuals^29,30^. The interactomes were also associated with neuronal function including axonal guidance and synaptogenesis signaling pathways (Supplementary Fig. S6b,c).

### Identification of unique target loci located nearby LINE-1 and *Alu* signatures in heterogeneous ASD

To investigate the associations of locus-specific LINE-1 and *Alu* methylation to target gene or neighboring gene expression in the ASD, we identified DEGs from multiple ASD studies obtained from the GEO DataSets. This approach reflected the heterogeneity of the ASD population because these studies were compiled from a different ASD cohort. There were 12,419 DEGs identified from seven datasets including four studies used peripheral blood samples and three studies used post-mortem brain tissues from ASD individuals (Supplementary Table S4). We subsequently overlapped the list of DEGs with differentially methylated genes (DMGs: genes located nearby LINE-1 and *Alu* signatures). The overlapping revealed 1847 DMGs in the heterogeneous ASD that were differentially expressed in several ASD studies, with 155 of them being autism-related genes in the SFARI database. We identified 43 top DMGs, |Δβ| ≥ 5%, inversely related to gene expression, and differentially expressed in at least two studies (Supplementary Table S5). Interestingly, two of the top DMGs, potassium voltage-gated channel subfamily Q member 3 (*KCNQ3*) and ubiquitin conjugating enzyme E2 H (*UBE2H*) (Table 1), were genes in the SFARI database and were enriched in the gene regulatory network related to ASD and mental retardation (Supplementary Fig. S5).

**Table 1:** Utilized gene expression microarrays/RNA-sequencing for the differential gene expression analysis of the target genes of heterogeneous ASD.

Methylome data				Transcriptome data				
ProbeID	Subfamiliy	Delta	P_FDR_	GSE	Gene ID	Gene	log_2_FC	q-value
cg13916261	*AluSg*	− 0.100	1.05E–04	GSE59288	23,048	*FNBP1*	0.354	0.013
cg13916261	*AluSg*	− 0.100	1.05E–04	GSE42133	ILMN_1797342	*FNBP1*	0.156	0.000
cg16926147	*AluSg7*	0.062	1.31E–04	GSE18123	206573_at	*KCNQ3*	− 0.093	0.018
cg16926147	*AluSg7*	0.062	1.31E–04	GSE59288	3786	*KCNQ3*	− 0.634	0.000
cg08998414	*AluY*	− 0.106	1.85E–04	GSE64018	ENSG00000186591	*UBE2H*	0.130	0.035
cg08998414	*AluY*	− 0.106	1.85E–04	GSE42133	ILMN_1757644	*UBE2H*	0.126	0.026
cg08998414	*AluY*	− 0.106	1.85E–04	GSE25507	222419_x_at	*UBE2H*	0.171	0.023
cg23416909	L1M5	− 0.104	3.44E–04	GSE18123	206405_x_at	*USP6*	0.233	< 0.001
cg23416909	L1M5	− 0.104	3.44E–04	GSE59288	9098	*USP6*	0.614	< 0.001
cg12611243	L1MC1	0.051	2.43E–03	GSE59288	3340	*NDST1*	− 0.231	0.050
cg12611243	L1MC1	0.051	2.43E–03	GSE18123	1554010_at	*NDST1*	− 0.209	0.021
cg12611243	L1MC1	0.051	2.43E–03	GSE18123	202608_s_at	*NDST1*	− 0.275	0.029
cg24094412	L1PA3	0.023	7.01E–04	GSE59288	348,980	*HCN1*	− 0.428	0.002
cg24094412	L1PA3	0.023	7.01E–04	GSE18123	1562563_at	*HCN1*	− 0.315	0.041

The genomic regions of LINE-1 and *Alu* methylation signatures within the DMGs are shown in Supplementary Fig. S7. We identified DMRs by mapping all probes located nearby LINE-1 and *Alu* signatures using the UCSC genome browser. The findings revealed that *AluSg7* (cg16926147), which is located on the gene body of the *KCNQ3* gene (Supplementary Fig. S7a), was hypermethylated and *KCNQ3* expression level was significantly reduced in blood and post-mortem brain tissues. Interestingly, we discovered that several probes in this region, including those in the promoter region were not changed. This result suggests that LINE-1 and *Alu* methylation at DMRs may facilitate gene expression indicated by the inverse relationship between LINE-1/*Alu* methylation and gene expression. As well as *AluY* (cg08998414) within *UBE2H* gene (Supplementary Fig. S7b) and L1PA3 (cg24094412) within hyperpolarization activated cyclic nucleotide gated potassium channel 1 (*HCN1*) (Supplementary Fig. S7c), we also observed that *AluY* and L1PA3 methylation were inversely related to the gene expression levels in both blood and brain tissues of ASD cohort. Moreover, we found several DMGs that were not reported in the SFARI database but the expression of these DMGs in the blood and post-mortem brain tissues was inversely related to LINE-1 and *Alu* methylation such as *N-deacetylase and N-sulfotransferase 1* (*NDST1*) (cg12611243: L1MC1), *ubiquitin specific peptidase 6* (*USP6*) (cg23416909: L1M5), and *formin binding protein 1* (*FNBP1*) (cg13916261: *AluSg*) (Table 1). These associations suggest that DMPs at LINE-1 and *Alu* elements may affect the expression of genes located nearby these DMPs in the heterogeneous ASD cohort.

### Identification of unique target loci located nearby LINE-1 and *Alu* signatures in ASD variants

**Figure 4:**
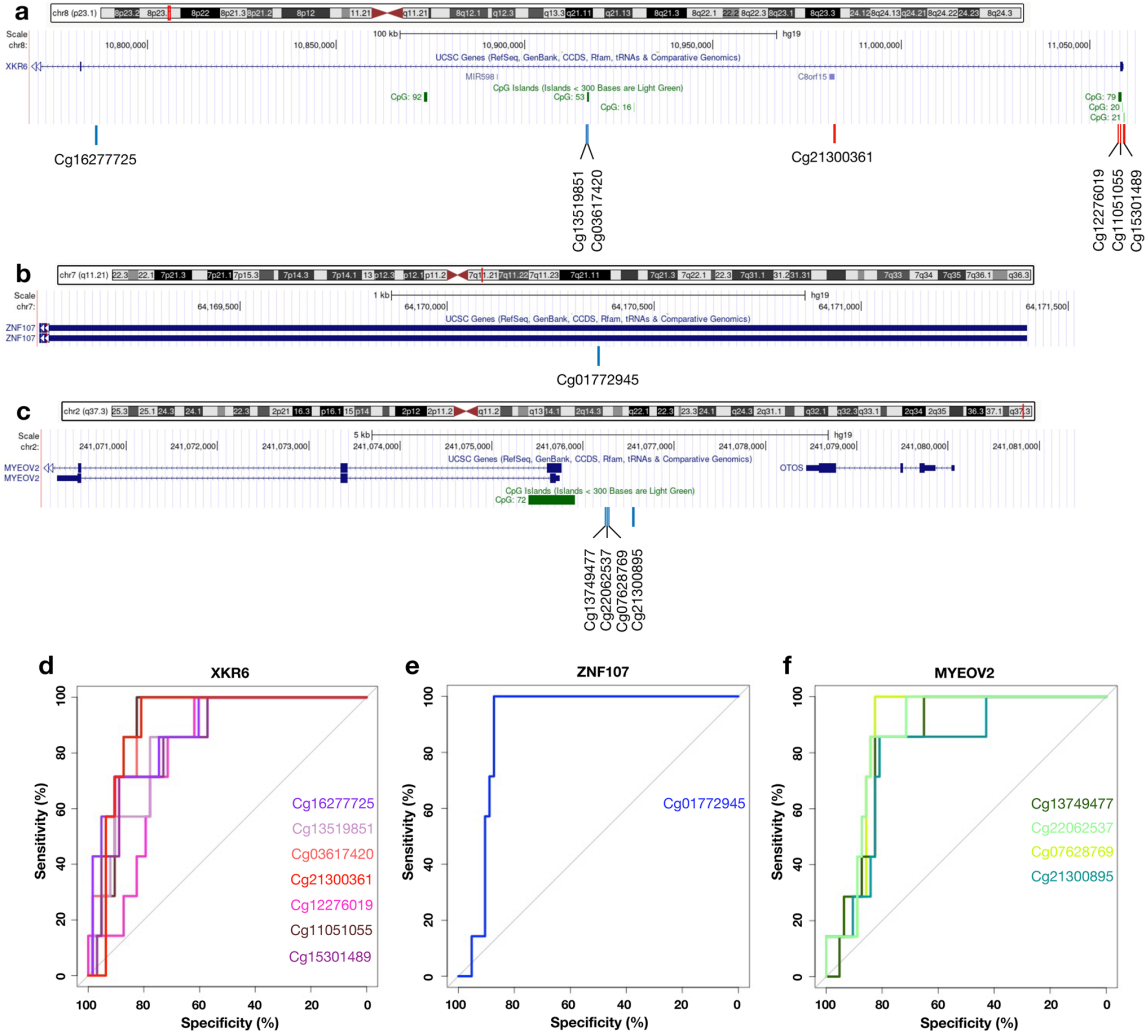
Genomic location and specificity of the unique differentially methylated regions in ASD with 16p11.2 deletion. Genomic location on *XK related 6 (XKR6)* (**a**), *Zinc Finger Protein 107 (ZNF107)* (**b**) and *Myeloma overexpressed 2 (MYEOV2)* (**c**): blue line represents hypomethylation, red line represents hypermethylation. The receiver operating characteristic (ROC) analysis of the unique differentially methylated regions of ASD with 16p11.2 deletion (n = 7) was performed against non-ASD (n = 48) and ASD with *CHD8* variants (n = 15). Specificity and sensitivity of the unique differentially methylated regions for *XKR6* (**d**), *ZNF107* (e) and *MYEOV2* (**f**).

To investigate the associations of unique LINE-1 and *Alu* methylation signatures to target gene or neighboring gene expression in the genetically homogeneous ASD, we obtained 39 and 101 DMPs that were found exclusively in the ASD with 16.p11.2 del and *CHD8* variants, respectively (Supplementary Fig. S4a, Supplementary Table S6). Within the analysis among heterogeneous ASD, ASD + 16p11.2 del and ASD + *CDH8* variants, the probes cg27005715 and cg08394597 were found in the overlap (Supplementary Fig. S4a). Furthermore, two other probes (cg26962295 and cg26620682) were found in the overlapped ASD + 16p11.2 del and ASD + CDH8 variants (Supplementary Fig. S4a). We re-analyzed them for ASD variant versus the heterogenous ASD. Next, we conducted the same strategy used for the heterogeneous ASD to select the candidate DMPs by overlapping with the transcriptome data. The overlapping of unique DMPs with transcriptome data revealed 11 and 31 unique DMGs in the ASD with 16.p11.2 del and *CHD8* variants, respectively (Supplementary Tables S7 and S8). Among the unique DMGs, we found several genes linked to neurodevelopmental disorder and ASD, including *XK related 6* (*XKR6*) (Fig. 4a)*, zinc finger protein 107* (*ZNF107*) (Fig. 4b), and *myeloma-overexpressed gene 2 protein* (*MYEOV2*) (Fig. 4c) in the ASD with 16.p11.2 del. The significant DMPs at *AluY* (cg21300361) within *XKR6* was hypermethylated, while as *AluSq* (cg01772945) within *ZNF107* and L1MB3 (cg13749477) within *MYEOV2* were hypomethylated. Interestingly, these genes were differentially expressed in the blood transcriptome of multiple ASD cohorts, and their expression was inversely relative to LINE-1 and *Alu* methylation levels (Table 2).

**Table 2:** Utilized gene expression microarrays/RNA-sequencing for the differential gene expression analysis of the target genes of ASD with 16p11.2 deletion and *CHD8* variants.

Methylome data				Transcriptome data				
Probe ID	Elements	Delta	P_FDR_	GSE	Gene ID	Gene	log_2_FC	q-value
**ASD with 16p11.2 deletion**								
cg22062537	L1MB3	− 0.079	0.0002	GSE25507	1553515_at	*MYEOV2*	0.107	0.0496
cg13749477	L1MB3	− 0.038	0.0004	GSE25507	1553515_at	*MYEOV2*	0.107	0.0496
cg07628769	L1MB3	− 0.101	0.0028	GSE25507	1553515_at	*MYEOV2*	0.107	0.0496
cg01772945	*AluSq*	− 0.048	0.0003	GSE18123	205739_x_at	*ZNF107*	0.382	0.0017
cg09168728	HAL1	0.042	0.0039	GSE18123	202651_at	*LPGAT1*	0.16	0.0038
cg21300361	*AluY*	0.024	0.0139	GSE18123	1553640_at	*XKR6*	− 0.949	0.0206
cg21300361	*AluY*	0.024	0.0139	GSE25507	1553640_at	*XKR6*	0.134	0.0112
**ASD with *CHD8* variant**								
cg06421197	*AluJo*	− 0.034	0.0266	GSE18123	206011_at	*CASP1*	0.14	0.0122
cg06421197	*AluJo*	− 0.034	0.0266	GSE42133	ILMN_2326509	*CASP1*	− 0.16	0.0258
cg06421197	*AluJo*	− 0.034	0.0266	GSE42133	ILMN_2326512	*CASP1*	− 0.17	0.0213
cg06421197	*AluJo*	− 0.034	0.0266	GSE42133	ILMN_1727762	*CASP1*	− 0.25	< 0.0001
cg06421197	*AluJo*	− 0.034	0.0266	GSE59288	834	*CASP1*	0.61	0.0096
cg09604414	*AluSx*	− 0.067	0.0336	GSE25507	229079_at	*EHMT2*	0.09	0.0471
cg22706070	L1MC5	− 0.008	0.0496	GSE25507	229079_at	*EHMT2*	0.09	0.0471
cg02169692	*AluSx*	− 0.109	0.0275	GSE42133	ILMN_3240420	*USP18*	− 0.31	0.025
cg02169692	*AluSx*	− 0.109	0.0275	GSE42133	ILMN_3240420	*USP18*	− 0.31	0.025
cg18699242	*AluSx*	− 0.106	0.0448	GSE42133	ILMN_3240420	*USP18*	− 0.31	0.025
cg18699242	*AluSx*	− 0.106	0.0448	GSE42133	ILMN_3240420	*USP18*	− 0.31	0.025

**Figure 5:**
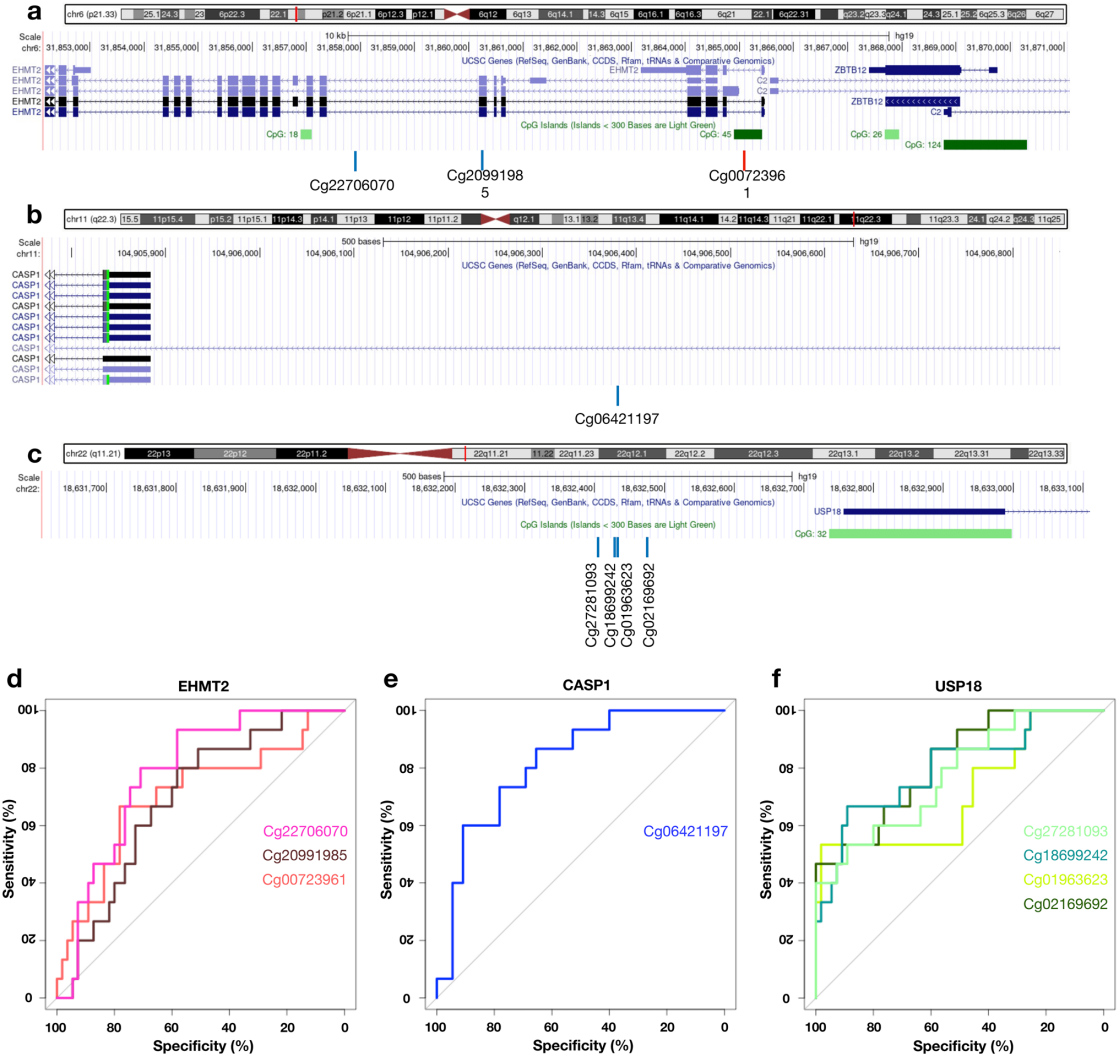
Genomic location and specificity of the unique differentially methylated regions in ASD with *CHD8* variants. Genomic location on *Euchromatic Histone Lysine Methyltransferase 2 (EHMT2)* (**a**), *Caspase 1 (CASP1)* (**b**) and *Ubiquitin Specific Peptidase 18 (USP18)* (**c**): blue line represents hypomethylation, the red line represents hypermethylation. The receiver operating characteristic (ROC) analysis of the unique differentially methylated regions of ASD with *CHD8* variants (n = 15) was performed against non-ASD (n = 48) and ASD with 16p11.2 deletion (n = 7). Specificity and sensitivity of the unique differentially methylated regions for *EHMT2* (**d**), *CASP1* (**e**) and *USP18* (**f**).

For ASD with *CHD8* variants, we found that all LINE-1 and *Alu* elements located on candidate genes were markedly hypomethylated, as expected from total and family-specific methylation levels. These DMPs consist of L1MC5 (cg22706070) within *Euchromatic Histone Lysine Methyltransferase 2* (*EHMT2*) (Fig. 5a), *AluJo* (cg06421197) within *caspase 1* (*CASP1*) (Fig. 5b), and *AluSx* (cg18699242, cg01963623, cg02169692) within *ubiquitin-specific peptidase 18* (*USP18*) (Fig. 5c). *EHMT2* was significantly increased in the blood of ASD, while *CASP1* was increased in both the blood and brain of multiple ASD cohorts (one probe was decreased). These changes were inversely relative to LINE-1 and *Alu* methylation levels within that gene (Table 2). We found that the expression of *USP18* was not inversely relative to *AluSx* methylation located on the gene. Additionally, the DMRs of *XKR6, ZNF107, MYEOV2, EHMT2,* and *CASP1* genes revealed LINE-1 and *Alu* probes as well as non-LINE-1/*Alu* probes located in the same DMRs (Figs. 4a–c and 5a,b).

### Sensitivity and specificity of unique LINE-1 and *Alu* signatures in ASD variants

To predict diagnosis of the genetically homogenous ASD by using LINE-1 and *Alu* methylation signatures, we subsequently conducted ROC curves analysis of these loci and other probes within unique DMRs to distinguish each homogenous ASD variant from non-ASD and ASD with non-specific variants. For ASD with 16.p11.2 del, *AluY* within *XKR6* (cg21300361) exhibited high sensitivity and specificity (AUC = 0.905, 95%CI = 0.83–0.98) to distinguish ASD with 16.p11.2 del from non-ASD and ASD with *CHD8* variants as shown in the ROC curves (Fig. 4d). In addition, the ROC curves of *AluSq* within *ZNF107* (cg01772945) and L1MB3 within *MYEOV2* (cg13749477) also exhibited high AUC value (*AluSq*: AUC = 0.900, 95%CI = 0.83–0.97 and L1MB3: AUC = 0.841, 95%CI = 0.74–0.95) (Fig. 4e,f). In the ASD with *CHD8* variants, LINE-1 and *Alu* methylation signatures within candidate DMGs showed moderate sensitivity and specificity as demonstrated by AUC values (AUC range: 0.712–0.819) compared with the specificity of unique loci in ASD with 16.p11.2 del, including L1MC5 (cg22706070) within *EHMT2* (Fig. 5d), *AluJo* (cg06421197) within *CASP1* (Fig. 5e), and *AluSx* (cg18699242, cg01963623, cg02169692) within *USP18* (Fig. 5f). Our findings suggest that these novel DMPs at the LINE-1 and *Alu* elements could be used for clinical purposes. However, an independent cohort is required for validation, as we were limited by the percentage of ASD individuals affected by these genetic variants.

## Discussion

Epigenetic modification is an important mechanism linking environmental and genetic factors, especially during the developmental process. There are accumulating evidences suggest that ASD heterogeneity is influenced by a combination of genetic and environmental factors^4–6,31–33^. DNA methylation status of LINE-1 and *Alu* elements can be altered in response to the environmental exposures^34,35^. Due to LINE-1 and *Alu* elements account for more than 25% of the human genome and 50% of genomic methylation^17,36^. Altered DNA methylation of these REs is involved with genomic instability and biologically relevant such as gene expression^17,37–42^. In this study, we interrogated locus- and family-specific methylation profiles of LINE-1 and *Alu* elements in ASD whole blood. In the heterogeneous ASD, we found no difference in total methylation of REs (LINE-1 and *Alu*) (Fig. 1a), which is consistent with our previous studies using lymphoblastoid cell lines that found no difference when all ASD were combined. In addition to Shpyleva’s study, total methylation of LINE-1 in the ASD brain was also not significantly altered^26^. The possibility is that the aberration of total methylation of REs may rely on family-specific REs or restrict to specific locations. Reducing the heterogeneity of ASD by classifying ASD based on clinical phenotype may be beneficial, as demonstrated by previous findings from our investigators^22,27,43–45^. Subcategorizing ASD allowed us to observe the hypomethylation of total methylation of REs in ASD with *CHD8* variants.

The aberration of LINE-1 or *Alu* elements during development may cause double-strand DNA breaks and DNA damage leading to the process of neurodegeneration^41,46^. Furthermore, identification of LINE-1 and *Alu* subfamilies has led to a better understanding of the association between the REs and human diseases because some subfamilies of LINE-1 and *Alu* elements remain active^21^. To the best of our knowledge, our study is the first to identify the locus-specific methylation at LINE-1 and *Alu* elements in a subfamily-specific manner of the ASD whole blood samples. In this study, 7165 DMPs at LINE-1 and *Alu* elements were identified in the heterogeneous ASD compared with non-ASD and the most of the DMPs were notably hypermethylated. We observed these hypermethylated loci mapped to L1P, L1H, *AluJ*, and *AluS* elements, which are intermediate and youngest LINE-1, and oldest and intermediate *Alu*, respectively. This implies that hypermethylation suppressed the most active LINE-1 and *Alu* subfamilies (intermediate and young REs) in the heterogeneous ASD. However, the hypomethylation of intermediate and young LINE-1 and *Alu* was shown in ASD with *CHD8* variants. Both hypermethylation and hypomethylation of REs may interfere with gene expression of themselves and inserted genes in ASD^22,26^.

It is important to note that LINE-1 and *Alu* elements play important roles in human brain development and brain somatic mosaicism. LINE-1 and *Alu* elements can regulate nearby genes during brain development^37,40,42,47^. LINE-1 and *Alu* retrotransposition occurred more frequently in the brain than in germline cells^48^. Furthermore, Coufal’s study, which compared LINE-1 activity in fetal neural progenitor cells (NPCs) to other somatic cells, revealed that NPCs have high retrotransposition of LINE-1^38^. They also discovered low DNAm at the LINE-1 promoter as well as a high copy number of LINE-1 in brain tissues when compared to other somatic cells^38,49^. Thus, we also performed analysis in the validation cohort using methylation data from post-mortem brain tissues of ASD including prefrontal cortex and subventricular zone regions (GSE80017 and GSE131706, respectively). We identified significant 831 and 538 significant DMPs (*p* < 0.05) at LINE-1 and *Alu* elements in the prefrontal cortex and subventricular zone of ASD, respectively. When DMPs from whole blood were compared to DMPs from post-mortem brain tissues, we found that 3.7 and 3% of DMPs in the blood of heterogeneous ASD and ASD with *CHD8* variant intersected with DMPs in ASD prefrontal cortex, respectively (Supplementary Fig. S8a,e). There are 1.4 and 0.8% of DMPs in whole blood of the heterogeneous ASD and ASD with *CHD8* variant intersected with DMPs in subventricular zone area (Supplementary Fig. S8b,f). While as significant DMPs in whole blood of ASD with 16p11.2del intersect with both prefrontal cortex and subventricular zone reached only 0.2% (Supplementary Fig. S8c,d). This warrant additional investigation into how relevant these DMPs are to ASD, and will necessitate to analyze a larger dataset of DMPs in ASD brain tissue. A validation of the data could open the way to the potential medical application of specific DMP tracking as marker to identify high risk ASD patient from blood test.

Our findings suggest that epigenetic dysregulation of LINE-1 and *Alu* elements in ASD may alter the function of autism-related genes regulated by these elements. To address this, we predicted the biological functions and networks of genes located nearby DMPs of LINE-1 and *Alu* elements. Neurological diseases and canonical pathways implicated in ASD were significantly associated with these genes (Supplementary Table S1). Moreover, interactome networks associated with ASD revealed several autism-related genes in the SFARI database (Supplementary Fig. S5). Especially, *Alu*Sg7 (cg16926147) within *KCNQ3* gene and L1PA3 (cg24094412) within *HCN1* gene were hypermethylated and inversely related to aberrant gene expression in the blood and post-mortem brain tissues of several ASD cohort studies. Hypomethylated DMPs were also discovered in the most active *Alu* family, *AluY* (cg08998414), which is located on the *UBE2H* gene and has an inverse relationship with gene expression. *KCNQ3* encodes a protein involved in neuronal excitability; people with a de novo variant of this gene experience ASD features, and some were diagnosed with ASD^50^. *HCN1* encodes a hyperpolarization-activated cation channel that is widely expressed in the brain regions^51^. *HCN1* mutation causes epileptic encephalopathy and this mutation is associated with intellectual disability and autistic traits^52^. *UBE2H* encodes an E2 ubiquitin-conjugating enzyme family protein that is involved in the protein ubiquitination mechanism. Genetic association and screening studies have shown that this gene is present in ASD individuals^53,54^. Another interesting result is hypomethylation in the HAL1 family which was found exclusively in the heterogeneous ASD. HAL1 or half-L1 encodes only ORF1p which enhances the efficiency of their transposition, but the origin, biological properties, and subfamilies have not been well studied^55^. HAL1 subfamilies were also not well annotated in our data. However, this result warrants further research of their biological activity in the ASD.

Here, we discovered LINE-1 and *Alu* methylation signatures in these genetically homogeneous ASD (both 16p11.2 del and *CHD8* variants). In the ASD with 16p11.2del, only locus-specific changes at LINE-1 and *Alu* elements were observed (Supplementary Fig. S1). We identified unique DMPs which target genes differentially expressed in the several ASD cohort studies including *AluY* within *XKR6* (cg21300361), *AluSq* within *ZNF107* (cg01772945), and L1MB3 within *MYEOV2* (cg13749477). These genes were genetic risk variants for ASD identified in genome-wide association study (GWAS), single nucleotide polymorphisms (SNPs), and copy number variation (CNV) studies^36,56,57^. In the case of ASD with the *CHD8* variants, we observed a widespread reduction of LINE-1 and *Alu* methylation levels in total methylation and the active LINE-1 and *Alu* families (L1P, L1H, and *Alu*S). This change has far-reaching implications for even the oldest and fossil family (*AluJ* and FAM), as well as FRAM family. Furthermore, the unique LINE-1 and *Alu* methylation signatures of ASD with *CHD8* variants, such as L1MC5 (cg22706070) within *EHMT2*, *AluJo* (cg06421197) within *CASP1*, and *AluSx* (cg18699242, cg01963623, cg02169692) within *USP18*, were also hypomethylated. However, we found that these alterations are inconsistent with the heterogeneous ASD profile, in which most DMPs were hypermethylated. These results may be caused by disease-specific genetic variants of *CHD8* that is a huge difference from the ASD without any genetic variants or with undefined ones. CHD8 is a chromatin remodeling/modifier factor that plays a role in the transcription process required for brain development^11^. LINE-1 and *Alu* elements have an activation and a repressive chromatin mark that is bound by several chromatin remodeling/modifier factors^39,42,47,58^. Aberrant *CHD8* function may be leading to changes in genome-wide epigenetic marks which can affect a variety of gene regulatory mechanisms. The inverse relationship between LINE-1/*Alu* methylation and gene expression was also observed in the ASD with *CHD8* variants. *EHMT2,* located nearby L1MC5, is a histone lysine methyltransferase involved with gene activation or repression. Gene and protein expression levels of *EHMT2* were significantly increased in the post-mortem brain tissues of ASD^59,60^. *CASP1* encodes cysteine-aspartic acid protease (caspase) enzyme involving apoptosis, monocyte cell fate, and immune response^61^. *CASP1*, located nearby *AluJo*, was significantly elevated in the peripheral blood mononuclear cells of ASD^62^, as well as overexpressed in two ASD studies including blood and post-mortem brain tissues. USP18 is a protein in the ubiquitin pathway which is essential for cell cycle, cell differentiation, and proliferation^63^ and its CNV has been reported in ASD individuals^64^. In transcriptome data obtained from several ASD studies, *USP18* was significantly decreased, but not inversely related to hypomethylated positions of *AluSx* located in the upstream region of *USP18* gene*.* However, three probes (cg18699242, cg01963623, and cg27281093) at the same regions have been reported to be hypomethylated and they are the *CDH8* signature in the previous study^65^. Our findings showed that DNAm of LINE-1 and *Alu* elements, located in the target genes, are connected with ASD-related genes. Moreover, biological functions and interactome of the genes located nearby LINE-1 and *Alu* methylation signatures in the ASD cohorts were associated with neurological diseases and developmental disorders, as well as canonical pathways implicated in ASD.

Unlike genetic changes, epigenetic alterations are not recorded in the genome and cannot be identified by genome sequencing. DNAm signatures are identified by comparing the methylation patterns of affected individuals to those typically developing control individuals. Several DNAm signatures have been established, and their effectiveness is demonstrated as epigenetic markers for identifying variations of uncertain significance as pathogenic or benign^66^. Although ASD pathogenesis occurs in the brain tissue, other systems such as the immune^67^, metabolic^68^, and gastrointestinal systems^69^ are also affected in ASD individuals. DNAm in the blood is highly correlated to brain tissue samples and reflects environmental exposure^70^. The discovery of distinct LINE-1 and *Alu* methylation signatures in ASD blood outlines their clinical potential to be used as non-invasive biomarkers. We conducted ROC curves analysis to predict a sensitivity and specificity of diagnosis with ASD using unique DMPs at LINE-1 and *Alu* elements identified in the blood of ASD individuals. Our findings show that LINE-1 and *Alu* methylation can be used to identify ASD with specific variants from unaffected individuals and classify them. However, additional research is required to determine its sensitivity and specificity in large and independent ASD cohorts.

Because of the limitation of available post-mortem brain tissues for each ASD with genetic variants in publicly available datasets, our analyses were carried out using Illumina Infinium 450 K methylation array from ASD whole blood. Further research with a large number of post-mortem brain tissues and whole genome bisulfite sequencing or Epic850K array (an updated version with twice coverage) is required. There are confounding factors that may interfere the methylation data such as gender, age, and blood cell type composition. However, according to the original article, these factors have been demonstrated to have no effect on the DNAm signatures of this dataset^65^. As our study does not offer sufficient statistical power to include these covariates within the model, we cannot exclude the possibility that the observed differences in methylation could in part be linked to these factors. However, to ensure that our analysis is minimally disrupted by such factors, we performed a *Chi*-square test to determine whether sex is independent or no relationship to control, heterogeneous ASD, and ASD with variants used in our model (Pearson’s *Chi*-square *p* = 0.198), as well as age comparisons. We found no significant difference between the control group versus heterogeneous ASD or ASD with variants using the nonparametric Mann Whitney U-test (*p* = 0.691 for control vs heterogeneous ASD, *p* = 0.068 for control vs ASD with 16p11.2 del, and *p* = 0.507 for control vs ASD with *CHD8* variants). In this study, we discovered links between REs methylation and expression of target genes located nearby REs. A functional assay is required to demonstrate the functional impact of these associations in ASD. Moreover, we did not perform multiple test corrections in the analysis of the validation cohort using post-mortem brain tissues, due to the small sample size which affects the statistical analysis power. Additionally, methylome and transcriptome datasets used in our study were obtained from different cohorts. However, transcriptome data from several ASD studies may reflect the heterogeneity of ASD, and one of the ASD cohorts in our analyses is a heterogeneous group. It is important to note that changes in LINE-1 and *Alu* methylation may occur as a result of a genetic factor in the genetically homogeneous ASD.

## Conclusions

**Figure 6:**
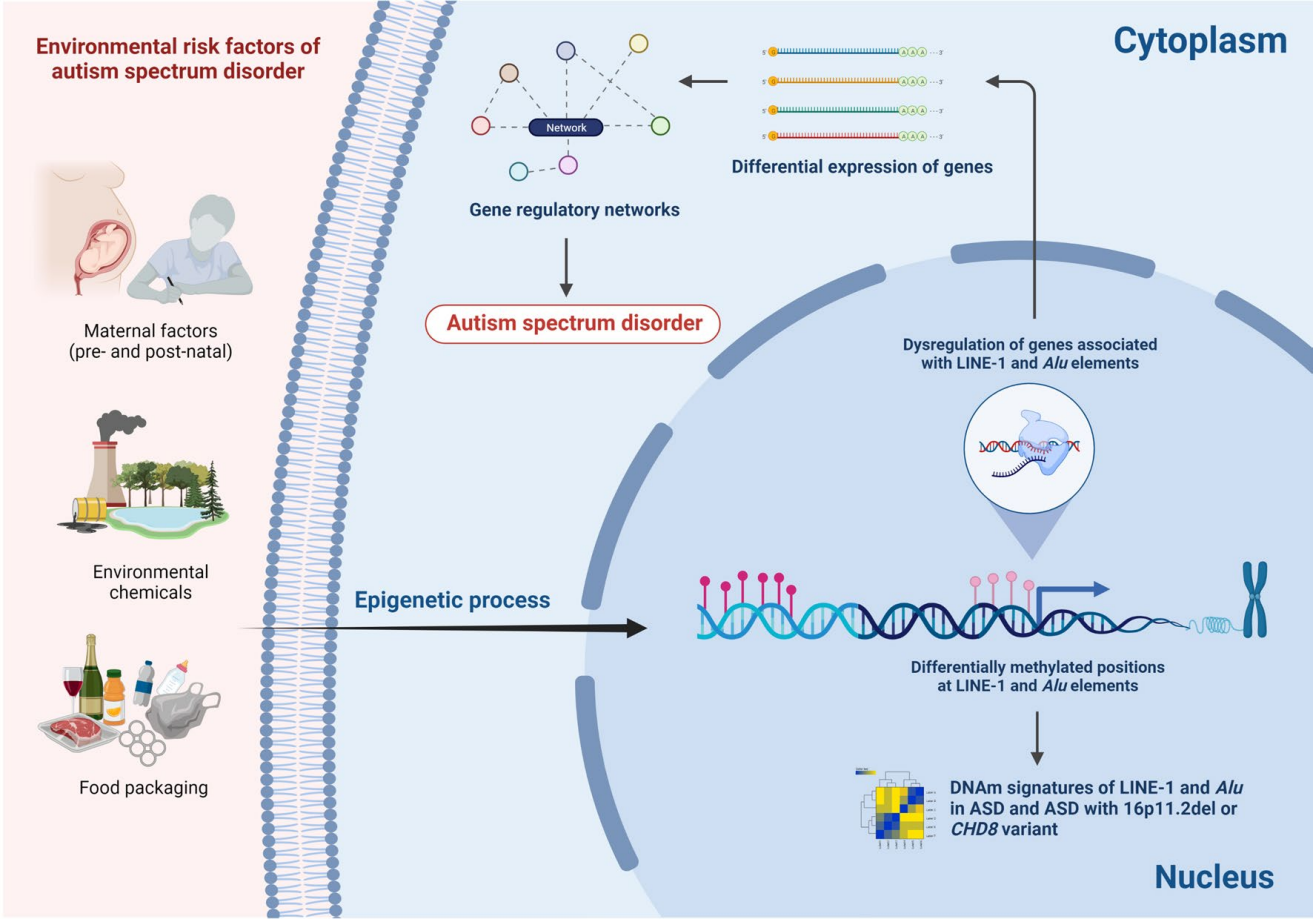
Schematic diagram illustrating a possible mechanism of LINE-1 and *Alu* elements in ASD (created with https://biorender.com/).

Locus-specific DNAm of LINE-1 and *Alu* elements in ASD, as well as its associations with gene expression, were firstly reported in our study. Our analyses revealed LINE-1 and *Alu* methylation changes in a locus- and family-specific manner which were different according to each ASD cohort. By integrating methylome and transcriptome data, the target genes of LINE-1 and *Alu* elements were identified. These target genes were differentially expressed in multiple ASD cohorts, and their functions were related to neurological diseases and developmental disorders such as ASD. Therefore, disruption of these functions may lead to ASD features (Fig. 6). Our research also demonstrated that the LINE-1 and *Alu* signatures could be applied to diagnose and classify people with ASD. Finally, our finding will provide a better understanding of the impact of LINE-1 and *Alu* elements in ASD, at least in the blood. Our study provides evidence supporting future studies on the role of LINE-1 and *Alu* related to ASD neuropathology using human post-mortem brain tissues. However, further functional studies will be necessary to investigate the subsequent impact upon the target genes and fully elucidate the role of REs in ASD biology.

## Methods

### Data collection

Differentially methylated retrotransposon loci were identified in publicly available Illumina Infinium 450 K datasets through GEO DataSets: http://www.ncbi.nlm.nih.gov/gds^71^: GSE113967^65^. In this dataset, ethical approval was granted by the Research Ethics Boards of the respective institutions (University of Michigan SickKids, Holland Bloorview Kids Rehabilitation Hospital, Western University, McMaster University)^65^. Data were collected from the heterogeneous ASD (n = 52), ASD with confirmed typical 600 Kbp deletion in 16p11.2 del (n = 7), ASD with confirmed de novo *CHD8* sequence variants (n = 15), and age-matched controls (non-ASD) (n = 48) (Supplementary Table S9). The heterogeneous ASD group in our study consists of ASD individuals who were selected without consideration for genetic characteristics or clinical symptoms as described in the original article^65^. Validation was performed in a cohort of genome-wide DNAm profiling of post-mortem brain tissue in the subventricular zone of the lateral ventricles from 17 individuals with ASD and 17 without (GSE131706)^72^ and in the frontal cortex from nine individuals with ASD and nine without (GSE80017) (Supplementary Table S9). The ethical considerations of these datasets were considered according to the original articles^72^. All methods were carried out in accordance with relevant guidelines and regulations, and the Declaration of Helsinki.

Genes with differently methylated loci were analyzed in publicly available gene expression datasets from publicly available datasets accessed via GEO using the following inclusion criteria: (1) the study must include ASD cases and controls; (2) the study must use microarray/RNA-seq technology; and (3) the study must use blood or post-mortem brain tissues. Finally, we obtained seven ASD studies, four of which used blood and three of which used post-mortem brain tissues (Supplementary Table S4).

### Differential methylation of retrotransposon subfamilies

Methylation datasets were normalized using the single-sample normalized (ssNoob) method in minfi package^73^ and corrected probes using Regression on Correlated Probes^74^. Probes on the Illumina Infinium 450 K methylation array mapping to repetitive elements (LINE-1 and *Alu*) were extracted using RepeatMarker^24^. To identify the variant-associated differential methylation of REs, probes with single nucleotide polymorphisms (SNPs) located at or within 10 base pairs of the target CpG site were included in the analysis. The CpG sites were mapped to LINE-1, *Alu*, half-L1 (HAL1), fossil *Alu* monomer (FAM), free right *Alu* monomer (FRAM), and free left *Alu* monomer (FLAM). Due to the evolution age of the REs, LINE-1 elements were clustered into oldest (L1M, mammalian-wide), intermediate (L1P, primate-specific), and youngest (L1HS, human-specific and L1PA, primate-amplified). Concomitantly, *Alu* elements were categorized into *AluJ* (oldest), *AluS* (intermediate) and *AluY* (youngest).

Mean β value across all loci of REs was calculated as total DNAm of REs in non-ASD, ASD with 16p11.2 del, and ASD with *CHD8* variants. Differential methylation of LINE-1 and *Alu* subfamilies between 1) non-ASD vs ASD, 2) non-ASD vs ASD with 16p11.2 del, and 3) non-ASD vs ASD with *CHD8* variants, were identified. DMPs to ASD were examined in the validation dataset. DMPs were identified in ASD, ASD with 16.p11.2 del, ASD with *CHD8* variants and non-ASD with 16.p11.2 del, by two-tailed t-test with correction for false discovery rate (FDR) using the Benjamini-Hochberg (BH) method^75^ and significance defined as P_FDR_ ≤ 0.05. To find the unique DMPs of each data set, the significant loci from 1 to 3 comparisons were computed to create Venn diagrams (https://bioinfogp.cnb.csic.es/tools/venny/).

### Differential gene expression analysis

The expression data of ASD studies were obtained from the GEO DataSets. The data from each study were analyzed separately using the Multiple Experiment Viewer (MeV) program (microarray software suite)^76^. Firstly, the data were filtered using a 70% cut-off filter to remove probes that were missing in > 30% of samples. The available data were then used for the identification of differentially expressed genes (DEGs) in ASD vs non-ASD cohort by using the Significance Analysis of Microarrays (SAM). The FDR and q-value less than 5% were considered as significant DEGs.

RNA-sequencing (RNA-seq) data were obtained from the Sequence Read Archive database and re-analyzed using the Galaxy platform (https://usegalaxy.org/)^77^. The quality control of RNA-seq data was assessed by *fastp* tool^78^. The cleaned reads were then mapped to the human reference genome (GRCh38/hg38) using HISAT2^79^ and quantified using the Subread package FeatureCounts^80^. Differential expression analysis was performed using the DESeq2 package^81^. The read counts were normalized using the median ratio method of the DESeq2 and the remove unwanted variation (RUV) tool^79^. The genes with a *p* value (*p*) with Benjamini–Hochberg correction of less than 0.05 were considered significant.

### Gene functions and pathway analysis

To predict biological functions and gene regulatory networks associated with LINE-1 and *Alu* elements, a list of genes located nearby DMPs of LINE-1 and *Alu* elements for each ASD variant were submitted to the Ingenuity Pathway Analysis software (IPA: QIAGEN Inc.,https://www.qiagenbioinformatics.com/products/ingenuitypathway-analysis)^82^. Gene regulatory networks were highlighted with log_2_ fold change of DNAm level.

### Identification of target loci in ASD with each genetic variant

The target RE loci of ASD with each genetic variant were identified by taking the unique DMPs from the Venn diagrams and re-analyzing the different methylation of RE loci in ASD vs ASD with 16.p11.2 del or with *CHD8* variants. We only selected loci which were significant in all three conditions (non-ASD vs ASD, ASD vs ASD with 16.p11.2 del and ASD vs ASD with *CHD8* variants) by two-tailed t-test with correction for multiple hypothesis testing using the BH method and significance defined as P_FDR_ ≤ 0.05. Moreover, the DMRs, located nearby the significantly distinct DMPs, were identified in ASD with 16.p11.2 del or with *CHD8* variants.

### Statistical analyses

Differentially methylated loci were identified by two-tailed t-tests and multiple testing correction for array data was performed by BH procedure for false discovery rate adjustment (PFDR ≤ 0.05 was considered to be significant). Fisher’s exact test was used to identify enrichment by genomic location of REs. DEGs were identified using SAM analysis with significance defined as FDR ≤ 0.05 by the BH method. Gene function and pathway analysis were performed in IPA using Fisher’s exact test with BH correction for multiple testing (P_FDR_ ≤ 0.05 was considered to be significant). All statistical analyses were performed in R (version 4.0.5) and RStudio (version 1.4.1103) using the ggplot2, plotROC, pheatmap, and GraphPad Prism (version 7.0b); data are presented as mean ± SD, and *p* ≤ 0.05 were considered to be significant.

## Supplementary Information

Supplementary Information.

## Data Availability

The datasets used and/or analyzed during the current study are available in the Gene Expression Omnibus, GSE113967, GSE131706, GSE80017, GSE59288, GSE64018, GSE28521, GSE18123, GSE25507, GSE42133, GSE89594.

## Abbreviations

16p11.2del: 16P11.2 deletion
3’UTR: 3’ Untranslated region
5’UTR: 5’ Untranslated region
ASD: Autism spectrum disorder
*CASP1*: *Caspase 1*
*CHD8*: *Chromodomain helicase DNA-binding protein 8*
CNV: Copy number variation
DEGs: Differentially expressed genes
DMGs: Differentially methylated genes
DMPs: Differentially methylated positions
DMRs: Differentially methylated regions
DNAm: DNA methylation
*EHMT2*: *Euchromatic histone lysine methyltransferase 2*
FDR: False discovery rate
*FNBP1*: *Formin binding protein 1*
GWAS: Genome-wide association study
*HCN1*: *Hyperpolarization activated cyclic nucleotide gated potassium channel 1*
*KCNQ3*: *Potassium voltage-gated channel subfamily Q member 3*
LINE-1: Long interspersed nucleotide element-1
MeCp2: Methyl-CpG binding protein 2
*MYEOV2*: *Myeloma overexpressed 2*
*NDST1*: *N-deacetylase and N-sulfotransferase 1*
NPCs: Neural progenitor cells
ROC: Receiver operating characteristic
REs: Repetitive elements
SNPs: Single nucleotide polymorphisms
TSS: Transcriptional start site
*UBE2H*: *Ubiquitin conjugating enzyme E2 H*
*USP18*: *Ubiquitin-specific peptidase 18*
*USP6*: *Ubiquitin specific peptidase 6*
*XKR6*: *XK related 6*
*ZNF107*: *Zinc finger protein 107*
