# Supplementary material for: LINE-1 and Alu methylation signatures in autism spectrum disorder and their associations with the expression of autism-related genes
Source: Sci Rep. 2022 Aug 17;12:13970. doi: 10.1038/s41598-022-18232-6 (PMC9385849; doi:10.1038/s41598-022-18232-6)
Supplement: Supplementary file 1 — Supplementary Information. [file 41598_2022_18232_MOESM1_ESM.pdf]

LINE-1 and *Alu* methylation signatures in autism spectrum disorder and their associations with the expression of autism-related genes

Thanit Saeliw[1], Tiravut Permpoon[2], Nutta Iadsee[2], Tewin Tencomnao[3,4], Valerie W. Hu[5], Tewarit Sarachana[4,6], Daniel Green[7], Chanachai Sae-Lee*[2]

[1]The Ph.D. Program in Clinical Biochemistry and Molecular Medicine, Department of Clinical Chemistry, Faculty of Allied Health Sciences, Chulalongkorn University, Bangkok, Thailand

[2]Research division, Faculty of Medicine Siriraj Hospital, Mahidol University, Bangkok, Thailand

[3]Natural Products for Neuroprotection and Anti-Ageing Research Unit, Chulalongkorn University, Bangkok, Thailand

[4]Department of Clinical Chemistry, Faculty of Allied health Sciences, Chulalongkorn University, Bangkok, Thailand

[5]Department of Biochemistry and Molecular Medicine, School of Medicine and Health Sciences, The George Washington University, Washington, DC 20052, USA

[6]SYstems Neuroscience of Autism and PSychiatric Disorders (SYNAPS) Research Unit, Department of Clinical Chemistry, Faculty of Allied Health Sciences, Chulalongkorn University, Bangkok, Thailand

[7]Institute of Life Course and Medical Sciences, Faculty of Health and Life Sciences, University of Liverpool, Liverpool, United Kingdom

*Corresponding author:

Chanachai Sae-Lee (CS), Ph.D.: 809, 8th floor, SiMR, Faculty of Medicine Siriraj Hospital, Mahidol University, Bangkok, Thailand

Tel., e-mail: +66611922353, chanachai.sae@mahidol.ac.th

E-mail Addresses of Co-authors:

TSae e-mail: 6271004937@student.chula.ac.th

TP e-mail: tiravut.perm@gmail.com

NI e-mail: nutta.i@kkumail.com

TT e-mail: tewintencomnao@gmail.com

VWH e-mail: valhu@gwu.edu

TS e-mail: tewarit.sa@chula.ac.th

DG e-mail: daniel.green@liverpool.ac.uk

Supplementary Fig. S1. Methylation of repetitive elements (LINE-1 and *Alu*) in non-ASD (n = 48) and ASD patients who carry 16p11.2 deletions (n = 7). Total DNA methylation (a). Volcano plots of mean change in methylation (Δβ) of LINE-1 (b) and *Alu* (c) against -log10 FDR-adjusted p-value (P_{FDR}) of ASD compared with non-ASD; the green line represents P_{FDR} = 0.05, the red line represents 10% of methylation changes, green dots represent hypomethylation loci, and orange dots represent hypermethylation loci. Changes in DNA methylation (Δβ) of ASD with 16p11.2 deletion compared with non-ASD by a subfamily of LINE-1 (d) and *Alu* (e). Mean ± SD.

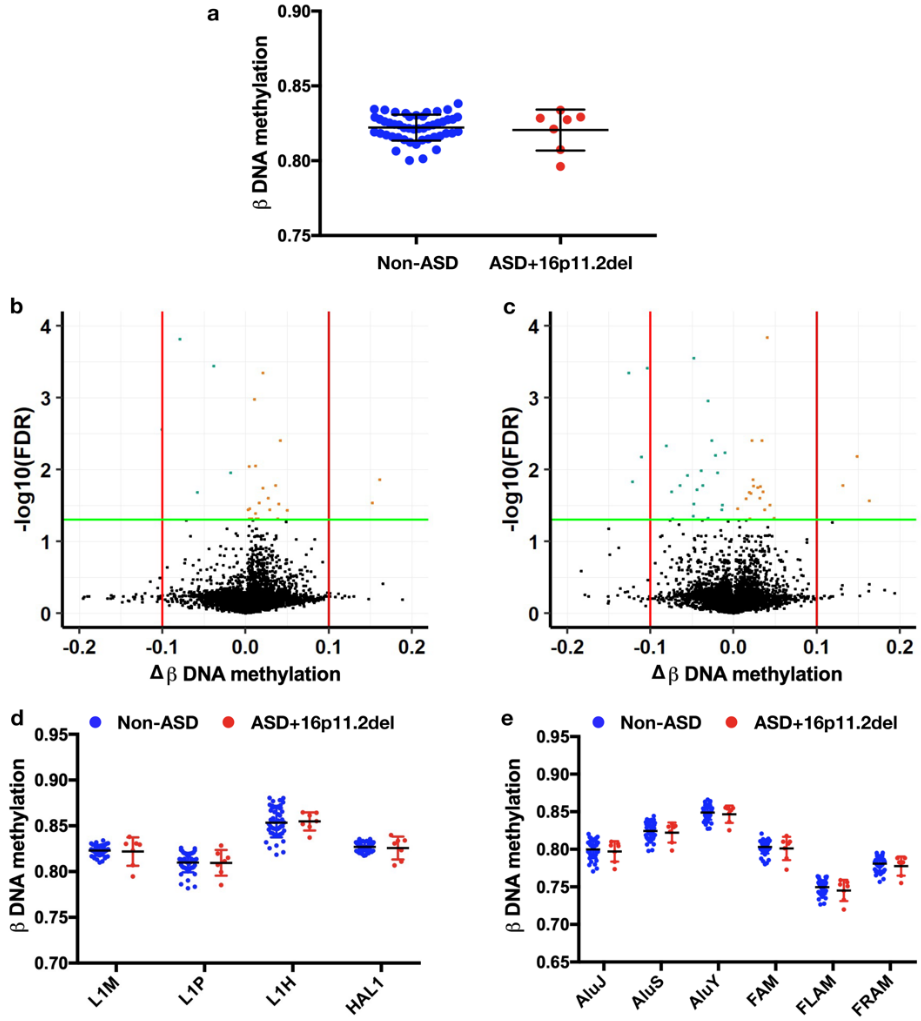

Supplementary Fig. S2. Methylation of LINE-1 elements by genomic feature in non-ASD (n = 48) and ASD (n = 52). Methylation levels (β) of LINE-1 probes by subfamily and genomic feature, as reported by the Illumina annotation (a). These probes were mapped to within 1,500 or 200 bases of the transcriptional start site (TSS1500, TSS200), the 5' untranslated region (5'UTR), first exon (1st exon), gene body (Body) and 3' untranslated region (3'UTR). The difference of methylation levels of each genomic location between ASD and non-ASD (b). Lines indicate median values, boxes the interquartile range (IQR), whiskers the highest and lowest values within 1.5×IQR, and outliers are displayed as individual points.

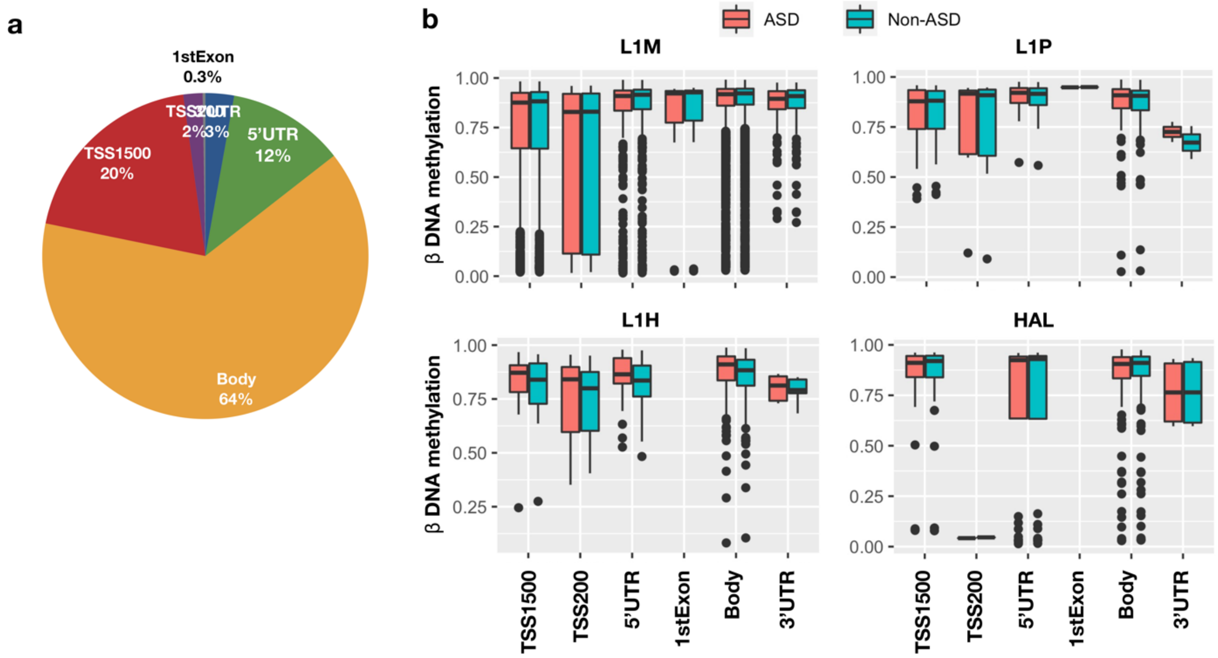

Supplementary Fig. S3. Methylation of *Alu* elements by genomic feature in non-ASD (n = 48) and ASD (n = 52). Methylation levels (β) of *Alu* probes by subfamily and genomic feature, as reported by the Illumina annotation (a). These probes were mapped to within 1,500 or 200 bases of the transcriptional start site (TSS1500, TSS200), the 5' untranslated (5'UTR), first exon (1st exon), gene body (Body) and 3' untranslated region (3'UTR). The difference of methylation levels of each genomic location between ASD and non-ASD (b). Lines indicate median values, boxes the interquartile range (IQR), whiskers the highest and lowest values within 1.5×IQR, and outliers are displayed as individual points.

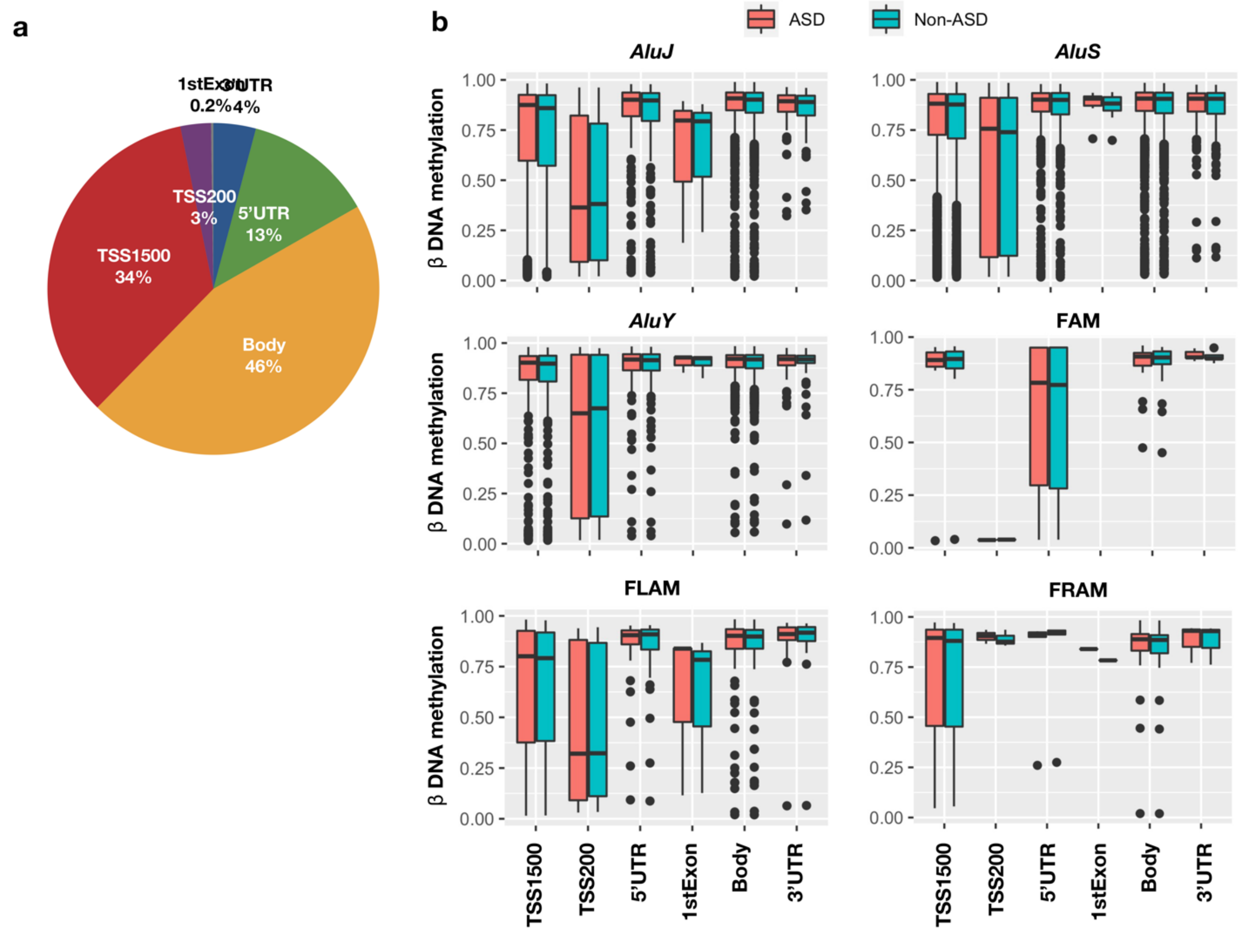

Supplementary Fig. S4. Significant differentially methylated loci of the repetitive elements. Venn diagram of the significant differentially methylated loci of the repetitive elements in ASD, ASD with 16p11.2 deletion (16p11.2 del), ASD with *CHD8* variants, and 16p11.2 del without ASD: all conditions were compared with non-ASD (a). Repartition of the unique loci of the significant differentially methylated loci of the repetitive elements by genomic feature in ASD with 16p11.2 del (b) and ASD with *CHD8* variants (c).

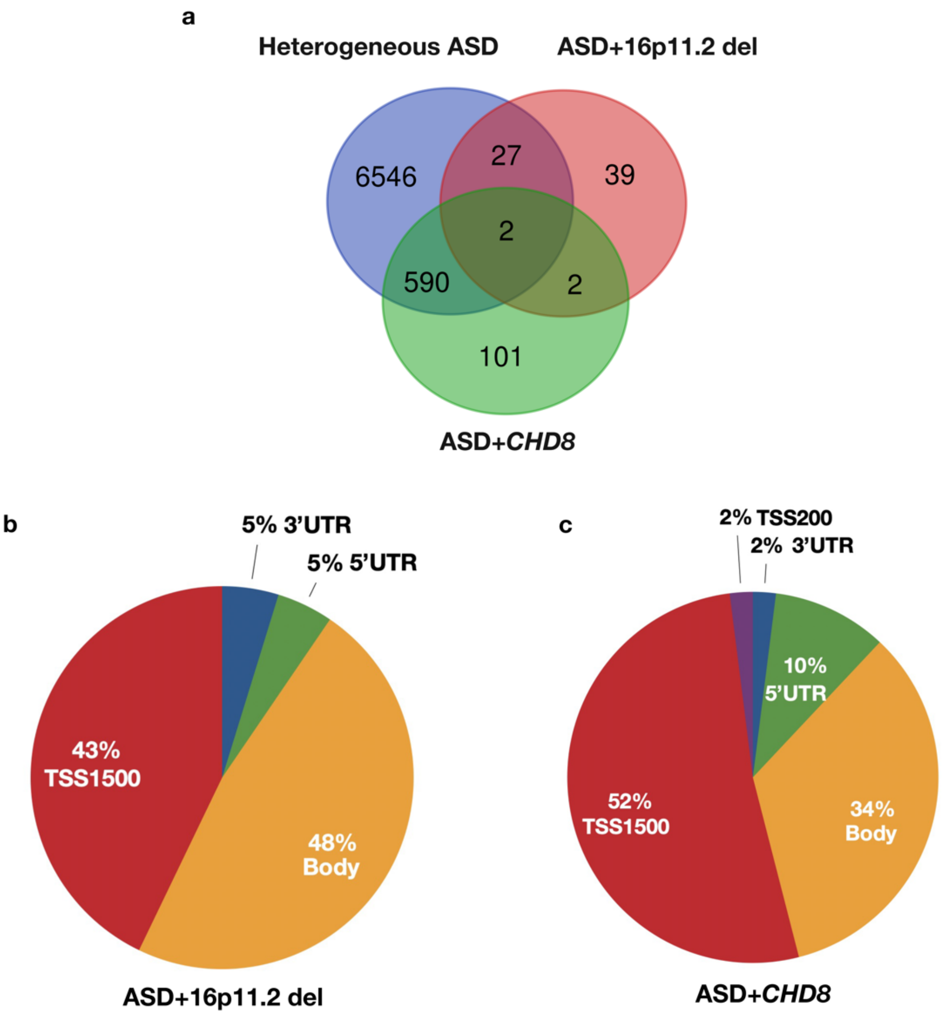

Supplementary Fig. S5. The regulatory network of differentially methylated genes (DMGs) in heterogeneous ASD that is related to neurological diseases. The gene regulatory network was predicted by ingenuity pathway analysis software using the list of DMGs (colored; red = hypermethylation; green = hypomethylation)

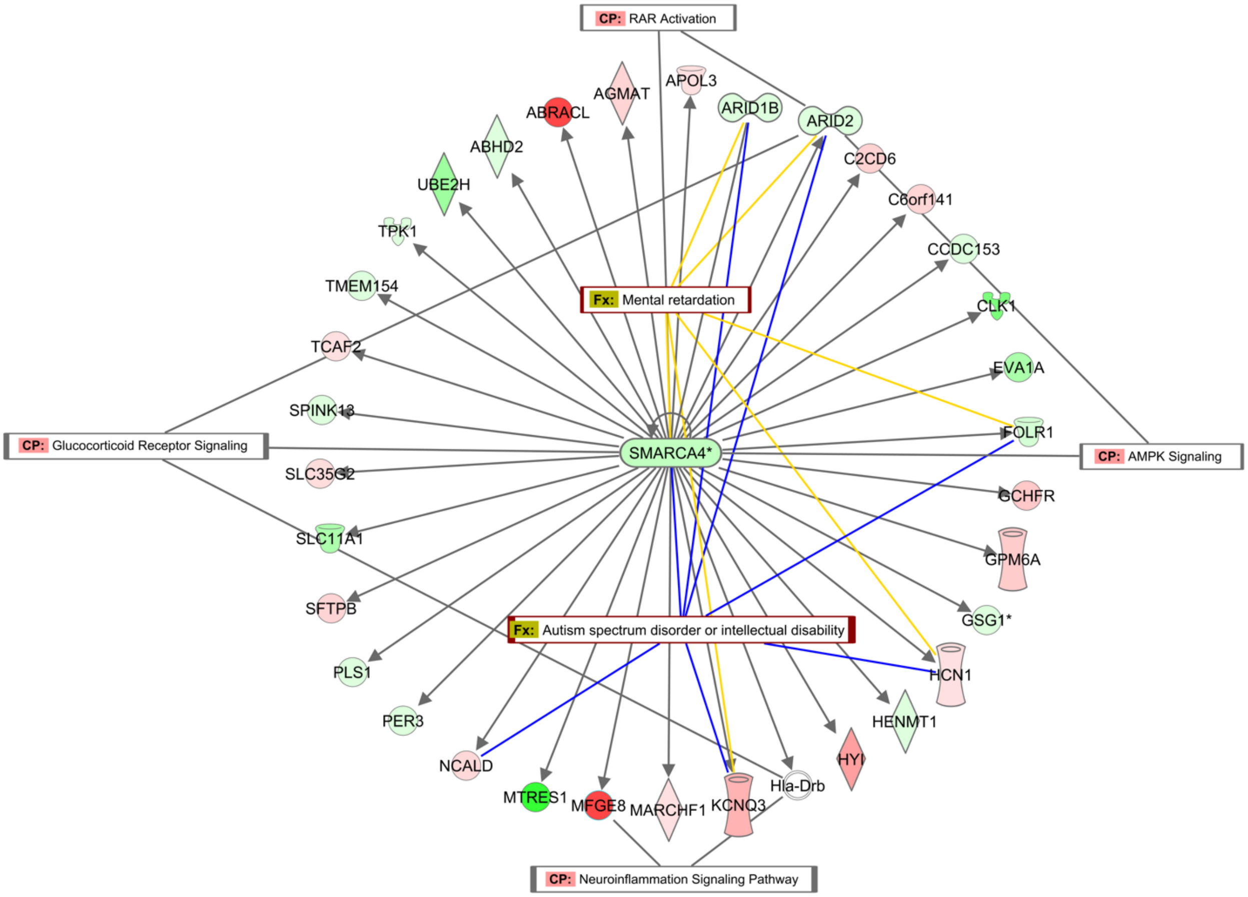

© 2000-2021 QIAGEN. All rights reserved.

Supplementary Fig. S6. Gene regulatory network of DMGs related LINE-1 and *Alu* methylation signatures. (a) Interactome of DMGs identified in ASD with 16p11.2del. (b)(c) Interactome of DMGs identified in ASD with *CHD8* variants. (Colored; red = hypermethylation; green = hypomethylation)

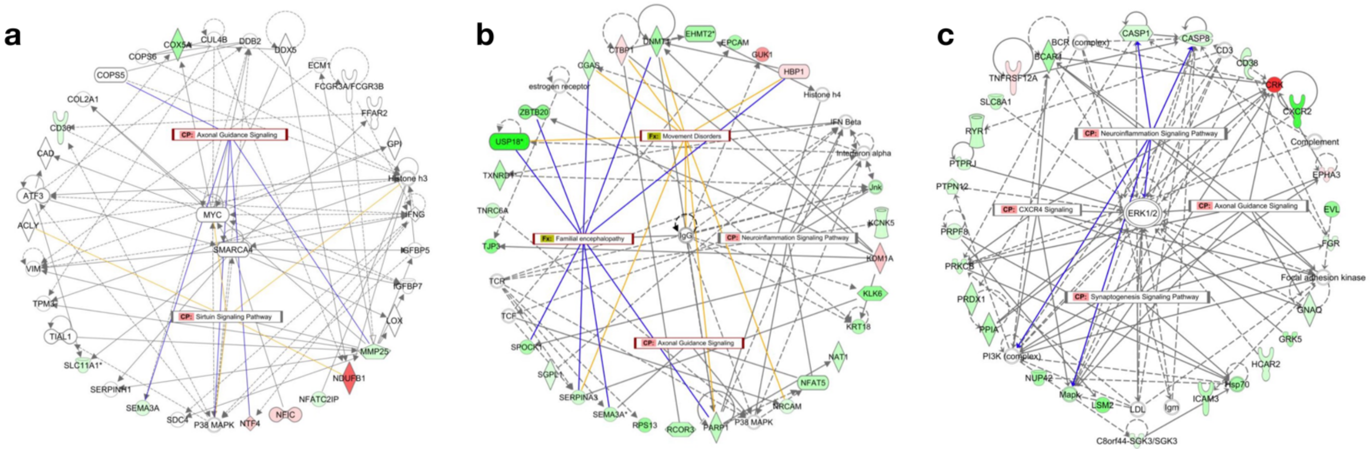

Supplementary Fig. S7. Genomic location of significant differentially methylated retrotransposons in heterogeneous ASD. Genomic location on *potassium voltage-gated channel subfamily Q member 3* (*KCNQ3*) (a), *ubiquitin conjugating enzyme E2 H* (*UBE2H*) (b), and *hyperpolarization activated cyclic nucleotide gated potassium channel 1* (*HCN1*) (c): blue line represents hypomethylation, red line represents hypermethylation.

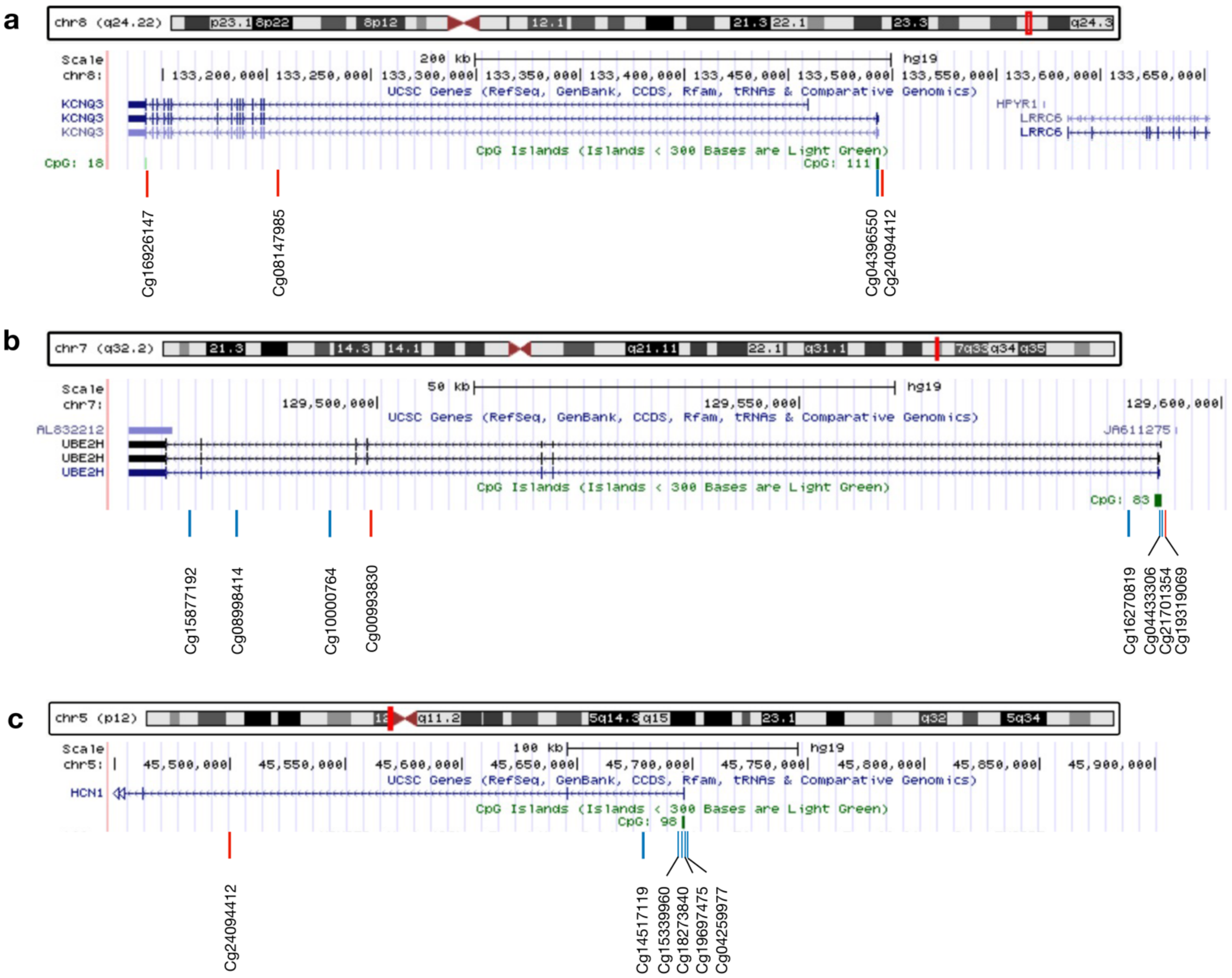

Supplementary Fig. S8. Intersection of LINE-1 and *Alu* methylation signatures between DMPs in the blood ASD and DMPs in the post-mortem brain tissues (validation cohort) including GSE80017 (prefrontal cortex) and GSE131706 (subventricular zone).

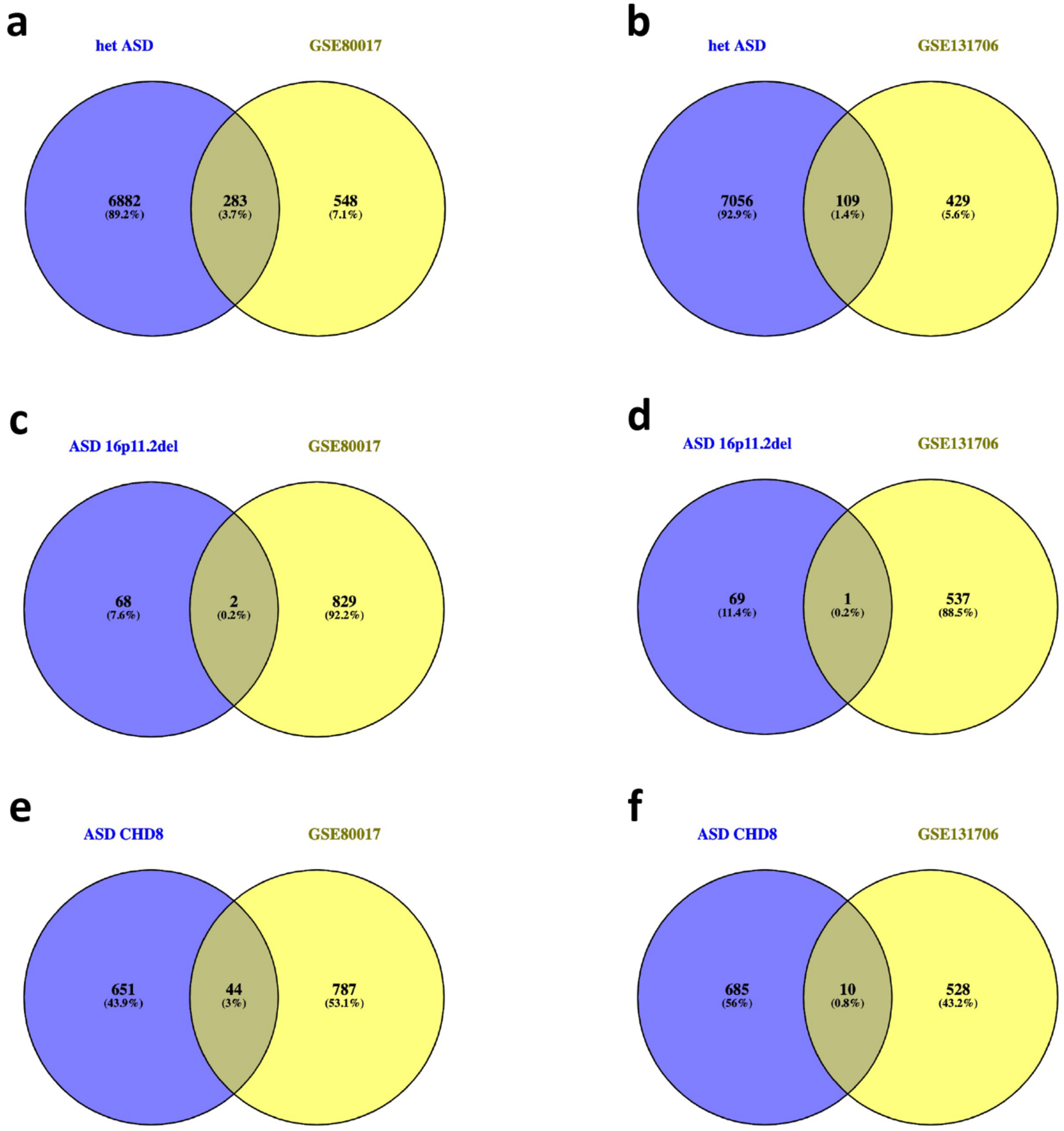

Supplementary Table S1. Diseases and biological functions associated with DMGs of LINE-1 and *Alu* methylation signatures in heterogeneous ASD predicted by the Ingenuity Pathway Analysis (IPA)

Name	P-value	#Genes
Diseases and Disorders		
Cancer	5.01E-03 - 1.01E-65	3245
Endocrine system disorders	2.27E-03 - 4.39E-52	2818
Gastrointestinal disease	3.98E-03 - 1.36E-49	2912
Reproductive system disease	2.27E-03 - 8.05E-33	2236
Neurological disease	4.95E-03 - 3.33E-26	2274
Neurological disease category		
Brain lesion	3.33E-26	1901
Familial encephalopathy	5.01E-10	471
Cerebral disorder	2.24E-09	826
Autism spectrum disorder or intellectual disability	2.56E-06	253
Huntington Disease	3.06E-06	159
Ingenuity Canonical Pathways		
Protein Kinase A Signaling	3.24E-05	92
AMPK Signaling	1.00E-03	57
ERK5 Signaling	1.32E-03	22
NGF Signaling	2.19E-03	31
α-Adrenergic Signaling	2.69E-03	28

Supplementary Table S2. Diseases and biological functions associated with DMGs of LINE-1 and *Alu* methylation signatures in ASD with *CHD8* variant predicted by the Ingenuity Pathway Analysis (IPA)

Name	P-value	#Genes
Diseases and Disorders		
Cancer	2.64E-02 - 1.17E-07	429
Immunological disease	2.11E-02 - 2.32E-06	140
Gastrointestinal disease	2.47E-02 - 2.01E-05	390
Reproductive system disease	1.06E-02 - 2.55E-04	290
Neurological disease	2.58E-02 - 1.17E-04	302
Neurological disease category		
Brain lesion	2.18E-04	253
Familial encephalopathy	2.80E-04	73
Neonatal epilepsy	1.31E-03	2
Huntington Disease	5.20E-03	26
Movement Disorders	8.28E-03	42
Ingenuity Canonical Pathways		
Pentose Phosphate Pathway (Oxidative Branch)	2.57E-03	2
Axonal Guidance Signaling	3.89E-03	20
NRF2-mediated Oxidative Stress Response	5.37E-03	11
α-Adrenergic Signaling	6.46E-03	7
CXCR4 Signaling	8.13E-03	9

Supplementary Table S3. Diseases and biological functions associated with DMGs related LINE-1 and *Alu* methylation signatures in ASD with 16p11.2del variant predicted by the Ingenuity Pathway Analysis (IPA)

Name	P-value	#Genes
Diseases and Disorders		
Hematological system development and function	3.72E-02 - 2.63E-04	4
Inflammatory response	3.93E-02 - 2.63E-04	13
Cancer	3.93E-02 - 9.29E-04	47
Developmental disorder	3.93E-02 - 2.22E-03	9
Endocrine system disorders	3.93E-02 - 2.22E-03	38
Neurological disease category		
Primary ciliary dyskinesia type 39	2.22E-03	1
Susceptibility to generalized epilepsy with febrile seizures plus type 5	2.22E-03	1
Familial advanced sleep phase syndrome type 3	2.22E-03	1
Glutaric acidemia type 2A	2.22E-03	1
Congenital fibrosis of extraocular muscles type 3A	2.22E-03	1
Ingenuity Canonical Pathways		
Axonal Guidance Signaling	4.47E-03	5
Semaphorin Signaling in Neurons	7.59E-03	2
Gustation Pathway	9.55E-03	3
mTOR Signaling	1.05E-02	3
Sirtuin Signaling Pathway	2.51E-02	3

Supplementary Table S4. Details of the gene expression profiles obtained from GEO DataSets

GSE number	Author	Sample type	Sample size	Titles	Notes
GSE59288	Liu et al.	Prefrontal cortex	ASD=34, Ctrl=38	Disruption of an Evolutionarily Novel Synaptic Expression Pattern in Autism	RNA-seq RAW data
GSE64018	Parikshak et al.	Prefrontal cortex and cerebellum	ASD=12, Ctrl=12	Genome-wide changes in lncRNA, splicing, and regional gene expression patterns in autism	Obtained DEGs list from original article
GSE28521	Voineagu et al.	Prefrontal cortex, Cerebellum, and Temporal cortex	ASD=10, Ctrl=10	Transcriptomic Analysis of Autism Brain Reveals Convergent Molecular Pathology	Microarray data
GSE18123	Kong SW et al.	Whole blood	ASD=170, Ctrl=115	Blood gene expression signatures distinguish autism spectrum disorders from controls	Microarray data
GSE25507	Alter MD et al.	Peripheral blood lymphocytes	ASD=82, Ctrl=64	Autism and increased paternal age-related changes in global levels of gene expression regulation	Microarray data
GSE42133	Pramparo T et al.	Peripheral blood Leukocyte	ASD=91, Ctrl=56	Disrupted functional networks in autism underlie early brain mal-development and provide accurate classification	Microarray data
GSSE89594	Kimura R et al.	peripheral blood	ASD=32, Ctrl=54	Integrative network analysis reveals biological pathways associated with Williams syndrome	Microarray data

Supplementary Table S5 Utilized gene expression microarrays/RNA-sequencing for the differential gene expression analysis of the target genes of heterogeneous ASD.

Methylome data				Transcriptome data				
ProbeID	Subfamiliy	Delta	PFDR	GSE	Gene ID	Gene	log2FC	q-value
cg02571470	L1MC5	0.061	1.61E-05	GSE18123	1563708_at	*SFXN5*	-0.348	0.016
cg02571470	L1MC5	0.061	1.61E-05	GSE18123	241999_at	*SFXN5*	-0.407	0.039
cg21314304	AluJb	0.054	2.68E-05	GSE25507	226298_at	*RUNDC1*	-0.141	0.027
cg21314304	AluJb	0.054	2.68E-05	GSE28521_FC	ILMN_1733875	*RUNDC1*	-0.234	0.032
cg21314304	AluJb	0.054	2.68E-05	GSE59288	146923	*RUNDC1*	-0.452	<0.001
cg04668642	AluSx	0.051	2.68E-05	GSE64018	ENSG00000166780	*C16orf45*	-0.224	0.042
cg04668642	AluSx	0.051	2.68E-05	GSE28521_FC	ILMN_1687821	*C16orf45*	-0.256	0.029
cg02616069	AluJb	0.069	3.02E-05	GSE18123	215584_at	*HECW1*	-0.259	0.007
cg02616069	AluJb	0.069	3.02E-05	GSE59288	23072	*HECW1*	-0.805	<0.001
cg15531814	AluJo	0.059	4.42E-05	GSE89594	A_23_P310257	*KLK2*	-0.161	0.018
cg15531814	AluJo	0.059	4.42E-05	GSE18123	1555545_at	*KLK2*	-0.138	0.025
cg11204311	AluJr	0.060	4.54E-05	GSE28521_FC	ILMN_1679796	*TOMM20*	-0.506	0.011
cg11204311	AluJr	0.060	4.54E-05	GSE59288	9804	*TOMM20*	-0.176	0.044
cg11204311	AluJr	0.060	4.54E-05	GSE64018	ENSG00000173726	*TOMM20*	-0.231	0.046
cg04027778	AluSc	0.054	4.66E-05	GSE28521_TC	ILMN_1712705	*RAB40C*	-0.258	0.031
cg04027778	AluSc	0.054	4.66E-05	GSE18123	227269_s_at	*RAB40C*	-0.429	0.013
cg04027778	AluSc	0.054	4.66E-05	GSE18123	1569396_at	*RAB40C*	-0.309	0.002
cg04027778	AluSc	0.054	4.66E-05	GSE59288	57799	*RAB40C*	-0.322	0.013
cg06719602	AluSx1	0.050	5.57E-05	GSE28521_FC	ILMN_1706238	*CSE1L*	-0.202	0.038
cg06719602	AluSx1	0.050	5.57E-05	GSE28521_FC	ILMN_1665797	*CSE1L*	-0.184	0.045
cg23935361	AluJb	0.066	5.82E-05	GSE42133	ILMN_1738093	*RNFT2*	-0.102	0.009
cg23935361	AluJb	0.066	5.82E-05	GSE59288	84900	*RNFT2*	-0.259	0.047
cg17429234	AluSp	0.061	6.88E-05	GSE59288	1780	*DYNC1I1*	-0.852	<0.001
cg17429234	AluSp	0.061	6.88E-05	GSE28521_FC	ILMN_1690397	*DYNC1I1*	-0.516	0.008
cg17429234	AluSp	0.061	6.88E-05	GSE64018	ENSG00000158560	*DYNC1I1*	-0.324	0.041
cg23376467	AluSq	0.077	7.13E-05	GSE18123	1556907_at	*ZNF474*	-0.292	0.035
cg23376467	AluSq	0.077	7.13E-05	GSE89594	A_33_P3360565	*ZNF474*	-0.205	0.020
cg06471678	AluYc	-0.065	7.85E-05	GSE25507	233694_at	*HSPA1L*	0.111	0.031
cg06471678	AluYc	-0.065	7.85E-05	GSE42133	ILMN_1654566	*HSPA1L*	0.107	0.030
cg15074424	AluSx1	0.083	1.01E-04	GSE28521_FC	ILMN_2212354	*WDR46*	-0.201	0.036
cg15074424	AluSx1	0.083	1.01E-04	GSE28521_TC	ILMN_2212354	*WDR46*	-0.303	0.033
cg02747612	AluSq	-0.068	1.02E-04	GSE42133	ILMN_1802053	*ZNF91*	0.162	0.016
cg02747612	AluSq	-0.068	1.02E-04	GSE18123	236128_at	*ZNF91*	0.306	0.047
cg13916261	AluSg	-0.100	1.05E-04	GSE59288	23048	*FNBP1*	0.354	0.013
cg13916261	AluSg	-0.100	1.05E-04	GSE42133	ILMN_1797342	*FNBP1*	0.156	<0.001

cg02827046	L1PA16	0.099	1.17E-04	GSE89594	A_33_P3420900	*PATE2*	-0.221	0.014
cg02827046	L1PA16	0.099	1.17E-04	GSE42133	ILMN_2133784	*PATE2*	-0.157	0.024
cg07930329	AluSx3	0.056	1.21E-04	GSE59288	10055	*SAE1*	-0.295	0.006
cg07930329	AluSx3	0.056	1.21E-04	GSE28521_TC	ILMN_1657204	*SAE1*	-0.194	0.042
cg07930329	AluSx3	0.056	1.21E-04	GSE64018	ENSG00000142230	*SAE1*	-0.194	0.039
cg16926147	AluSg7	0.062	1.31E-04	GSE18123	206573_at	*KCNQ3*	-0.093	0.018
cg16926147	AluSg7	0.062	1.31E-04	GSE59288	3786	*KCNQ3*	-0.634	<0.001
cg08499057	AluJo	0.057	1.63E-04	GSE28521_FC	ILMN_1781999	*ABCF2*	-0.164	0.043
cg08499057	AluJo	0.057	1.63E-04	GSE28521_FC	ILMN_1669201	*ABCF2*	-0.168	0.043
cg08499057	AluJo	0.057	1.63E-04	GSE28521_TC	ILMN_1669201	*ABCF2*	-0.202	0.037
cg08499057	AluJo	0.057	1.63E-04	GSE18123	207623_at	*ABCF2*	-0.321	0.005
cg08998414	AluY	-0.106	1.85E-04	GSE64018	ENSG00000186591	*UBE2H*	0.130	0.035
cg08998414	AluY	-0.106	1.85E-04	GSE42133	ILMN_1757644	*UBE2H*	0.126	0.026
cg08998414	AluY	-0.106	1.85E-04	GSE25507	222419_x_at	*UBE2H*	0.171	0.023
cg20334591	AluSx	0.055	1.85E-04	GSE42133	ILMN_2174702	*UBE2K*	-0.118	0.002
cg01996643	AluJr4	0.089	2.12E-04	GSE59288	3831	*KLC1*	-0.432	<0.001
cg01996643	AluJr4	0.089	2.12E-04	GSE42133	ILMN_1726901	*KLC1*	-0.089	0.034
cg23480284	AluJb	0.087	2.23E-04	GSE59288	8193	*DPF1*	-0.583	0.001
cg23480284	AluJb	0.087	2.23E-04	GSE18123	231599_x_at	*DPF1*	-0.228	0.029
cg23416909	L1M5	-0.104	3.44E-04	GSE18123	206405_x_at	*USP6*	0.233	<0.001
cg23416909	L1M5	-0.104	3.44E-04	GSE59288	9098	*USP6*	0.614	<0.001
cg23043218	AluJb	0.057	3.50E-04	GSE59288	84224	*NBPF3*	-0.521	0.004
cg23043218	AluJb	0.057	3.50E-04	GSE18123	1558825_s_at	*NBPF3*	-0.287	0.026
cg01779076	AluSg	-0.054	3.50E-04	GSE18123	209939_x_at	*CFLAR*	0.099	0.012
cg01779076	AluSg	-0.054	3.50E-04	GSE18123	211316_x_at	*CFLAR*	0.074	0.047
cg01779076	AluSg	-0.054	3.50E-04	GSE59288	8837	*CFLAR*	0.425	0.008
cg19453726	AluJr	0.068	4.36E-04	GSE18123	1558756_at	*C2CD3*	-0.516	0.029
cg19453726	AluJr	0.068	4.36E-04	GSE18123	231708_at	*C2CD3*	-0.379	0.018
cg07127103	AluSc	0.071	6.66E-04	GSE18123	212497_at	*MAPK1IP1L*	-0.233	0.021
cg07127103	AluSc	0.071	6.66E-04	GSE25507	225643_at	*MAPK1IP1L*	-0.154	0.036
cg26447146	AluSx	0.077	8.52E-04	GSE59288	54993	*ZSCAN2*	-0.354	0.005
cg26447146	AluSx	0.077	8.52E-04	GSE18123	231188_at	*ZSCAN2*	-0.165	0.023
cg02767673	L1MEc	-0.077	1.10E-03	GSE59288	79745	*CLIP4*	0.351	0.009
cg02767673	L1MEc	-0.077	1.10E-03	GSE18123	226425_at	*CLIP4*	0.246	0.002
cg02848097	L1MC1	-0.091	1.16E-03	GSE59288	4126	*MANBA*	0.261	0.024
cg02848097	L1MC1	-0.091	1.16E-03	GSE18123	203778_at	*MANBA*	0.222	0.002
cg01783051	L1PA10	0.053	1.96E-03	GSE18123	214170_x_at	*FH*	-0.070	0.039
cg01783051	L1PA10	0.053	1.96E-03	GSE28521_FC	ILMN_1719392	*FH*	-0.256	<0.001
cg01783051	L1PA10	0.053	1.96E-03	GSE28521_TC	ILMN_1719392	*FH*	-0.182	0.035
cg23691873	AluJr4	0.059	2.39E-03	GSE18123	206010_at	*HABP2*	-0.295	0.024
cg23691873	AluJr4	0.059	2.39E-03	GSE89594	A_23_P1173	*HABP2*	-0.180	<0.001

cg12611243	L1MC1	0.051	2.43E-03	GSE59288	3340	*NDST1*	-0.231	0.050
cg12611243	L1MC1	0.051	2.43E-03	GSE18123	1554010_at	*NDST1*	-0.209	0.021
cg12611243	L1MC1	0.051	2.43E-03	GSE18123	202608_s_at	*NDST1*	-0.275	0.029
cg00528779	L1MD3	-0.062	2.77E-03	GSE42133	ILMN_2340052	*NCOR2*	0.151	0.022
cg00528779	L1MD3	-0.062	2.77E-03	GSE42133	ILMN_1698419	*NCOR2*	0.133	0.025
cg20889774	L1PA10	0.068	3.07E-03	GSE18123	228737_at	*TOX2*	-0.282	0.027
cg20889774	L1PA10	0.068	3.07E-03	GSE59288	84969	*TOX2*	-0.324	0.018
cg21240283	L1MB5	0.074	4.93E-03	GSE28521_FC	ILMN_1777261	*FAM3C*	-0.453	0.037
cg21240283	L1MB5	0.074	4.93E-03	GSE59288	10447	*FAM3C*	-0.222	0.039
cg21240283	L1MB5	0.074	4.93E-03	GSE28521_FC	ILMN_2368773	*FAM3C*	-0.421	0.043
cg18599231	L1MA6	0.063	6.32E-03	GSE18123	1554984_a_at	*HLA-DOB*	-0.433	0.018
cg18599231	L1MA6	0.063	6.32E-03	GSE18123	205671_s_at	*HLA-DOB*	-0.212	0.013
cg13089369	L1PB1	0.057	9.66E-03	GSE28521_FC	ILMN_2340131	*MAPK10*	-0.409	0.009
cg13089369	L1PB1	0.057	9.66E-03	GSE28521_FC	ILMN_1748281	*MAPK10*	-0.347	0.006
cg12181730	AluSz	-0.058	2.38E-02	GSE18123	221645_s_at	*ZNF83*	0.345	<0.001
cg12181730	AluSz	-0.058	2.38E-02	GSE59288	55769	*ZNF83*	0.496	0.037
cg25547286	AluSz	0.062	2.82E-02	GSE59288	10426	*TUBGCP3*	-0.363	0.008
cg25547286	AluSz	0.062	2.82E-02	GSE18123	1554086_at	*TUBGCP3*	-0.140	0.021
cg01324264	AluSz	0.056	3.11E-02	GSE18123	202942_at	*ETFB*	-0.139	0.026
cg01324264	AluSz	0.056	3.11E-02	GSE42133	ILMN_1729374	*ETFB*	-0.141	<0.001
cg00507855	AluY	-0.059	4.19E-02	GSE28521_FC	ILMN_2322996	*EYA2*	0.464	0.026
cg00507855	AluY	-0.059	4.19E-02	GSE59288	2139	*EYA2*	0.538	0.024
cg00507855	AluY	-0.059	4.19E-02	GSE64018	ENSG00000064655	*EYA2*	0.535	0.013

Supplementary Table S6 List of overlapping and non-overlapping DMPs among heterogeneous ASD, ASD+16p11.2 del and ASD+*CDH8* variants.

Intersections	No. of overlapping DMPs	List of overlapping DMPs
- ASD+16p11.2 del<br>- ASD+*CHD8*<br>- Heterogeneous ASD	2	cg27005715,cg08394597
- ASD+16p11.2 del<br>- Heterogeneous ASD	27	cg13979194,cg07290865,cg21555940,cg07818063,cg25095351,cg18391611,cg22847190,cg25757017,cg01649538,cg06488671,cg00694180,cg13802355,cg05654059,cg26577993,cg17142091,cg02920701,cg13834366,cg13916261,cg17051552,cg25188165,cg26425691,cg15577272,cg25937395,cg14631503,cg24330514,cg10988561,cg22289694
- ASD+*CHD8*<br>- Heterogeneous ASD	590	cg12393411,cg00549972,cg17422409,cg23698897,cg16512795,cg05759316,cg21757392,cg20616645,cg27393573,cg14528040,cg20105448,cg17522935,cg18390494,cg08553963,cg17882033,cg19392007,cg09388237,cg05900045,cg19651268,cg18330222,cg05019180,cg21969548,cg10739132,cg21599794,cg16061177,cg14200357,cg11811959,cg08199727,cg05680926,cg02094225,cg09838962,cg24494791,cg06901762,cg14089007,cg02440947,cg09790117,cg26873488,cg13614586,cg08857906,cg18570369,cg06082293,cg12020954,cg15975375,cg25399504,cg01085853,cg21448781,cg01000036,cg07971743,cg17182002,cg23221506,cg26095478,cg05062491,cg04876223,cg25491112,cg21387117,cg19593229,cg18058157,cg19764326,cg22447396,cg23363818,cg13637352,cg03991533,cg14799489,cg26131493,cg21673929,cg08157318,cg21870518,cg20334591,cg22226852,cg20960611,cg25188141,cg01336552,cg24193797,cg25188442,cg06776823,cg02711159,cg27399297,cg15269721,cg22062131,cg04548722,cg25814432,cg16644647,cg21557451,cg24787692,cg11234875,cg09357831,cg08222555,cg06450790,cg14311225,cg15944824,cg10690082,cg23683290,cg04505313,cg08702612,cg10866856,cg10008284,cg07587783,cg04588248,cg07324609,cg26154546,cg20485361,cg16960797,cg27215140,cg03355241,cg08472753,cg26972937,cg06830415,cg11162942,cg13947513,cg19497646,cg26917965,cg00956639,cg16470963,cg07169295,cg13977583,cg12224879,cg14715098,cg12974668,cg06760144,cg09569990,cg01573175,cg02871554,cg03655147,cg25719374,cg04326562,cg16568759,cg05118364,cg06659269,cg19248797,cg10526795,cg27337803,cg23857447,cg26542912,cg07725198,cg05761535,cg13971096,cg16470306,cg11228874,cg09814912,cg21570277,cg09146645,cg01650965,cg24800365,cg04509365,cg11694026,cg01334783,cg11336323,cg02288770,cg09028316,cg11509284,cg12545097,cg10178127,cg05049638,cg21930286,cg18148726,cg06398741,cg03377789,cg19991512,cg16632575,cg02314619,cg02523938,cg19674688,cg24531568,cg23531154,cg24353651,cg10372473,cg12193153,cg19526740,cg22654601,cg05769097,cg20364241,cg13751692,cg12643877,cg24889095,cg09521141,cg00116558,cg23029185,cg01018606,cg23499616,cg10516204,cg01167408,cg12844298,cg26262710,cg20777740,cg14773480,cg21314304,

Intersections	No. of overlapping DMPs	List of overlapping DMPs
		cg09834444,cg00911267,cg09456760,cg17972930,cg07357075,cg09856869,<br>cg16415939,cg01809080,cg11423443,cg08505649,cg09267580,cg17454086,<br>cg08705895,cg20026205,cg16463100,cg22155325,cg08151866,cg11800888,<br>cg19197082,cg06686174,cg11191744,cg00995085,cg01944530,cg19615949,<br>cg26256420,cg03033780,cg27519910,cg23380262,cg19761271,cg03374552,<br>cg01435137,cg02489379,cg21262907,cg14265180,cg25477904,cg19485271,<br>cg03144715,cg04908013,cg23231093,cg01575164,cg12056677,cg26195168,<br>cg15472282,cg01540760,cg12878615,cg13453288,cg11874143,cg11660569,<br>cg11134322,cg05201677,cg15603614,cg20710898,cg14336803,cg19477926,<br>cg17812839,cg09827122,cg02968496,cg04728180,cg19045284,cg14115771,<br>cg14153625,cg22927957,cg06471678,cg18738114,cg18675361,cg02173333,<br>cg12119068,cg08865588,cg26442078,cg16205163,cg26180732,cg17097119,<br>cg06061692,cg08953850,cg00677180,cg13182809,cg09657136,cg03663072,<br>cg24620463,cg17120654,cg06610614,cg18022357,cg26238180,cg25621735,<br>cg21919903,cg17630248,cg25002551,cg08740309,cg22252462,cg02599116,<br>cg04275172,cg01324264,cg10819992,cg19918821,cg04140754,cg12794109,<br>cg03791799,cg18730307,cg15480485,cg25283205,cg18951256,cg24104623,<br>cg16926147,cg02989230,cg09112388,cg11758022,cg13619349,cg02647989,<br>cg21267836,cg12566149,cg14835746,cg00467160,cg09582822,cg27222589,<br>cg27378066,cg01548340,cg03658699,cg27360593,cg13587495,cg07507749,<br>cg27194173,cg24747539,cg01076454,cg16194684,cg05306903,cg00551654,<br>cg00572487,cg01659483,cg18637291,cg07689148,cg23743606,cg18402433,<br>cg26192872,cg14430410,cg12330727,cg09477426,cg14579098,cg07913503,<br>cg10437577,cg00591353,cg12525774,cg02695751,cg12151542,cg15695952,<br>cg01035563,cg25819914,cg27046528,cg05457768,cg15067764,cg22565361,<br>cg15495901,cg01608315,cg14959695,cg13594837,cg18828762,cg02653315,<br>cg15979146,cg11029642,cg06757863,cg07930329,cg01330679,cg05977558,<br>cg22160349,cg23817555,cg16806519,cg08163160,cg03996098,cg21543123,<br>cg00366594,cg18592742,cg06881432,cg12518775,cg24087515,cg18458116,<br>cg18231321,cg25513108,cg12765138,cg19240584,cg09604414,cg13202415,<br>cg11973587,cg00733705,cg25817973,cg01700035,cg07992633,cg13467964,<br>cg26965832,cg17210130,cg13085681,cg22608269,cg26612648,cg06864005,<br>cg02868598,cg00279778,cg08698856,cg10850863,cg21221785,cg09593727,<br>cg23707912,cg09126701,cg20971459,cg08816455,cg00858196,cg08655480,<br>cg15435907,cg00991546,cg27639249,cg21533964,cg02827046,cg11845486,<br>cg04371950,cg08384658,cg06570311,cg09120302,cg16944496,cg02581828,<br>cg14734614,cg02148292,cg09927198,cg03322872,cg07437988,cg14775487,<br>cg08164977,cg25916505,cg02600258,cg14773441,cg01165426,cg06373096,<br>cg21180845,cg07106501,cg27375966,cg13766651,cg16348962,cg05348421,<br>cg11178371,cg12282124,cg06528310,cg08068007,cg14218089,cg11827409,<br>cg04460361,cg03407622,cg04188562,cg02211160,cg12467123,cg06749259,<br>cg04671206,cg25845688,cg13111263,cg14952802,cg19470840,cg24053148,<br>cg22041446,cg23300865,cg26415023,cg19950846,cg06786000,cg04293489,<br>cg19190258,cg06485596,cg06723057,cg08064261,cg03349173,cg16732626,

Intersections	No. of overlapping DMPs	List of overlapping DMPs
		cg03043928,cg16377144,cg26780231,cg14714057,cg19605592,cg24912310,cg12911313,cg15608151,cg09249637,cg03672621,cg13227480,cg17888979,cg17284568,cg16359517,cg04410039,cg22499348,cg07690202,cg19755108,cg11661134,cg13844662,cg18331325,cg12447346,cg03728499,cg17035513,cg18311854,cg23499977,cg25421423,cg04116790,cg07521902,cg26909817,cg21301655,cg16288378,cg06321336,cg04249613,cg13942897,cg02761778,cg10894274,cg04276288,cg22933538,cg18660980,cg02023042,cg11047457,cg19789336,cg22753417,cg20126417,cg18070057,cg14933365,cg14002274,cg04576182,cg23376467,cg25169926,cg08509032,cg04784196,cg18117601,cg05388057,cg03331175,cg10987882,cg09581201,cg07779179,cg21829420,cg03584683,cg18738545,cg16241861,cg06123940,cg19853333,cg21577193,cg04611407,cg02911408,cg11826452,cg12121634,cg16399360,cg13788717,cg02126497,cg18831773,cg06196636,cg14963062,cg03965414,cg17489282,cg25731347,cg18253026,cg07596467,cg14785220,cg11204311,cg14554776,cg25523522,cg18813867,cg08023476,cg05567726,cg07680195,cg22458640,cg13796246,cg15439326,cg17762631,cg03410097,cg06033407,cg07816200,cg02448190,cg25626264,cg13390231,cg06949017,cg16655175,cg06187986,cg10515439,cg00013543,cg23656485,cg27433934,cg21251646,cg04668642,cg04466394,cg04382250,cg01390564,cg02442496,cg18874953,cg11437226,cg00261099,cg11107524,cg26453517,cg21950196,cg13681325,cg15400593,cg20024310,cg26558363,cg13880034,cg00990358,cg08867767,cg26675410,cg25045882,cg04082898,cg02018121,cg04477079,cg24027342,cg17391569,cg14027685,cg12934569,cg03330642,cg15633618,cg09427809,cg21395626,cg20594433,cg15074424
- ASD+16p11.2 del<br>- ASD+*CHD8*	2	cg26962295,cg26620682
- Heterogeneous ASD	6546	cg23027296,cg01908444,cg05956803,cg23502492,cg04922006,cg14757759,cg20889774,cg04510049,cg11857452,cg19917720,cg12738399,cg02166431,cg02605178,cg14154441,cg13324357,cg02155543,cg04626931,cg10699852,cg05187583,cg24180227,cg11668188,cg20162066,cg21160516,cg15627442,cg26114804,cg21574739,cg10600242,cg21158795,cg03603924,cg25739309,cg09713269,cg23861101,cg27167195,cg03554660,cg05119467,cg24641427,cg24751730,cg13332064,cg06314748,cg04161398,cg12890750,cg03477775,cg00171459,cg11556696,cg02867991,cg02246683,cg09892357,cg09240793,cg00311360,cg00646244,cg24806893,cg00913818,cg10346147,cg18064463,cg10900075,cg01301560,cg04915533,cg01906445,cg11060199,cg01360773,cg14558728,cg03108966,cg13695383,cg02419873,cg12589994,cg14945086,cg08239841,cg16546340,cg11089276,cg07661754,cg15559737,cg04383208,cg18191335,cg07552723,cg08351269,cg00089162,cg06369443,cg14557855,cg18042586,cg03474461,cg16915659,cg13526071,cg00219774,cg15648832,cg14143323,cg11036640,cg15056638,cg23075144,cg19846447,cg10892251,cg12595460,cg12143767,cg26020015,cg21906644,cg11262331,cg06366585,cg03130663,cg03670773,cg15294150,cg05355156,cg01837113,cg03458836,

Intersections	No. of overlapping DMPs	List of overlapping DMPs
		cg04076968,cg01527261,cg01884232,cg16301728,cg20794546,cg06819279,cg16484967,cg26494252,cg06847471,cg04217096,cg15124547,cg25993248,cg03396325,cg03640426,cg00921682,cg14166735,cg08137564,cg14364844,cg08454110,cg09526697,cg08357055,cg00656931,cg11853283,cg09380357,cg11345718,cg24034957,cg22082397,cg18145853,cg26827874,cg27502952,cg05297956,cg02402209,cg18223625,cg14865309,cg02240686,cg02969923,cg11528392,cg19360316,cg20036711,cg11126351,cg04488529,cg00134611,cg02471166,cg02987095,cg04612949,cg15789334,cg23883036,cg02521428,cg08670672,cg27111461,cg07671055,cg02057157,cg04701394,cg00688850,cg18659483,cg24157523,cg15048700,cg04889045,cg03686887,cg08334022,cg13580201,cg01023870,cg26883884,cg18180501,cg18421160,cg15944727,cg26298583,cg13018177,cg04097463,cg14469155,cg14786757,cg23250039,cg20692181,cg09529600,cg27276613,cg27121316,cg08842287,cg16868591,cg02106712,cg04818570,cg13914486,cg07324824,cg24865482,cg11415152,cg02224047,cg04534026,cg03777697,cg01276845,cg09028228,cg25680645,cg01490325,cg02770187,cg01401219,cg10268161,cg20246990,cg15498733,cg00894481,cg16117070,cg19503341,cg08648566,cg17864421,cg16001675,cg19128082,cg23249133,cg12227210,cg11952595,cg14143954,cg07572949,cg15438999,cg25365421,cg10086080,cg21473775,cg25727203,cg01573288,cg23929628,cg22424405,cg09458566,cg06053850,cg02963624,cg10948652,cg23798387,cg16345653,cg18646240,cg24589017,cg11101032,cg21756828,cg13308834,cg13126378,cg24551539,cg01380878,cg22088645,cg12565580,cg05470736,cg07739215,cg23592953,cg23016347,cg25004427,cg14045992,cg24631716,cg20724227,cg22869224,cg05430387,cg18484267,cg01098431,cg03062952,cg22564731,cg00379272,cg19007523,cg21123505,cg15891645,cg19189291,cg25758978,cg02944578,cg08174779,cg23624621,cg11238064,cg07372384,cg13943052,cg05588508,cg08394510,cg10794933,cg02456218,cg18073357,cg00836641,cg27506609,cg09784070,cg12439695,cg17362351,cg16862729,cg19788417,cg21827221,cg11779362,cg14911314,cg02044621,cg12558269,cg14305657,cg18044322,cg02854490,cg21169995,cg09514933,cg02721858,cg15721439,cg20869999,cg24426329,cg14023449,cg12079885,cg12689996,cg10590549,cg11204987,cg05489989,cg08491661,cg12824952,cg14910854,cg07429108,cg00558376,cg11247304,cg18027784,cg13258091,cg03327343,cg20242129,cg14948268,cg02848315,cg17291906,cg08539962,cg10173149,cg26850451,cg21643513,cg14333691,cg00351054,cg10240587,cg18162767,cg02768998,cg18521771,cg10107956,cg26421413,cg18356743,cg13248315,cg12210418,cg12591377,cg00150837,cg04027778,cg22958079,cg23007255,cg00415763,cg11346962,cg06767326,cg13628937,cg07399537,cg23332005,cg03148858,cg11513485,cg04899273,cg03725064,cg09834771,cg12019265,cg16027727,cg19190632,cg10425970,cg03357650,cg12198254,cg22614372,cg11209394,cg03005407,cg23201188,cg25582924,cg24924731,cg20594804,cg13715827,cg05097579,cg22605379,cg20864734,cg18497291,cg13541457,cg08769212,cg09482109,cg10620305,cg03606093,cg22772235,cg19597559,cg21376526,cg14972792,cg04309037,cg18159934,cg20646884,

Intersections	No. of overlapping DMPs	List of overlapping DMPs
		cg22634105,cg21274724,cg19239278,cg26878318,cg05644518,cg08229522,<br>cg27315695,cg10048986,cg13983495,cg07205661,cg01003176,cg13417485,<br>cg09294038,cg00112367,cg09285808,cg25625985,cg10198000,cg03526614,<br>cg23571126,cg20835555,cg01553653,cg08789741,cg08181060,cg25109031,<br>cg07450021,cg26914630,cg27228191,cg25391562,cg07060261,cg25143871,<br>cg17503596,cg06771275,cg00381789,cg27185511,cg01454540,cg01694554,<br>cg12193649,cg04390274,cg10316026,cg19540291,cg15965155,cg23196919,<br>cg24369048,cg10833829,cg10099056,cg11507713,cg25677288,cg01437450,<br>cg14768899,cg21442556,cg23484515,cg07983330,cg27373063,cg13331011,<br>cg00763190,cg26163463,cg17547157,cg10171332,cg15978191,cg18964454,<br>cg02709717,cg01915885,cg16846728,cg16601146,cg00338685,cg04693379,<br>cg05554735,cg25638229,cg25428810,cg25494536,cg01129439,cg04892672,<br>cg02959866,cg17216549,cg23972904,cg20747789,cg20638805,cg11208348,<br>cg01237677,cg13767162,cg17212500,cg12355108,cg08339324,cg21838933,<br>cg06815586,cg22129614,cg06459926,cg08289176,cg16147794,cg06663970,<br>cg23241950,cg21429544,cg09810870,cg24755177,cg13579226,cg18267124,<br>cg03634073,cg02420927,cg01326942,cg22284390,cg13812249,cg16545196,<br>cg22349487,cg10625488,cg00571067,cg23392434,cg08602694,cg24708674,<br>cg21003470,cg06059483,cg00695265,cg02824489,cg19521912,cg19329225,<br>cg20473622,cg23457357,cg25192591,cg02062970,cg26472922,cg21764101,<br>cg19911185,cg26352502,cg25275860,cg09582000,cg18989499,cg19110428,<br>cg24423329,cg00655283,cg02780120,cg16308672,cg00509057,cg07179556,<br>cg10496511,cg00423291,cg03464560,cg19517402,cg00860550,cg14182819,<br>cg13569449,cg02311588,cg15695624,cg05242653,cg16252322,cg21386099,<br>cg02990742,cg13897316,cg19887630,cg16817668,cg14224170,cg09640135,<br>cg19464037,cg19087888,cg14939553,cg24301692,cg02868222,cg16409456,<br>cg00361155,cg25927259,cg08210300,cg18589278,cg00044501,cg23226387,<br>cg26751868,cg10390329,cg04482971,cg04142312,cg05374263,cg24028459,<br>cg00167836,cg23374864,cg03686228,cg22238406,cg03870411,cg26680765,<br>cg14500563,cg08923066,cg00966078,cg07151047,cg23047079,cg04061225,<br>cg04905173,cg14099160,cg04805794,cg19666555,cg23334804,cg04773669,<br>cg10578897,cg06836020,cg11051391,cg25519921,cg12532546,cg01459748,<br>cg03804556,cg25884094,cg14953536,cg07718254,cg24611115,cg06215939,<br>cg08369295,cg27105914,cg10507156,cg00089154,cg26493814,cg23155468,<br>cg24738257,cg11724156,cg26726204,cg23509128,cg01794812,cg13096779,<br>cg00415924,cg11254196,cg18578309,cg02705940,cg16758764,cg21548096,<br>cg02800334,cg22012693,cg12098623,cg02150335,cg27382393,cg15102821,<br>cg11574130,cg02844984,cg14774273,cg22117193,cg21853296,cg10496302,<br>cg24707982,cg11325180,cg09612912,cg19638039,cg09604762,cg23268344,<br>cg00465198,cg05844402,cg21651356,cg27488741,cg02050202,cg18180230,<br>cg12041429,cg23007574,cg04084436,cg21186506,cg19828499,cg27032353,<br>cg15044252,cg05203949,cg06377825,cg09120959,cg00214780,cg06995113,<br>cg17299886,cg00166343,cg13971262,cg09269336,cg16890939,cg16209364,<br>cg09916234,cg14190196,cg22475952,cg20676413,cg22493413,cg02971604,

Intersections	No. of overlapping DMPs	List of overlapping DMPs
		cg02984966,cg24156393,cg08182409,cg12177010,cg20097407,cg16018548,cg27127202,cg05570153,cg07274300,cg14960489,cg24210426,cg05385188,cg22649290,cg00400654,cg10740203,cg07590922,cg08796647,cg10744268,cg17187041,cg05987389,cg17103026,cg00575342,cg13621925,cg12802413,cg02147805,cg22250376,cg09753762,cg06211041,cg00571466,cg17806882,cg24307117,cg27534317,cg22972187,cg22331840,cg12221729,cg27582094,cg11202620,cg09691127,cg05678871,cg25477163,cg08815261,cg21499175,cg13096307,cg08858091,cg07562687,cg20367788,cg09997119,cg14338801,cg03269429,cg24453767,cg20145601,cg00321115,cg26273127,cg21585068,cg19514148,cg02181143,cg03607054,cg05242523,cg14068721,cg24752624,cg22643337,cg00771316,cg21078247,cg15528579,cg12043511,cg21377260,cg24274354,cg10088941,cg03803150,cg02780400,cg23894755,cg10993503,cg23353281,cg21778229,cg08008141,cg14593420,cg09513647,cg07753245,cg08621277,cg00062285,cg00845626,cg26013057,cg13934560,cg15220697,cg22817650,cg05281623,cg18690271,cg05543443,cg09319020,cg26044032,cg05754632,cg13038229,cg15327998,cg20138486,cg25958261,cg07015465,cg16465369,cg02341729,cg08163798,cg08627569,cg01232121,cg14330713,cg10231500,cg02968179,cg16345272,cg03596320,cg02355043,cg06439489,cg06300012,cg16532100,cg23043218,cg05357229,cg10821976,cg01201744,cg27582696,cg00085889,cg13460465,cg25631436,cg18533237,cg07576098,cg13390361,cg15691035,cg17275695,cg03241046,cg20029657,cg08665079,cg12483357,cg23641305,cg22919686,cg19963309,cg22869634,cg11827743,cg26040729,cg04537229,cg11797265,cg24324942,cg26325497,cg19510699,cg18934822,cg02261895,cg26484916,cg07232823,cg11015356,cg06695566,cg12003990,cg20529334,cg13288599,cg24871371,cg06574139,cg05481217,cg24048806,cg07088430,cg23242649,cg11396122,cg25559103,cg12752449,cg02144870,cg04860082,cg23272987,cg03861798,cg16052972,cg04262892,cg03446092,cg05906717,cg02622542,cg26881207,cg27258878,cg18488204,cg16577533,cg11494887,cg20572967,cg05078091,cg06598386,cg00340928,cg10885089,cg15360468,cg20321498,cg14975798,cg18236358,cg21628854,cg06840895,cg11791847,cg19707638,cg05765440,cg00869962,cg25479093,cg18385457,cg15759889,cg02345638,cg08224649,cg09738193,cg13599051,cg11492288,cg23055003,cg09530179,cg23693380,cg14367513,cg25288627,cg18817246,cg07364632,cg18056738,cg25605038,cg01476138,cg17084358,cg18783135,cg01095406,cg16735509,cg26968804,cg10071277,cg01391060,cg26325163,cg15814092,cg17145196,cg12115081,cg01836858,cg24202638,cg09309979,cg11776325,cg03191384,cg20788199,cg03694795,cg14511089,cg02912476,cg24536474,cg09045787,cg06297318,cg21532334,cg03691285,cg14085247,cg23253460,cg21352393,cg18536843,cg13571582,cg10665123,cg25349265,cg13880254,cg05231930,cg22508530,cg20788115,cg06141780,cg17100390,cg10310944,cg02393535,cg16562257,cg13741103,cg26028571,cg21547845,cg14407184,cg22736267,cg25904166,cg01370111,cg01198359,cg18700146,cg09777036,cg19875535,cg20208129,cg11619648,cg09786062,cg01377475,cg25045700,cg10232190,cg21240283,cg24493120,cg24284859,

Intersections	No. of overlapping DMPs	List of overlapping DMPs
		cg18752867,cg07540629,cg08066917,cg07473590,cg22542955,cg14275273, cg11675409,cg13524563,cg25490276,cg14831745,cg17439158,cg06838584, cg07360644,cg05294259,cg09290177,cg15540749,cg14840389,cg14985591, cg00847250,cg10090044,cg09277086,cg22734427,cg08969328,cg17935677, cg09756436,cg13974798,cg24966460,cg02072104,cg08868423,cg07797421, cg15284457,cg01957971,cg17311921,cg26305717,cg09664554,cg04371703, cg21101995,cg25149391,cg13741070,cg17981254,cg18241189,cg00383735, cg08549326,cg27506139,cg17859655,cg00041865,cg08498247,cg07937578, cg10474284,cg23949901,cg16299131,cg10211725,cg09683623,cg20076039, cg24704635,cg11861406,cg15166642,cg03642066,cg06546849,cg19643315, cg03089512,cg07687820,cg16869108,cg01402948,cg24682482,cg09325695, cg18594724,cg00959027,cg16997671,cg15658344,cg21416181,cg23812948, cg03779326,cg24846791,cg03401477,cg21740507,cg21351140,cg27408262, cg04925220,cg10076508,cg23022783,cg14433428,cg11987682,cg15020247, cg10467926,cg01502484,cg11966063,cg07602776,cg20466739,cg08128444, cg08577509,cg10617396,cg25468928,cg00390120,cg16288220,cg09815235, cg22557711,cg26293920,cg25002540,cg23691873,cg04964733,cg24456094, cg20250922,cg24602093,cg21593149,cg24345166,cg12340381,cg23950767, cg00687812,cg13840590,cg11067885,cg13151361,cg26256837,cg00307501, cg16462462,cg04089197,cg07586906,cg25229198,cg19804570,cg17482273, cg12878786,cg13925235,cg20613639,cg03423957,cg06977264,cg14605001, cg25644257,cg03108250,cg03238709,cg22884082,cg11335419,cg06351029, cg16759615,cg15661251,cg15527491,cg23245898,cg24843535,cg21057349, cg00324520,cg00223863,cg23424400,cg25401621,cg20413331,cg01648784, cg22911184,cg10058885,cg08397205,cg27342505,cg23816594,cg07538838, cg27271732,cg23488395,cg05708629,cg14863101,cg20960167,cg23566468, cg13802942,cg08145495,cg20988870,cg01959405,cg06540908,cg27021666, cg17752268,cg15281406,cg25278126,cg26935729,cg04814317,cg22046143, cg11517528,cg09217023,cg15301282,cg23637167,cg08380150,cg25659288, cg26888227,cg01149053,cg26899591,cg24552802,cg02827132,cg12611243, cg13401212,cg24616366,cg05208416,cg24074054,cg26883472,cg18416823, cg22998918,cg09704152,cg14380065,cg04498159,cg27476881,cg14737263, cg16189976,cg11147497,cg08659517,cg00340744,cg24927841,cg02405484, cg06373919,cg14978741,cg16710731,cg06423617,cg24259560,cg07393618, cg06779329,cg02949523,cg14376618,cg13876071,cg07405806,cg14390884, cg03436428,cg23051632,cg27314760,cg23817265,cg17012502,cg16750112, cg05034125,cg24917883,cg19771350,cg11757672,cg16142054,cg22723249, cg06396527,cg22219933,cg17848515,cg15124343,cg10648815,cg19653701, cg00086171,cg17227941,cg02449564,cg11935592,cg19479828,cg10622684, cg04013419,cg25610960,cg16786949,cg07473599,cg02650789,cg02133456, cg08900656,cg24907569,cg27568983,cg18275339,cg01308483,cg04270402, cg00568426,cg22258490,cg03467501,cg16714273,cg23689419,cg25326090, cg05143332,cg08284758,cg06833647,cg25482228,cg18309935,cg26323746, cg04188238,cg08321145,cg20335039,cg16043230,cg01633517,cg09640718,

Intersections	No. of overlapping DMPs	List of overlapping DMPs
		cg01711850,cg09455239,cg20550536,cg17265330,cg27207764,cg15886307,cg10581754,cg19850931,cg21228642,cg20429277,cg19645839,cg03026080,cg14258645,cg25447019,cg12510271,cg07380106,cg07478893,cg23863619,cg04177517,cg27350326,cg00040486,cg21949997,cg04978451,cg00660009,cg00594793,cg00801360,cg20694666,cg16468183,cg26204448,cg16174071,cg07812459,cg05402641,cg22799069,cg05363350,cg02951457,cg05052501,cg27005906,cg14707886,cg23522840,cg12600843,cg13400284,cg14867639,cg10840054,cg08538416,cg27424057,cg04945335,cg14095321,cg23955334,cg23081204,cg22840583,cg06364293,cg22157398,cg05914369,cg13535489,cg04728073,cg18169800,cg04377849,cg07725685,cg18182475,cg11011759,cg01308806,cg14212360,cg00537709,cg16401578,cg08961047,cg02246427,cg10751914,cg14223485,cg17588491,cg09565692,cg01038517,cg08749095,cg23965109,cg14465410,cg00540819,cg21668391,cg25519056,cg11230363,cg15164048,cg24122991,cg25477334,cg24750627,cg18791823,cg01317217,cg08395822,cg11221023,cg03972852,cg23977502,cg23536509,cg00738299,cg25472172,cg05476998,cg07542043,cg18535456,cg03842980,cg18883976,cg06668647,cg20728496,cg04028541,cg05076103,cg17576104,cg27285968,cg21527414,cg07979579,cg12379755,cg27303211,cg11763017,cg12281517,cg11371953,cg06840167,cg25782778,cg21093215,cg07602317,cg03913000,cg11209001,cg27304437,cg09561458,cg11816739,cg18589846,cg03151258,cg05377587,cg03216491,cg12852407,cg06003083,cg26416766,cg02026611,cg14332139,cg14962943,cg02064831,cg19462712,cg04612349,cg10104129,cg06833721,cg04892443,cg27089146,cg01099448,cg11382976,cg08821968,cg14652369,cg08280358,cg20084815,cg23685885,cg19758385,cg26369178,cg23248208,cg10169183,cg03449621,cg15463964,cg13663416,cg17459911,cg05267740,cg15095581,cg06192220,cg08806261,cg04774364,cg14500628,cg19988798,cg26714086,cg26611926,cg00321074,cg05612506,cg20586531,cg24668728,cg12866401,cg14315334,cg15263723,cg23254737,cg26502549,cg24828975,cg06440169,cg27625887,cg02400009,cg10805645,cg05833629,cg09254350,cg02311189,cg09930167,cg26341749,cg18809463,cg00710269,cg06488615,cg05496988,cg07592136,cg03994617,cg22179905,cg08642528,cg17753475,cg13234088,cg24036612,cg07964833,cg08531025,cg15470705,cg06123714,cg13539591,cg14830815,cg08063891,cg14675896,cg08988226,cg05274179,cg00393247,cg21325040,cg06787317,cg23889301,cg11120049,cg04442311,cg05103956,cg10224939,cg24589858,cg26631127,cg04060943,cg04250180,cg21311736,cg08862717,cg21224669,cg10399850,cg20028483,cg01320920,cg25469905,cg14168059,cg24904992,cg02663175,cg10655371,cg14496314,cg19779005,cg01050005,cg04062303,cg11921411,cg10825481,cg24587796,cg08034613,cg04359302,cg03155271,cg00967405,cg18381729,cg01703966,cg18788349,cg10504120,cg02838089,cg26523866,cg24975723,cg03011822,cg19475108,cg04721719,cg11948339,cg20852378,cg13168638,cg00349636,cg01331064,cg00704176,cg06807791,cg19542548,cg00222625,cg21510284,cg17996603,cg24732062,cg25547286,cg00412526,cg08272783,cg02389050,cg14048487,cg03475756,cg00241482,cg05300773,cg06043512,

Intersections	No. of overlapping DMPs	List of overlapping DMPs
		cg18089238,cg10884633,cg10134583,cg02642833,cg24217980,cg21231853, cg20735242,cg18453188,cg03457485,cg05343548,cg15553710,cg26105731, cg20690856,cg24583410,cg21544702,cg11784214,cg04589118,cg20358075, cg23373966,cg16760291,cg14024083,cg24049084,cg05781798,cg10774367, cg26976060,cg18814048,cg11248957,cg21647407,cg03128614,cg16412148, cg27144306,cg09361751,cg18678231,cg22698031,cg04380278,cg24107165, cg16583381,cg17081998,cg19896351,cg12240828,cg14705208,cg12575429, cg20149531,cg07981140,cg22988865,cg17600233,cg23542414,cg24890976, cg05766943,cg23493751,cg26102342,cg19977683,cg22482142,cg18030084, cg02634762,cg11806305,cg20052431,cg16051599,cg26297203,cg00910319, cg13560919,cg08834459,cg02078896,cg16349724,cg27607520,cg13601636, cg00336022,cg21630243,cg02580987,cg27631638,cg15074266,cg19134770, cg11528069,cg13976610,cg20378199,cg22788109,cg16579136,cg24486867, cg22375623,cg17378387,cg14461450,cg15147841,cg22772698,cg07463167, cg00727386,cg21157357,cg24929754,cg17216301,cg06336871,cg08527226, cg14195971,cg03555579,cg01861664,cg16116663,cg02958915,cg00146334, cg04349162,cg02606575,cg21059786,cg13985868,cg05737522,cg27024083, cg01571560,cg20060185,cg18965620,cg08461425,cg24021800,cg03212228, cg26475285,cg03660331,cg04379416,cg24064325,cg04533549,cg11661235, cg03409144,cg22673336,cg14893473,cg13265607,cg17443821,cg13882046, cg19973085,cg16771862,cg01853986,cg01954057,cg18420708,cg10948220, cg25653886,cg14447733,cg19457908,cg00739139,cg04993605,cg00779304, cg22996588,cg21772589,cg16307399,cg26341003,cg12589566,cg05279382, cg18631513,cg19249583,cg20667960,cg14376110,cg14759548,cg07275480, cg15403961,cg07327927,cg09406715,cg14991562,cg05072063,cg08964756, cg07600669,cg22641542,cg00338377,cg10830021,cg16255462,cg08525476, cg12980798,cg02279591,cg19632366,cg09312279,cg16543876,cg21801085, cg01921212,cg05823818,cg00767403,cg01650612,cg03983808,cg22140583, cg09333836,cg15956049,cg27381618,cg25326998,cg05730992,cg23352067, cg24202922,cg09970332,cg14127641,cg12539194,cg06515617,cg07841529, cg02197019,cg20915907,cg04912438,cg12156012,cg00994644,cg03233224, cg24194093,cg20901993,cg15003833,cg14706226,cg03038520,cg09273289, cg20284705,cg02602268,cg10673431,cg11555640,cg09793796,cg09889228, cg23130946,cg24990022,cg26707265,cg22274522,cg11259254,cg11737081, cg24470929,cg15836199,cg18516241,cg15992711,cg10493270,cg13186010, cg09226290,cg25241823,cg08559214,cg25499769,cg25362169,cg04565216, cg02316852,cg08547873,cg05573149,cg06659875,cg08952300,cg10076958, cg24152297,cg02136723,cg13335769,cg01586011,cg14926216,cg09499747, cg26006672,cg06815630,cg13483099,cg09197288,cg03516394,cg12181730, cg09241714,cg14894369,cg13936208,cg00815484,cg07460894,cg15856832, cg00267196,cg26133909,cg19761060,cg26789319,cg17533142,cg06483795, cg23520478,cg01269913,cg13073589,cg14956546,cg23780049,cg08306333, cg15223933,cg25491247,cg26719056,cg01367751,cg06700353,cg09112262, cg12932539,cg15680557,cg25667409,cg24843157,cg16621856,cg03113271,

Intersections	No. of overlapping DMPs	List of overlapping DMPs
		cg27209175,cg18593073,cg18467551,cg16245495,cg21167587,cg11579447,cg04844516,cg18696805,cg02618000,cg16096085,cg07134622,cg04343242,cg05976571,cg06020868,cg21152662,cg10302285,cg15401058,cg08897302,cg19814520,cg20821992,cg27495951,cg18969709,cg20882571,cg17599298,cg03890634,cg24125910,cg18901378,cg18469747,cg19288514,cg25728543,cg12530864,cg18758339,cg08659785,cg15556873,cg24558112,cg16000824,cg17586419,cg01325840,cg15185244,cg03123170,cg10208083,cg11090641,cg06029376,cg12808990,cg01212680,cg00809453,cg21408734,cg21244845,cg07356283,cg12765632,cg24613956,cg16038269,cg09447749,cg20840778,cg15372862,cg04533214,cg05736906,cg19027126,cg26586942,cg03737209,cg19674611,cg16335566,cg24714100,cg27326127,cg05033560,cg10573450,cg10191404,cg24575651,cg00346673,cg24904103,cg23687958,cg13581752,cg16314538,cg10276665,cg03232621,cg04026763,cg22736383,cg00649211,cg12183221,cg13579926,cg21043328,cg19109130,cg12156301,cg00322636,cg14821382,cg19679907,cg24604179,cg15379240,cg22345349,cg07590638,cg18002794,cg04043623,cg21196575,cg16419334,cg24743791,cg27429663,cg03053581,cg16281582,cg21532323,cg04436431,cg03783948,cg10597337,cg08691275,cg23889393,cg05082920,cg17203119,cg25771226,cg25836827,cg21125848,cg25382025,cg19015686,cg07299078,cg12732791,cg05268347,cg20075927,cg24725441,cg14256726,cg04868072,cg07743179,cg25396464,cg05054831,cg06660522,cg20445518,cg10329699,cg25824209,cg02616069,cg10635686,cg20705908,cg21942150,cg25320496,cg09945822,cg01807303,cg07927482,cg20796544,cg01908874,cg22686253,cg09932730,cg20099906,cg02942186,cg04576317,cg03928133,cg18703875,cg03907995,cg17833937,cg14403762,cg17092609,cg16546489,cg16952929,cg26438672,cg16696019,cg16393125,cg24529493,cg15189797,cg16528891,cg22609459,cg10438671,cg15574362,cg12358298,cg22836066,cg03103952,cg04234556,cg26945780,cg08833787,cg14742920,cg19081751,cg14764876,cg00397989,cg15334572,cg15656470,cg15582335,cg08119847,cg12581354,cg14075156,cg12565693,cg04465185,cg01961025,cg02261637,cg17168358,cg07887939,cg03706883,cg04893543,cg26944546,cg10105974,cg21618529,cg26703520,cg25071880,cg10987801,cg09189978,cg22309080,cg25574190,cg04388075,cg06962592,cg24031228,cg14208859,cg24074432,cg06934003,cg25013571,cg13382117,cg14146491,cg04261102,cg11277260,cg22591672,cg13873902,cg10719247,cg00549234,cg07841686,cg23213158,cg02043219,cg07062938,cg02290978,cg16457259,cg18376227,cg15066992,cg09075968,cg04460747,cg01529454,cg08827274,cg17240701,cg21825944,cg13244886,cg06420058,cg19156941,cg17265790,cg17362299,cg24802374,cg14661969,cg02988795,cg06563713,cg23244169,cg20561213,cg10011978,cg04566233,cg04943797,cg04141261,cg19800728,cg08329428,cg08432712,cg25285383,cg09294492,cg08001899,cg09131903,cg23403117,cg02516530,cg21464596,cg24878059,cg03996123,cg27447083,cg10929866,cg15604158,cg04651240,cg10247061,cg19592720,cg02642298,cg07491206,cg03834906,cg08828531,cg18917736,cg24987706,cg05771997,cg21341391,cg14870682,cg20219894,cg01230154,cg06902235,

Intersections	No. of overlapping DMPs	List of overlapping DMPs
		cg00440702,cg06193995,cg15189860,cg02323296,cg04684390,cg06907056,cg12284736,cg05895956,cg12062067,cg25543190,cg00400085,cg09668276,cg14880184,cg14435997,cg05513661,cg09845205,cg26252027,cg21133608,cg00903154,cg25947600,cg21445204,cg18712148,cg07252892,cg01251118,cg04963523,cg03125787,cg13784878,cg14574871,cg18182059,cg04561294,cg21182103,cg06683481,cg25764839,cg03689082,cg02926797,cg09479793,cg27089079,cg06033859,cg24739950,cg21165395,cg12328023,cg18280859,cg13003329,cg27078893,cg14167748,cg16196745,cg18399457,cg26318674,cg24069815,cg07488365
		cg09463174,cg25272394,cg19002680,cg18373317,cg24606352,cg04558749,cg09150075,cg02385739,cg17685800,cg17911958,cg13103473,cg25997394,cg23673553,cg15519565,cg02246063,cg16734258,cg25364573,cg10460813,cg27437868,cg00228355,cg02022978,cg19188370,cg07146531,cg26626287,cg21818901,cg09067990,cg01732465,cg12977937,cg02577078,cg11102844,cg20609063,cg00844404,cg18761929,cg15633107,cg05122441,cg14178937,cg26242334,cg06923775,cg00395140,cg12629264,cg23519421,cg01654519,cg26139168,cg09113046,cg00382572,cg18222240,cg06578353,cg20192041,cg16201822,cg13324337,cg18234973,cg05456482,cg13864749,cg23101564,cg03949031,cg25230945,cg13855384,cg11835050,cg15592863,cg03174275,cg14422179,cg05716269,cg16340755,cg21069563,cg22001073,cg04950762,cg03387593,cg00756734,cg01600921,cg00364281,cg07799157,cg22722807,cg27047316,cg17307811,cg20672245,cg18135087,cg21216618,cg22802174,cg13415320,cg11003426,cg09766327,cg19596655,cg07427559,cg24445752,cg22227726,cg24690924,cg03218931,cg16638580,cg04612869,cg09153950,cg27360762,cg02967787,cg19593680,cg15157403,cg21868353,cg01065748,cg23066675,cg04766314,cg08032884,cg10458893,cg02737022,cg10539298,cg13202514,cg15012625,cg05620791,cg15791108,cg26746674,cg14010619,cg26543139,cg18548797,cg20712925,cg15014811,cg07649586,cg03859282,cg08089041,cg13648157,cg11718780,cg02352401,cg16412228,cg05227844,cg08996709,cg05745461,cg00177657,cg25369184,cg11648383,cg00401234,cg04948956,cg09821234,cg19631365,cg10258962,cg14623381,cg27261494,cg09785516,cg01682663,cg15278390,cg23284476,cg17218309,cg06611850,cg23999337,cg15691099,cg11741432,cg10396491,cg04310180,cg06728577,cg01405879,cg11333454,cg17964939,cg25962639,cg05445006,cg11340993,cg16462528,cg15219878,cg13387071,cg10024100,cg02289322,cg07627664,cg24940096,cg07722333,cg13242999,cg16068495,cg09817503,cg02537106,cg12327691,cg25190151,cg05580667,cg04345766,cg17854641,cg06131149,cg15082435,cg17219828,cg04993019,cg00551088,cg04019954,cg19689672,cg04377724,cg21367769,cg25492985,cg18101917,cg04299495,cg11734019,cg08898622,cg26504886,cg08515779,cg00281153,cg21330088,cg10701847,cg21877281,cg12579612,cg14618411,cg03664391,cg09540002,cg26831184,cg08938061,cg14699376,cg18116450,cg17983053,cg23095043,cg08376051,cg18947305,cg04006206,cg10518593,cg20073451,cg04331511,cg12633766,

Intersections	No. of overlapping DMPs	List of overlapping DMPs
		cg14246190,cg18761446,cg22730675,cg12343338,cg09571097,cg27416283, cg08377615,cg14559733,cg07940971,cg22792674,cg23996893,cg12767937, cg08571764,cg15052277,cg25431479,cg00795885,cg06837552,cg10658703, cg20803931,cg01025954,cg16696781,cg27400353,cg12809883,cg02552466, cg18499919,cg13476464,cg05507145,cg24674674,cg09610792,cg20381182, cg25204360,cg21600781,cg12400807,cg25992775,cg05908149,cg05533233, cg13201187,cg12603650,cg12668953,cg13386215,cg18016759,cg16501628, cg14829660,cg01158161,cg27317524,cg02039956,cg04720825,cg01406061, cg00026075,cg17491656,cg11444684,cg08593037,cg18645906,cg04403850, cg17209231,cg08338237,cg11252050,cg19478079,cg13151527,cg16413327, cg19782158,cg01764438,cg12874388,cg27370248,cg22502179,cg16181638, cg25083596,cg21350892,cg11505238,cg26728141,cg25739700,cg08705882, cg03047053,cg12049322,cg12351749,cg25116020,cg27530136,cg26242772, cg27073142,cg11137761,cg26089220,cg16255307,cg20693190,cg15565180, cg05511921,cg21203984,cg05919975,cg23874587,cg04173523,cg19822502, cg00339657,cg25372607,cg05575220,cg07005246,cg05875121,cg22082216, cg07336442,cg25319570,cg25501748,cg04577395,cg04751736,cg10624161, cg02571470,cg20140908,cg00373114,cg06412759,cg11724472,cg14644008, cg26174329,cg22979615,cg06089829,cg08862181,cg02885021,cg03808674, cg17391339,cg21243154,cg27000795,cg00077606,cg24379995,cg18118503, cg00779551,cg00943060,cg20010076,cg18494563,cg13060607,cg23707478, cg07246946,cg22156600,cg09860935,cg00309402,cg14014369,cg04100169, cg06798661,cg00042254,cg24817345,cg11553596,cg21170007,cg26646397, cg08700194,cg18193764,cg22380849,cg18565809,cg05835241,cg05083325, cg22677498,cg00965991,cg23225193,cg18414429,cg26855234,cg00354549, cg02849894,cg10015175,cg08931376,cg23267554,cg13754687,cg25940501, cg24493925,cg01937395,cg14929153,cg08657886,cg11140287,cg14011387, cg26725813,cg06265811,cg08904194,cg09322386,cg19865412,cg08275454, cg09788029,cg08224051,cg02921920,cg22241777,cg10096579,cg06828282, cg27180368,cg05360806,cg04653337,cg05365199,cg24980765,cg03885144, cg23184087,cg04181401,cg17585181,cg03701884,cg12367274,cg16306219, cg24064360,cg06069685,cg20158159,cg13231384,cg07127103,cg06819431, cg17426237,cg00204262,cg01305547,cg09232906,cg06245711,cg00091552, cg23611097,cg04975778,cg27259633,cg19970325,cg05024143,cg01711986, cg09456065,cg05897615,cg04478430,cg01555458,cg07831164,cg08276455, cg15297235,cg03061237,cg13705828,cg13462829,cg14299696,cg02709100, cg13443843,cg12101256,cg07780735,cg25793628,cg13951190,cg00019838, cg02635468,cg21594343,cg25646128,cg05962793,cg11114487,cg14445171, cg27352712,cg15569228,cg21274067,cg00130528,cg06380553,cg22573179, cg01399379,cg23212786,cg04819691,cg20505486,cg10706649,cg17825185, cg20107201,cg07146383,cg04943636,cg25519942,cg13412154,cg04556349, cg06279274,cg02798874,cg00077299,cg18450810,cg17167159,cg07679834, cg09526343,cg20153160,cg03463303,cg14528756,cg23096305,cg19716125, cg10337331,cg22222413,cg14239359,cg09795091,cg07568314,cg00039070,

Intersections	No. of overlapping DMPs	List of overlapping DMPs
		cg15447238,cg00432369,cg26321198,cg02025988,cg19166242,cg25450321,cg25326378,cg16991671,cg06991713,cg22040728,cg21545673,cg02095553,cg19419389,cg10946404,cg02741291,cg13382840,cg12380458,cg18599533,cg06829695,cg12048339,cg23967474,cg22925943,cg08124330,cg08458378,cg26946223,cg12523675,cg06957003,cg06695264,cg27213231,cg05777672,cg27548568,cg02814641,cg10994819,cg11635182,cg03627891,cg08575682,cg07792006,cg26053872,cg19773249,cg02256315,cg12225045,cg21111301,cg05302100,cg12770425,cg16773294,cg10356341,cg15464645,cg13781121,cg09690561,cg04140153,cg05895868,cg08248134,cg22350859,cg03216991,cg20489289,cg25443137,cg08111714,cg04941236,cg04965879,cg09012726,cg25114299,cg24935031,cg22009052,cg16500097,cg23793961,cg15328758,cg07675262,cg24078554,cg13937446,cg17418407,cg27646075,cg19502207,cg27133230,cg13435833,cg04947850,cg00414485,cg24601011,cg27501362,cg17843101,cg02113605,cg02698622,cg11882601,cg15874144,cg10832417,cg07967498,cg10090058,cg02548198,cg13754621,cg05445090,cg24933856,cg23933179,cg02671915,cg01774400,cg24523650,cg09655329,cg01006648,cg19548524,cg12649695,cg18935014,cg10256349,cg22622505,cg04942380,cg12478793,cg27638428,cg12798675,cg10795119,cg19425807,cg14292424,cg15550930,cg05949877,cg22591006,cg02210621,cg16117781,cg23574899,cg16984019,cg17731710,cg24630373,cg06069324,cg12837524,cg06910977,cg09382092,cg27407390,cg21949229,cg03653845,cg13800700,cg21610957,cg23279878,cg08106708,cg09344229,cg16098957,cg17365377,cg14594485,cg21538631,cg13361488,cg01200893,cg14188111,cg24808545,cg14942793,cg11658986,cg25340121,cg10135817,cg12977827,cg05040338,cg22883353,cg06471744,cg09581848,cg03128219,cg14841419,cg14543104,cg26437306,cg11322936,cg17146997,cg09770075,cg17518348,cg26208764,cg24664308,cg11503487,cg03938800,cg10493018,cg27530298,cg18987284,cg14563732,cg23144143,cg00770471,cg08700667,cg25450372,cg19938922,cg03687707,cg07442476,cg19476918,cg21648425,cg03325085,cg16105080,cg07193389,cg08244656,cg17006443,cg11022060,cg22540162,cg14015851,cg06055730,cg05819221,cg05013825,cg14736248,cg00459589,cg03994248,cg04767522,cg06590119,cg09232154,cg10308193,cg18414979,cg21196708,cg04329429,cg03059575,cg21908038,cg25866738,cg11973167,cg25505109,cg00589791,cg05295028,cg10984490,cg05224642,cg15288310,cg15189233,cg06582708,cg18834652,cg17367457,cg17136038,cg26423693,cg05241619,cg01877700,cg18579752,cg11153596,cg22968863,cg02266539,cg16396441,cg23685037,cg08497894,cg21544075,cg03718620,cg17510601,cg11553667,cg24165107,cg10173749,cg04180473,cg14921275,cg15172011,cg17548932,cg09081517,cg04509238,cg03646297,cg15055062,cg15381433,cg03980035,cg18365806,cg24923365,cg19071460,cg10167756,cg05655647,cg06795980,cg22021768,cg18054654,cg17158584,cg15107887,cg22076764,cg15354877,cg02955100,cg25077271,cg08289189,cg06100373,cg16596250,cg02936043,cg27394814,cg26832154,cg17578638,cg00775777,cg22877331,cg14373718,cg12301921,cg25567280,cg06460350,cg10992704,cg05945844,cg00846218,cg06455125,

Intersections	No. of overlapping DMPs	List of overlapping DMPs
		cg20947553,cg11069461,cg01172235,cg20149922,cg05694825,cg08822635,cg10351418,cg04773376,cg02789915,cg26461449,cg04707327,cg17429234,cg12209160,cg15358035,cg02754643,cg20150189,cg20194814,cg11753004,cg11282100,cg15167836,cg01100030,cg27098587,cg17299451,cg03510444,cg20428196,cg25824592,cg01691522,cg06466360,cg27241677,cg10682833,cg04974940,cg27000687,cg02370040,cg12057368,cg00554165,cg26786687,cg01882222,cg22752345,cg27129076,cg27623867,cg05696056,cg09783493,cg25961221,cg04221836,cg07609108,cg09851981,cg27332018,cg13263013,cg07159131,cg03455828,cg13997640,cg24212302,cg22889322,cg11395890,cg07757545,cg22937571,cg22849967,cg04770593,cg11501364,cg17900273,cg02433406,cg04237127,cg12710220,cg07510510,cg01813672,cg07887012,cg12536921,cg17722656,cg17132591,cg05271080,cg02664328,cg06084702,cg05636830,cg13079633,cg05207611,cg06073550,cg04591308,cg05040778,cg10958840,cg15849831,cg04770282,cg18452155,cg05610699,cg11429177,cg08138407,cg01238140,cg06967016,cg14311690,cg07359806,cg17310354,cg15554631,cg06255741,cg12453585,cg23978557,cg03204787,cg24797931,cg04338834,cg11523381,cg04696881,cg09850161,cg23492413,cg17035490,cg21673010,cg01868128,cg05572584,cg05625839,cg02071267,cg24327491,cg13917023,cg20090429,cg21840672,cg25342481,cg14461273,cg26965819,cg06942743,cg10591077,cg19841740,cg26154888,cg04995753,cg14995181,cg24516912,cg14292220,cg07505515,cg14071249,cg00836097,cg07482451,cg24576285,cg04993041,cg15310513,cg02080152,cg24349106,cg08485389,cg09106587,cg07158881,cg15910230,cg03906073,cg04660984,cg23158658,cg18082885,cg03630756,cg07476353,cg00469797,cg04036088,cg06575847,cg25924827,cg10809872,cg12602291,cg06889183,cg17858098,cg13089369,cg02198582,cg19847588,cg16343302,cg15849550,cg03611573,cg25386688,cg22053565,cg14288953,cg01071153,cg01551350,cg07785225,cg27312255,cg01966599,cg22867154,cg03421069,cg08960663,cg06098801,cg27303125,cg11739512,cg05920666,cg16292912,cg16684846,cg15937191,cg19628908,cg19656808,cg19949797,cg05207255,cg17860201,cg06381000,cg13107582,cg03496777,cg11046579,cg03971661,cg17173442,cg17727418,cg26675620,cg13534903,cg10138313,cg19812747,cg23547656,cg21158975,cg12263794,cg20316715,cg13355312,cg08073874,cg19584834,cg27082426,cg08169026,cg16035667,cg10120616,cg12163415,cg03374148,cg19942560,cg23373897,cg07055311,cg08132525,cg23695211,cg27413230,cg16923710,cg04460007,cg04414629,cg25623815,cg08547352,cg25596655,cg13655908,cg01444149,cg20979307,cg05982597,cg24970360,cg27385049,cg12631085,cg05786698,cg12819781,cg11849302,cg18948941,cg22656997,cg19996313,cg12581446,cg07829209,cg13173369,cg12033909,cg15323066,cg14281264,cg03378998,cg27075492,cg01236774,cg00389785,cg26007766,cg19497709,cg27518314,cg05389374,cg01055075,cg12495591,cg02046941,cg02362718,cg17868687,cg20789811,cg06543365,cg17162808,cg20250700,cg16447054,cg24730688,cg17311802,cg02753990,cg08505135,cg19695166,cg21664781,cg25639848,cg23851043,cg11191979,cg08154700,cg06831389,cg22984417,cg20610838,

Intersections	No. of overlapping DMPs	List of overlapping DMPs
		cg16910047,cg06550991,cg20731623,cg03578552,cg00841768,cg02542806,cg10433438,cg20460709,cg22029774,cg24027594,cg24512770,cg08441414,cg16311685,cg10819807,cg02485558,cg10616902,cg17917920,cg09675993,cg03031322,cg13487037,cg01235795,cg21072513,cg03673295,cg03263979,cg13344470,cg10333051,cg02791765,cg16775198,cg04982874,cg03179726,cg15177410,cg03242401,cg16618979,cg19628012,cg26611367,cg07369260,cg19610848,cg20797552,cg26870594,cg19955257,cg09582410,cg20689224,cg04792642,cg22549383,cg25744558,cg06952534,cg09806966,cg22139983,cg15236453,cg17794241,cg13990958,cg08506445,cg24131397,cg26241746,cg17944001,cg18695750,cg19816059,cg00864563,cg11553654,cg23303070,cg08458655,cg04547433,cg08161136,cg03195665,cg00593414,cg03185283,cg11658588,cg25418870,cg27548735,cg24994648,cg13359037,cg10911328,cg17002428,cg04197935,cg25533594,cg07035001,cg08152567,cg05233550,cg22636056,cg23842331,cg24499677,cg15045771,cg08869857,cg08676001,cg18412326,cg20357807,cg25306357,cg22129059,cg03731358,cg18971492,cg07221511,cg01125327,cg02058944,cg12858231,cg21253881,cg25312546,cg18291151,cg27308967,cg12941675,cg04174710,cg01405685,cg17881380,cg07242196,cg08814020,cg22335802,cg18474718,cg14179383,cg13291666,cg03319082,cg23636571,cg27192892,cg02898904,cg17932974,cg05653753,cg06453853,cg25926652,cg26155739,cg11928962,cg13551229,cg10011963,cg19555833,cg18286995,cg26322995,cg19664425,cg14438634,cg04881008,cg00776252,cg07799639,cg19086488,cg09144905,cg08996975,cg15844527,cg24958423,cg06311795,cg04830191,cg04759112,cg23985930,cg08795994,cg23416909,cg24509237,cg05790237,cg13757172,cg15442567,cg03461361,cg21963218,cg04521279,cg04917208,cg01206398,cg21682210,cg20802214,cg19656799,cg18281090,cg22205607,cg10245488,cg21670828,cg23935361,cg13807341,cg05858115,cg00054572,cg00395929,cg13482378,cg03493513,cg26890309,cg06082228,cg12309550,cg18155344,cg01641468,cg18013249,cg06712763,cg11152223,cg11156733,cg14558153,cg06816421,cg03361504,cg12280796,cg23481184,cg02563407,cg26569349,cg23826579,cg20602914,cg05285768,cg03119599,cg22416672,cg18411194,cg24524735,cg14522790,cg24466100,cg02621779,cg27467249,cg06659282,cg06538888,cg18755544,cg26299763,cg19147499,cg20383654,cg24434232,cg16778451,cg02091607,cg09854814,cg10817408,cg22075974,cg12884510,cg17001032,cg23538344,cg00114618,cg21898708,cg11243251,cg18826743,cg14929317,cg26131114,cg22826803,cg22963191,cg14433609,cg04260057,cg18530840,cg09808196,cg13167465,cg22149095,cg00263314,cg04258934,cg27379587,cg14170835,cg16453673,cg24916055,cg18345539,cg19445642,cg03535648,cg14545914,cg22732549,cg05063097,cg03189593,cg15085842,cg12286804,cg13703095,cg21425723,cg09388113,cg12139156,cg07518353,cg23151747,cg19165344,cg23812013,cg02173969,cg07010337,cg04202122,cg07763398,cg08363518,cg20502257,cg24819427,cg17978425,cg25214823,cg25492458,cg21619881,cg02643580,cg24807354,cg09552983,cg25582105,cg18946932,cg16553051,cg06364539,cg02007235,cg06407259,cg04317573,cg26365152,cg26511326,

Intersections	No. of overlapping DMPs	List of overlapping DMPs
		cg21212977,cg01776221,cg23833195,cg04383038,cg07360201,cg02399275, cg01438537,cg17643729,cg17781357,cg20251199,cg25400753,cg07814816, cg14317321,cg24766109,cg07378405,cg08302468,cg15025569,cg02634745, cg12482983,cg09601319,cg25106036,cg08079970,cg21006422,cg07613952, cg17767957,cg04427512,cg16292030,cg20708199,cg13331741,cg05843946, cg12075776,cg07733129,cg18918365,cg22406758,cg21214969,cg22269416, cg08282043,cg01554257,cg14917136,cg02786257,cg23117565,cg08262598, cg23794200,cg17906245,cg26131292,cg27541349,cg09597666,cg16166262, cg02083151,cg27610521,cg21295398,cg01446270,cg14529610,cg23432249, cg25742745,cg18606993,cg22907589,cg25265769,cg18582979,cg03972219, cg14642381,cg24027542,cg07029201,cg24996280,cg22241014,cg10103235, cg06336578,cg04772797,cg00711386,cg07962218,cg08804555,cg16103996, cg06681324,cg25477839,cg11389953,cg05739305,cg14575222,cg09800974, cg06662976,cg23876355,cg17207090,cg07589034,cg08842388,cg24938659, cg26134629,cg18048655,cg00422716,cg03798162,cg00674003,cg04415672, cg24436207,cg23911661,cg07252933,cg10595421,cg02278087,cg19765038, cg00336555,cg21504005,cg15624335,cg15789385,cg00026346,cg19733514, cg26240939,cg09373254,cg11062167,cg16003857,cg06485995,cg04362315, cg19875936,cg05139219,cg02605708,cg03820779,cg19887341,cg23528492, cg00200667,cg21048411,cg13728650,cg20960953,cg27054055,cg08773876, cg13582500,cg02864626,cg12399860,cg12749280,cg27233071,cg13557160, cg13051865,cg15612847,cg03039137,cg07021678,cg22853813,cg01952473, cg20604957,cg12768447,cg02397061,cg09554099,cg13300509,cg00491469, cg26682641,cg23521138,cg04904531,cg20182709,cg18395233,cg26894484, cg26497076,cg11985745,cg25463779,cg20672131,cg22152082,cg17447300, cg01665970,cg21192260,cg00877619,cg04989533,cg08439328,cg15646967, cg07499201,cg01729288,cg20919914,cg09407223,cg24576666,cg10166160, cg01503773,cg26074575,cg09314196,cg03214637,cg07752079,cg07437235, cg06407709,cg11159299,cg08284985,cg04011354,cg07437737,cg05837491, cg22009239,cg02926601,cg03680769,cg08786064,cg12683742,cg08082943, cg26587014,cg00593446,cg14223709,cg03694517,cg15541890,cg26212701, cg26974427,cg27271242,cg10023469,cg16868500,cg18241486,cg11920576, cg06552046,cg19507893,cg16949584,cg00244317,cg08815352,cg14076860, cg03805782,cg04311699,cg16760971,cg19811786,cg09032303,cg01770076, cg15830449,cg09974839,cg00591225,cg24140200,cg11132411,cg10936233, cg00201595,cg21106661,cg24849108,cg13449831,cg04884171,cg15988897, cg26232655,cg17676119,cg27085560,cg07743747,cg25423732,cg11100216, cg27471183,cg27657326,cg19033271,cg15747531,cg10736955,cg17056317, cg13986498,cg13357330,cg12470218,cg07757454,cg07426622,cg13788387, cg22892485,cg24291991,cg00137745,cg00373376,cg25144109,cg19541260, cg10131227,cg10510779,cg22298470,cg16215362,cg04072337,cg19721541, cg02024219,cg21664212,cg26832183,cg14252850,cg06651838,cg07637658, cg16875108,cg14694085,cg22843395,cg21812670,cg13507091,cg19732625, cg16733589,cg07498239,cg24544275,cg17526060,cg16562054,cg11798814,

Intersections	No. of overlapping DMPs	List of overlapping DMPs
		cg01624081,cg15712821,cg27113736,cg25673945,cg05251479,cg26808852,cg19751869,cg23101686,cg21752889,cg10715333,cg00661205,cg13436725,cg17661103,cg08997913,cg06995299,cg10124011,cg07512790,cg21433538,cg05336067,cg00795247,cg00771653,cg09614350,cg02955037,cg05381183,cg14505667,cg03014969,cg01406909,cg11002558,cg05424526,cg03760158,cg19366381,cg09463155,cg04167811,cg02629093,cg22443651,cg03987813,cg16486754,cg16494108,cg02833379,cg24014141,cg04981665,cg18380211,cg04280377,cg18098053,cg00993710,cg11508081,cg26011210,cg14112555,cg05259332,cg19629216,cg06087932,cg01374654,cg05882673,cg18920489,cg00507855,cg23199403,cg17046348,cg06803290,cg12807855,cg22017654,cg07669814,cg07724487,cg26479971,cg23035238,cg06308323,cg24094412,cg17340810,cg25542337,cg11623932,cg24033957,cg01124868,cg01013685,cg24162579,cg05739419,cg17686973,cg16615900,cg14106242,cg01492146,cg21077030,cg01843766,cg04244183,cg06868413,cg00090612,cg14089603,cg24003649,cg02512224,cg08107352,cg03778373,cg08499057,cg21564514,cg23190402,cg14048785,cg08629097,cg04252529,cg22328929,cg24813180,cg03984344,cg03398966,cg15844647,cg25139877,cg04030146,cg25020232,cg24157008,cg24516399,cg02838309,cg14701925,cg22395811,cg19370278,cg12163646,cg15718106,cg12543807,cg08864829,cg23835338,cg06034437,cg01379680,cg11689744,cg06620605,cg22166248,cg19405941,cg12039660,cg13663667,cg14615959,cg21931174,cg21343859,cg24684576,cg04648500,cg11949533,cg14755054,cg04791421,cg10828900,cg15683356,cg03490448,cg09307250,cg27380412,cg13560204,cg03833993,cg21782357,cg23516537,cg18143116,cg23919678,cg07703679,cg11555339,cg19863964,cg08998414,cg25593022,cg06785359,cg02245418,cg19440553,cg07441476,cg01938295,cg02668655,cg09409500,cg20568659,cg07611351,cg14239588,cg19500057,cg25882056,cg08609477,cg07990036,cg21186771,cg05568311,cg25743531,cg08565320,cg06882504,cg03446694,cg19397969,cg15058852,cg05476521,cg22104394,cg04057161,cg11065418,cg10768614,cg05451078,cg27338653,cg08739385,cg00360840,cg04114537,cg14222000,cg13491023,cg06543160,cg01180835,cg20768515,cg02432263,cg27604200,cg00694660,cg24834165,cg17165818,cg14390764,cg07989373,cg14330517,cg03805357,cg06658821,cg10163580,cg17651095,cg17823376,cg20027331,cg19786648,cg13973939,cg07573797,cg24152718,cg03155233,cg10456628,cg19297164,cg24663131,cg14189381,cg16709217,cg21950208,cg20662420,cg18565899,cg18140324,cg22216431,cg01693326,cg24688425,cg14832223,cg11859176,cg20328379,cg08464855,cg25094834,cg14233513,cg04860622,cg09510269,cg04847386,cg07856421,cg16673286,cg09759808,cg11813940,cg19910022,cg13482911,cg05231098,cg08919528,cg26124514,cg24581736,cg24755266,cg08826384,cg00590383,cg12098896,cg13617603,cg27500542,cg18599231,cg01521143,cg09274104,cg10340872,cg07708653,cg17182202,cg19851416,cg18412394,cg16527939,cg05198670,cg01324000,cg16017492,cg01590359,cg07476792,cg04581073,cg02114341,cg16500769,cg03953086,cg01450736,cg04850842,cg03077730,cg15066656,cg00502963,cg18367027,cg04677581,cg04240603,

Intersections	No. of overlapping DMPs	List of overlapping DMPs
		cg16184346,cg18877514,cg02042467,cg00447557,cg16067453,cg24974704,cg13628100,cg05117124,cg26191710,cg13965201,cg15893396,cg18906478,cg27043531,cg21005240,cg11512797,cg15861059,cg25401219,cg06377069,cg03500611,cg19639071,cg02496113,cg05781820,cg15764655,cg18718349,cg15703165,cg05407135,cg00927227,cg08441455,cg01977520,cg10891008,cg12581891,cg22795059,cg20386784,cg04342863,cg16669468,cg09123455,cg22797978,cg13553897,cg15615365,cg13665979,cg10800519,cg00754836,cg04074650,cg13489424,cg26554494,cg27422721,cg03269320,cg14805174,cg04260065,cg24446166,cg09200760,cg09303484,cg18331135,cg22533480,cg27168606,cg01025185,cg18190417,cg23550034,cg00415939,cg16400196,cg08977027,cg10683565,cg07327335,cg06879534,cg21279353,cg08488494,cg21140633,cg18623351,cg16789902,cg18601334,cg15597147,cg15076179,cg20713712,cg12549595,cg18454218,cg17440757,cg00081046,cg08550689,cg03318222,cg21126306,cg11648402,cg01855601,cg19852607,cg21447989,cg10531521,cg22992730,cg17240885,cg02193513,cg11402099,cg18646208,cg17260058,cg18172802,cg03966669,cg05935660,cg14573897,cg06504624,cg01799186,cg05222807,cg21430959,cg16336227,cg04605316,cg06761437,cg14013161,cg08882503,cg12743556,cg15572423,cg22529427,cg10725048,cg20494682,cg14243616,cg22495211,cg02817770,cg04191142,cg19791907,cg26735886,cg23726945,cg23961141,cg03319070,cg02725493,cg14232165,cg05041475,cg04749197,cg01091239,cg05519080,cg24250234,cg25921194,cg17467517,cg25046866,cg09173378,cg13660596,cg27462497,cg18188326,cg10778931,cg10430021,cg03615063,cg14597822,cg01048203,cg11615495,cg15842559,cg24855045,cg08163621,cg13726504,cg21645075,cg21610554,cg06650698,cg08522655,cg27180636,cg26588398,cg14939641,cg18646671,cg13569714,cg21460946,cg03293095,cg01681290,cg19254426,cg24158437,cg05616608,cg16027728,cg26654766,cg17886662,cg10646179,cg17521526,cg16311808,cg13189097,cg17442548,cg26309261,cg09791009,cg03511766,cg10003974,cg02574374,cg11675271,cg20458807,cg15932610,cg24320065,cg26424817,cg12103802,cg06309053,cg08715988,cg27105890,cg04319742,cg06293195,cg05873359,cg02964602,cg20919336,cg12845900,cg10414477,cg23686321,cg23994714,cg01026613,cg01488306,cg06944489,cg12866769,cg09131512,cg03665229,cg19771031,cg03525847,cg10798225,cg19648192,cg19046642,cg02874396,cg15443166,cg09548799,cg03840912,cg19677184,cg26609894,cg23839398,cg01063509,cg23308914,cg13717555,cg20029324,cg05745622,cg26496604,cg10210086
		cg00010242,cg04917688,cg12210527,cg02255104,cg25764935,cg15815117,cg07729230,cg06874985,cg03880785,cg19661819,cg01853974,cg03983715,cg19690486,cg18358845,cg09882550,cg12882999,cg26679144,cg18285636,cg04482148,cg24209994,cg14000815,cg02348347,cg16114141,cg02050613,cg04906603,cg01976076,cg03643116,cg11678206,cg06733294,cg09570676,cg03987573,cg13924868,cg14218108,cg25456633,cg25616771,cg25413125,cg12336935,cg26492631,cg24693463,cg07784294,cg17228560,cg02774855,

Intersections	No. of overlapping DMPs	List of overlapping DMPs
		cg22754489,cg24302646,cg09838857,cg20693615,cg03482617,cg14792002,cg09383106,cg24841310,cg13247922,cg09279458,cg16715662,cg02289590,cg10595449,cg03530962,cg13570861,cg02600413,cg23089436,cg06719602,cg03656632,cg01152927,cg18849025,cg20284982,cg09470963,cg23702117,cg07904879,cg04409981,cg17206564,cg03670422,cg21844726,cg18903373,cg05372957,cg04719454,cg15708175,cg07186585,cg20374041,cg11064395,cg22993715,cg03177729,cg14847658,cg23363484,cg17565410,cg15612221,cg08780974,cg05846057,cg08491363,cg09711421,cg08577693,cg02693118,cg22389215,cg07902406,cg19819089,cg06044794,cg02222496,cg08047889,cg04315682,cg10318313,cg26389381,cg20177086,cg18731202,cg18528216,cg07144026,cg23392359,cg12989745,cg20187320,cg19405799,cg07395611,cg07811719,cg19733740,cg20164279,cg23318676,cg04098023,cg26836171,cg13956645,cg23256971,cg11607882,cg16023306,cg23919830,cg03657837,cg02519625,cg01837661,cg00214970,cg13073030,cg11780096,cg26579073,cg20516364,cg18706881,cg04722593,cg02862516,cg10565537,cg12496800,cg24908403,cg10709331,cg15162320,cg04606471,cg01858583,cg20823181,cg26620724,cg09436495,cg11953399,cg02678160,cg23920445,cg14650677,cg27599319,cg27350579,cg25905578,cg00402551,cg06592890,cg01695994,cg27089219,cg13128596,cg27365621,cg02422011,cg06374204,cg08160317,cg13346967,cg11023992,cg13010190,cg27002271,cg26389958,cg15367311,cg02854763,cg06031526,cg20330521,cg10762533,cg23146699,cg13932046,cg00712510,cg20677261,cg00098162,cg20414753,cg25195309,cg19930290,cg16707391,cg13777488,cg04267164,cg02348876,cg08409566,cg00275090,cg17799654,cg22020455,cg20776543,cg19929302,cg09474173,cg20621459,cg03482534,cg19405371,cg07923588,cg03916783,cg19078226,cg14559160,cg26604287,cg22553555,cg09315465,cg13571852,cg10617091,cg18826070,cg27528695,cg27233997,cg18581500,cg16790423,cg23622026,cg22643259,cg17170868,cg23191740,cg00046410,cg10537848,cg09408023,cg23331837,cg25355465,cg20948377,cg27349129,cg21583727,cg12072724,cg04247746,cg23448432,cg12106346,cg14195606,cg04080291,cg17942553,cg01553616,cg21536042,cg11860760,cg13450407,cg10775350,cg20994248,cg25780806,cg27570978,cg08173660,cg05305060,cg04849089,cg09938469,cg19874421,cg15177169,cg17956259,cg23460070,cg27061477,cg19723678,cg07633119,cg02362984,cg02905251,cg00351097,cg00150128,cg17342360,cg11068217,cg22621535,cg05050047,cg11085062,cg17299799,cg06739505,cg03132727,cg06379858,cg10210919,cg07332388,cg18224798,cg15574240,cg11308338,cg00905858,cg05581297,cg09190827,cg02167717,cg00252057,cg09240555,cg02773045,cg03237134,cg22058467,cg11834694,cg02158100,cg03898302,cg23435916,cg11911454,cg08805661,cg25783153,cg04127706,cg26249038,cg13947137,cg09829842,cg05555396,cg06596613,cg00948878,cg00388439,cg01342196,cg16766036,cg14081995,cg18531720,cg23044169,cg26970316,cg01936488,cg15684015,cg01845476,cg27147706,cg23413954,cg27314482,cg22005656,cg04546061,cg21358429,cg08369061,cg18234280,cg07681834,cg15934100,cg01206872,cg22525154,cg14299539,cg26532102,cg01292810,

Intersections	No. of overlapping DMPs	List of overlapping DMPs
		cg07003739,cg19061089,cg25579656,cg26240842,cg00202819,cg02527496, cg16196762,cg03644750,cg01058212,cg26124136,cg14333935,cg22275321, cg01276327,cg19453726,cg19127835,cg05000860,cg18545561,cg14878302, cg22847617,cg18767287,cg27322975,cg08988609,cg09734033,cg02642031, cg18443741,cg23951861,cg07075118,cg02165614,cg00539325,cg12805450, cg20151125,cg01566282,cg27222368,cg23413074,cg14962447,cg02771587, cg17604181,cg09866659,cg14737320,cg18444254,cg11546385,cg13428239, cg14202087,cg08965262,cg04707217,cg03077969,cg18683453,cg04502857, cg06438301,cg13896861,cg09744732,cg11319211,cg07876320,cg15999636, cg26010028,cg21821108,cg11957884,cg15195097,cg00906838,cg19497939, cg23936681,cg21211664,cg13518537,cg05343106,cg16437896,cg18596139, cg10448111,cg03394122,cg02807271,cg07855322,cg12798311,cg22130868, cg04636881,cg23270103,cg14216348,cg04321793,cg03747030,cg13451471, cg15077416,cg01834373,cg23797052,cg25939203,cg10438121,cg12193365, cg27016609,cg16230999,cg01390728,cg15939182,cg20273485,cg05677184, cg20150443,cg00301902,cg23557788,cg03793551,cg08598966,cg21443763, cg07107196,cg18792689,cg13267821,cg23154064,cg27046079,cg17463277, cg16047994,cg25212814,cg08171276,cg22375720,cg08400246,cg10675515, cg09946623,cg20788083,cg08160871,cg18569952,cg13295812,cg09095792, cg07672065,cg01675895,cg22339219,cg02418072,cg13827173,cg10410904, cg06530991,cg01048972,cg25672097,cg00288957,cg16522960,cg20075582, cg07402303,cg18345844,cg21857146,cg25990920,cg05921316,cg01522278, cg25714060,cg19549314,cg13504480,cg00897276,cg06127619,cg00410323, cg12298241,cg00065368,cg12738078,cg02569596,cg06401138,cg08982952, cg02189283,cg14946950,cg05828992,cg12225467,cg03313815,cg01448931, cg20276402,cg13731703,cg24505274,cg00498419,cg14266348,cg24676969, cg25217895,cg16277405,cg15807288,cg22986077,cg13559773,cg23978473, cg21527556,cg13925220,cg16095772,cg00559439,cg12007683,cg06853577, cg19263575,cg23480284,cg18546110,cg02333281,cg25039993,cg10543219, cg02646611,cg24374538,cg03541365,cg03117577,cg01548199,cg03751734, cg02898019,cg16320398,cg17092860,cg22232708,cg06183295,cg20029156, cg17242590,cg12318152,cg21690675,cg11911128,cg15263875,cg09573809, cg13444243,cg10503283,cg10681811,cg26309498,cg21005459,cg23021237, cg25837984,cg23811122,cg01516809,cg12786765,cg17752707,cg02276269, cg10795552,cg25172808,cg20173120,cg24048209,cg22068759,cg09248887, cg00395420,cg10537017,cg16503713,cg14431577,cg00458927,cg03484703, cg14600600,cg03250393,cg25370836,cg07465899,cg22869283,cg22626747, cg05302542,cg02887598,cg16026862,cg26795179,cg03194151,cg13589840, cg02696351,cg21538860,cg22207479,cg16909293,cg00094333,cg11193840, cg07372609,cg15033776,cg18205372,cg10784548,cg23691733,cg13429967, cg27437526,cg02953432,cg11646018,cg16556679,cg24715709,cg15624109, cg23430209,cg25605360,cg13978409,cg23236301,cg20872422,cg07839513, cg02764852,cg10080250,cg26758386,cg27116232,cg27042091,cg02921604, cg01128794,cg10947827,cg11940381,cg08096684,cg08846312,cg07911444,

Intersections	No. of overlapping DMPs	List of overlapping DMPs
		cg05774001,cg09439010,cg00087898,cg20677521,cg06905516,cg17266153,<br>cg25826644,cg25314134,cg03359428,cg19527748,cg05093315,cg04152341,<br>cg22717900,cg07740914,cg13445177,cg01182660,cg02703695,cg05947505,<br>cg09403024,cg20643159,cg09003326,cg06722917,cg20615655,cg23088110,<br>cg16937266,cg05105110,cg19785312,cg24651790,cg02105843,cg00752077,<br>cg13821031,cg08504577,cg09321430,cg14077232,cg26218805,cg13998226,<br>cg27307421,cg26984388,cg05881156,cg04284956,cg24710191,cg05222072,<br>cg22044134,cg04136817,cg08586494,cg02701137,cg00082656,cg08183300,<br>cg20706620,cg06659614,cg22068629,cg26140512,cg19502179,cg05263743,<br>cg21565802,cg15435485,cg03492460,cg22386757,cg00955895,cg23694533,<br>cg22445346,cg00342613,cg16438069,cg24716494,cg03342862,cg24500304,<br>cg04603928,cg08071391,cg14385566,cg24009362,cg12431453,cg14679235,<br>cg03169843,cg27524003,cg17748504,cg26836125,cg16010427,cg05546296,<br>cg09532502,cg06057750,cg02491003,cg11908291,cg19732831,cg08822578,<br>cg17155610,cg22153062,cg03859106,cg24108888,cg02084403,cg18885299,<br>cg09577966,cg01313965,cg10402804,cg26771320,cg13440882,cg04258046,<br>cg26860848,cg17027033,cg02755555,cg12142501,cg13673363,cg20454179,<br>cg00821510,cg04879348,cg21046959,cg06834705,cg00984787,cg27464378,<br>cg11896012,cg22392809,cg02159654,cg12484332,cg17578309,cg10215079,<br>cg03360716,cg00446213,cg05110629,cg00491591,cg13582724,cg02132460,<br>cg11807290,cg11677597,cg21185154,cg15749141,cg13810964,cg08249467,<br>cg01946966,cg21738716,cg04144604,cg07258419,cg19463703,cg08972843,<br>cg07783291,cg03281705,cg02275213,cg26840062,cg23341970,cg18007641,<br>cg16376484,cg16710828,cg21071940,cg19302283,cg00345656,cg05157272,<br>cg03982998,cg14129714,cg12589936,cg07190630,cg13831141,cg10533493,<br>cg02786368,cg09271353,cg08042477,cg00977895,cg02015439,cg18027632,<br>cg24331317,cg13950162,cg04458920,cg02812757,cg20497713,cg07573786,<br>cg26889774,cg15929624,cg20731100,cg03545117,cg20938103,cg24761212,<br>cg01064432,cg21751779,cg10110998,cg06297155,cg24839562,cg01630829,<br>cg09069150,cg14638662,cg18881992,cg05218782,cg06160221,cg01203563,<br>cg18690712,cg20786740,cg10010004,cg09529065,cg23455982,cg06655570,<br>cg05742691,cg11491652,cg02981094,cg22280402,cg08885316,cg16192293,<br>cg07520673,cg15412313,cg24073060,cg16741463,cg00147332,cg07596401,<br>cg00846287,cg02263880,cg17935214,cg09474625,cg03713453,cg15179921,<br>cg19008523,cg26469821,cg01840724,cg26917697,cg15992843,cg02913804,<br>cg21397124,cg00554341,cg06708683,cg14400821,cg00226961,cg19884600,<br>cg02295872,cg05632319,cg12266722,cg20625065,cg02354916,cg03716582,<br>cg03683004,cg12048805,cg13413387,cg21211551,cg05829424,cg08485259,<br>cg12257055,cg24838661,cg22882684,cg10955633,cg14509631,cg06157732,<br>cg15825373,cg01615453,cg19682249,cg20338276,cg26884837,cg02183561,<br>cg23678918,cg07875692,cg02002125,cg19892758,cg15482213,cg26950876,<br>cg17696660,cg02869126,cg04442042,cg17296165,cg21282998,cg06212582,<br>cg16758348,cg03552317,cg11072045,cg14161995,cg07629796,cg01195526,<br>cg01049121,cg24552391,cg11592938,cg03656734,cg00183569,cg10023624,

Intersections	No. of overlapping DMPs	List of overlapping DMPs
		cg03385207,cg01237853,cg25615890,cg20262540,cg06240859,cg10368507,cg10910666,cg14071459,cg24996200,cg26789922,cg00439735,cg03446203,cg26475819,cg00451058,cg12555369,cg18802268,cg04347820,cg09588434,cg25080322,cg11067338,cg13388830,cg27087192,cg23836113,cg21608387,cg01954649,cg20894569,cg21792806,cg19755756,cg10059104,cg05274503,cg10861426,cg08930897,cg25273903,cg03659851,cg22674789,cg19639021,cg18160462,cg06112082,cg02923302,cg06600200,cg21191508,cg21164545,cg01713095,cg26441097,cg22449686,cg12404462,cg09314700,cg06531517,cg10854092,cg24313161,cg23907004,cg20895054,cg08924911,cg03272958,cg20915460,cg11030124,cg15402112,cg07801506,cg21513537,cg09013092,cg16600869,cg06910896,cg05266321,cg11754807,cg13188416,cg02230692,cg02130289,cg05877590,cg19920161,cg13573346,cg15227771,cg01486987,cg00546808,cg22077962,cg23630878,cg10890917,cg02241545,cg09366887,cg24462263,cg14283475,cg11973514,cg20590059,cg15757342,cg08594632,cg19458893,cg17341118,cg01630362,cg06868034,cg12320621,cg17841099,cg07970724,cg00764840,cg13932217,cg11265874,cg22621663,cg26108836,cg04591623,cg03141971,cg01449444,cg17792882,cg27413928,cg08995473,cg26852404,cg12430902,cg24461508,cg20145695,cg02953679,cg18795277,cg18726360,cg05571215,cg26085762,cg16511392,cg18436714,cg16886882,cg13552506,cg08028698,cg03824238,cg22981198,cg07921682,cg20140735,cg20986076,cg16657691,cg27620524,cg03353474,cg05333174,cg19535004,cg10867456,cg01398168,cg27595997,cg10959344,cg05836646,cg09566453,cg05934333,cg26301626,cg13122532,cg21238276,cg24859593,cg15996647,cg27119063,cg12789833,cg10578681,cg22270690,cg21148531,cg14259893,cg01703196,cg18522822,cg22692213,cg26317888,cg18070504,cg00413370,cg26743898,cg09201173,cg10256790,cg23882417,cg23849965,cg08068547,cg17947225,cg11163433,cg26174797,cg17324872,cg14244739,cg17628468,cg12334842,cg00076936,cg14997702,cg21125095,cg04065297,cg00051510,cg23990682,cg11019127,cg12608975,cg17730759,cg16058430,cg21385748,cg06921605,cg20119308,cg14243899,cg21831927,cg12482432,cg27551697,cg09853648,cg10954454,cg22669624,cg08719077,cg14727763,cg09450550,cg13685779,cg11806823,cg24704082,cg03983684,cg00506859,cg00365411,cg26713125,cg02663317,cg06360121,cg15057735,cg12750276,cg02035015,cg07980295,cg04848972,cg07575166,cg26283801,cg26691898,cg15531814,cg00175423,cg17419329,cg15266307,cg07943688,cg14683055,cg22633597,cg09751673,cg20268994,cg16966520,cg10241247,cg16673712,cg14038214,cg18689082,cg22272240,cg15626873,cg22562498,cg14330621,cg24060317,cg17528325,cg10967866,cg14212631,cg03890998,cg03974286,cg12098665,cg15598425,cg09770389,cg19683188,cg23262420,cg19248505,cg25245744,cg21152173,cg25576282,cg09813341,cg13050142,cg10996532,cg16741245,cg16873780,cg05344692,cg05583095,cg04884395,cg18461093,cg04140267,cg09168272,cg13554030,cg06633484,cg10643608,cg26637826,cg00715913,cg16091630,cg01447350,cg27638509,cg10314470,cg27411706,cg19316897,cg15152789,cg01795660,cg13483593,cg00062070,cg02481294,cg08866469,

Intersections	No. of overlapping DMPs	List of overlapping DMPs
		cg21694626,cg15652280,cg00178785,cg01009068,cg00727586,cg13960894, cg17672031,cg25367374,cg26063030,cg20247222,cg14036226,cg12995004, cg02361881,cg21578783,cg13335863,cg12442242,cg07277465,cg20674419, cg22541831,cg01174674,cg07269146,cg13998990,cg11516226,cg00879786, cg08043017,cg14975881,cg07063183,cg18021101,cg24616081,cg20728419, cg12027843,cg18644525,cg05720491,cg02252828,cg18876623,cg04128367, cg04143547,cg21050667,cg06442472,cg19038282,cg18199495,cg03826683, cg22073507,cg04563169,cg05269246,cg10040969,cg26797811,cg00575903, cg03437916,cg25760395,cg01220560,cg16356630,cg27248581,cg24102491, cg22489198,cg13086586,cg27429917,cg00701316,cg00388732,cg05567646, cg25037274,cg13132816,cg15586842,cg23101093,cg01919034,cg23351190, cg06659867,cg10347474,cg01808050,cg04550237,cg01996643,cg18264519, cg16430099,cg12375876,cg10765877,cg00861824,cg09931146,cg05250380, cg19513514,cg02404507,cg03258272,cg13078931,cg15471398,cg03887676, cg25997110,cg07926770,cg23769345,cg00834234,cg11085971,cg11812143, cg02975091,cg01388301,cg10478863,cg17970482,cg00666211,cg18562923, cg02288552,cg16243197,cg09664593,cg22814944,cg20674547,cg06142142, cg13328067,cg13131111,cg21948564,cg00702369,cg03221984,cg18496965, cg03986418,cg07793537,cg05773164,cg20030852,cg06034238,cg05105212, cg03311682,cg13758932,cg11227941,cg16851359,cg21803141,cg22170222, cg10693065,cg01135961,cg02129877,cg27642883,cg23647968,cg06151505, cg03778258,cg18617527,cg00605545,cg17073323,cg27431237,cg00427150, cg16648603,cg26040075,cg18917270,cg00261621,cg12344862,cg19961277, cg09758248,cg22085718,cg08564657,cg06808227,cg17063731,cg13642800, cg12283362,cg09736526,cg06557127,cg22943875,cg00363815,cg19461907, cg15213026,cg26243679,cg13357708,cg18945671,cg21590238,cg21825757, cg08748794,cg02711665,cg14742851,cg09581413,cg03845683,cg06211385, cg04583163,cg10172319,cg17249729,cg07295197,cg04551025,cg19646204, cg24437314,cg07132926,cg11556122,cg16912134,cg08403296,cg24695281, cg08622563,cg00110695,cg07792446,cg05389922,cg03039025,cg10426482, cg00477817,cg04626915,cg10362294,cg14080881,cg13020386,cg24591118, cg15277311,cg13934722,cg14431001,cg01101177,cg00800038,cg23237516, cg06754191,cg23816019,cg02630829,cg00657581,cg24220766,cg23299156, cg13461762,cg03615476,cg03853124,cg04963553,cg00535466,cg20863321, cg14847095,cg21179412,cg03595262,cg12052754,cg18884536,cg02509204, cg08857436,cg19777318,cg26427417,cg03216596,cg08109808,cg15967169, cg13930665,cg27094305,cg14550212,cg11587748,cg21592690,cg26183021, cg00682203,cg09822812,cg05043657,cg13341441,cg05717478,cg20171451, cg04628206,cg16613176,cg04649441,cg10370687,cg05628357,cg23295491, cg00850868,cg03395044,cg19932993,cg01774477,cg08012156,cg10586082, cg15379825,cg03415649,cg17286985,cg18193230,cg21944219,cg00470768, cg14290291,cg16135164,cg05168280,cg22035897,cg13637552,cg03393472, cg17590234,cg17809780,cg13976251,cg27562595,cg17375177,cg15378274, cg18724709,cg02380561,cg24306422,cg17462468,cg05340577,cg04805512,

Intersections	No. of overlapping DMPs	List of overlapping DMPs
		cg13978991,cg18561871,cg17861477,cg07351592,cg19987349,cg25215594,<br>cg07711067,cg06393998,cg15473395,cg16793607,cg10148231,cg04938335,<br>cg04826176,cg23020752,cg01289561,cg18589543,cg17875260,cg20125407,<br>cg09364127,cg25971128,cg01521225,cg12740087,cg02377024,cg07645858,<br>cg13134082,cg24651708,cg00718418,cg04916980,cg01957629,cg00246547,<br>cg13174660,cg20932762,cg00072407,cg10317162,cg05549970,cg26982323,<br>cg16929959,cg22635075,cg20668812,cg10995473,cg09486594,cg24727188,<br>cg17665552,cg15601859,cg01507833,cg08009313,cg10210928,cg12622520,<br>cg26776722,cg08537599,cg05863841,cg23714454,cg22748470,cg03516636,<br>cg26131927,cg19191502,cg01858458,cg03930577,cg13934990,cg14069835,<br>cg10593816,cg25203036,cg15990679,cg18532208,cg20740203,cg22238304,<br>cg18885872,cg24171807,cg26994859,cg05389034,cg18641433,cg05431656,<br>cg11736740,cg26650701,cg07470284,cg14248680,cg01734338,cg15715969,<br>cg17131053,cg11610831,cg26281971,cg11640384,cg02744713,cg25294651,<br>cg04040935,cg19563410,cg02993013,cg01782154,cg03865947,cg10612060,<br>cg22636631,cg10568796,cg03694875,cg00872232,cg13298827,cg24063594,<br>cg04254389,cg09762054,cg26484361,cg04109704,cg05362860,cg02104456,<br>cg16300033,cg17505339,cg12447937,cg03291430,cg19493162,cg09231555,<br>cg19487745,cg21909286,cg22629515,cg24777071,cg11463522,cg24805307,<br>cg26301777,cg11791725,cg09497391,cg22604496,cg00781795,cg23434122,<br>cg03734677,cg25828607,cg23101469,cg18192414,cg26747301,cg20018396,<br>cg09556178,cg07612889,cg07061975,cg03788567,cg27107611,cg03261347,<br>cg26103331,cg21618694,cg08550882,cg11361049,cg04349266,cg19210342,<br>cg19532617,cg10716356,cg23597907,cg16790676,cg23878917,cg18803747,<br>cg24636611,cg20526432,cg20715291,cg02376664,cg17313042,cg16547125,<br>cg23748584,cg08095345,cg04025244,cg08254315,cg11152012,cg09153458,<br>cg22813165,cg23828422,cg04031454,cg27383744,cg05607320,cg08160526,<br>cg03805929,cg15287541,cg13629903,cg22462000,cg03696592,cg00954931,<br>cg22167093,cg22450197,cg19406863,cg15115413,cg16736799,cg02952548,<br>cg11786283,cg00914084,cg03143333,cg12524776,cg03198186,cg14377342,<br>cg24607603,cg01292924,cg07997387,cg02848097,cg10747758,cg15912014,<br>cg23365723,cg09501333,cg24291485,cg05950923,cg03833512,cg27539472,<br>cg26886965,cg13613011,cg09177194,cg03355286,cg12185938,cg13514165,<br>cg18516228,cg21827626,cg02190786,cg06766579,cg12204171,cg06424550,<br>cg04815812,cg16999495,cg07080785,cg05636912,cg19957411,cg26612736,<br>cg15632673,cg02915805,cg07027119,cg01374440,cg13064046,cg17559292,<br>cg15067386,cg03344608,cg27268640,cg25597177,cg05180512,cg11177858,<br>cg15493018,cg02294570,cg22942210,cg00086429,cg23065757,cg13386084,<br>cg14435659,cg17364250,cg09545764,cg04605148,cg02586949,cg26532930,<br>cg20415697,cg00800809,cg02292720,cg09466312,cg20349574,cg23055064,<br>cg05560494,cg02767673,cg01541565,cg22036487,cg00891176,cg27210269,<br>cg01202731,cg14465801,cg03584693,cg02583183,cg19281864,cg07468862,<br>cg20355676,cg25265930,cg25712758,cg08970795,cg22712955,cg08270888,<br>cg23158189,cg06613547,cg07016571,cg00448889,cg00603673,cg13823166,

Intersections	No. of overlapping DMPs	List of overlapping DMPs
		cg01182973,cg02998155,cg14835484,cg06574881,cg26394282,cg05570682,cg21512370,cg17714154,cg05331252,cg00847301,cg18450293,cg22676447,cg03902905,cg04983519,cg07473909,cg00989238,cg10141352,cg16826218,cg02362309,cg00408605,cg17929152,cg24337310,cg04204557,cg05269713,cg08223835,cg13571802,cg10555000,cg15587018,cg20449764,cg13910907,cg13838832,cg00221096,cg23175907,cg21756765,cg06215556,cg15535114,cg27553564,cg11806813,cg02572033,cg12525514,cg16713069,cg04792167,cg19950767,cg15246460,cg13316257,cg24696735,cg18867781,cg06338697,cg05239771,cg07421068,cg14516566,cg22230559,cg11748170,cg06624244,cg00599328,cg01341130,cg21220870,cg00513781,cg08874470,cg09050048,cg05152653,cg08447044,cg26154534,cg26279614,cg26915384,cg05834354,cg25737169,cg02317375,cg05286791,cg01333532,cg20930489,cg19565241,cg13394785,cg26113972,cg18474563,cg24212248,cg22523074,cg17420774,cg21413754,cg26720073,cg22466850,cg18908342,cg26227943,cg00975587,cg09884851,cg21562638,cg24700494,cg09747054,cg11641080,cg06223797,cg07801516,cg02247211,cg12635848,cg04114269,cg06585606,cg09979356,cg04824666,cg09461494,cg17820495,cg10188698,cg05565807,cg03808043,cg19614627,cg23825344,cg23528297,cg10202675,cg01104200,cg05977276,cg26892308,cg03039294,cg14202791,cg02747612,cg17431301,cg01696060,cg11325576,cg02255892,cg01092531,cg15434398,cg08547427,cg09243824,cg06599575,cg22654949,cg10173001,cg25141418,cg04097394,cg20852653,cg26502817,cg18079653,cg13639582,cg10635719,cg02656873,cg04917409,cg12158165,cg02553302,cg22284302,cg02981041,cg22351824,cg23389807,cg03729042,cg17128082,cg08703289,cg14104700,cg24246245,cg26414564,cg23819027,cg16358215,cg20105087,cg18935740,cg10369313,cg01523027,cg03752430,cg22909296,cg05889294,cg07773272,cg03268737,cg01518398,cg11823382,cg20073882,cg21700831,cg26629556,cg27480957,cg23712897,cg16919579,cg09124018,cg10797856,cg24096828,cg11979618,cg15637781,cg23486661,cg04462547,cg01825203,cg18360585,cg08237400,cg22807576,cg16996457,cg16359654,cg11327565,cg04910692,cg17868778,cg14349131,cg18304242,cg17378655,cg24328054,cg07029084,cg09816107,cg03731251,cg07180182,cg02868123,cg07709195,cg11968166,cg24395241,cg13549760,cg26182313,cg04990609,cg15118519,cg22459078,cg08857545,cg03057072,cg03926968,cg19969431,cg01607187,cg20138055,cg11709211,cg12502155,cg26566411,cg00779056,cg20834966,cg08885100,cg03929077,cg04849201,cg00531054,cg01335566,cg18011078,cg13484614,cg08056835,cg01255646,cg18175611,cg10930676,cg10703101,cg10239978,cg02044172,cg03195015,cg04226766,cg12492289,cg21287017,cg19123462,cg20027684,cg15069785,cg11265952,cg00022606,cg22990888,cg17916447,cg21830726,cg06419562,cg00081852,cg13653680,cg12130250,cg11677105,cg10819981,cg09563451,cg02130266,cg02903279,cg11239335,cg14027704,cg24517611,cg26675905,cg24901609,cg11289591,cg06577028,cg23968650,cg23901843,cg01761277,cg10220850,cg27513225,cg24755297,cg12178432,cg01320648,cg13487422,cg17971210,cg09516898,cg05841219,cg15843977,cg00043080,cg23058911,

Intersections	No. of overlapping DMPs	List of overlapping DMPs
		cg10930667,cg17145983,cg27560687,cg12946080,cg04076763,cg13817353,<br>cg21632117,cg08880904,cg03353804,cg04285493,cg12440728,cg12610079,<br>cg00776427,cg02386644,cg02262873,cg02237704,cg22922010,cg24467825,<br>cg05153791,cg10174975,cg03169856,cg21173221,cg20788133,cg22875855,<br>cg22048274,cg15758403,cg21046954,cg16149628,cg27662204,cg10544839,<br>cg27430465,cg09267674,cg05925927,cg27208012,cg16563180,cg16054502,<br>cg16494843,cg25209294,cg10702394,cg06673956,cg15120497,cg01892997,<br>cg25598062,cg25619312,cg08455021,cg24556854,cg20662018,cg00263326,<br>cg23873683,cg07380384,cg22721170,cg20299105,cg24805126,cg13781498,<br>cg15768620,cg10727416,cg14052688,cg02243606,cg01834029,cg18918155,<br>cg26511337,cg00378007,cg14400179,cg08858643,cg07553580,cg03646967,<br>cg23331966,cg11589509,cg03789934,cg09499379,cg26979044,cg22964775,<br>cg06846752,cg27630274,cg19493789,cg01960043,cg07437919,cg00217442,<br>cg01880743,cg14276973,cg06526042,cg14180758,cg00693222,cg24023023,<br>cg08929467,cg22179018,cg04823492,cg07602984,cg08845025,cg25540004,<br>cg03946106,cg01165203,cg01618287,cg09494546,cg02637052,cg13562011,<br>cg07959403,cg26153497,cg07442204,cg13453110,cg21196492,cg22534919,<br>cg06238779,cg25798479,cg27312049,cg27131197,cg12132429,cg02888513,<br>cg18892132,cg04830471,cg01369276,cg01526919,cg11793413,cg06812099,<br>cg19517014,cg15720500,cg09741995,cg12520727,cg15988785,cg19534906,<br>cg10431365,cg08098111,cg06576765,cg16807420,cg17975976,cg08849542,<br>cg26845430,cg02845997,cg07456314,cg14168996,cg07461370,cg14971418,<br>cg03747003,cg01050740,cg05791482,cg07054526,cg23367286,cg13690703,<br>cg13224855,cg19052829,cg20587332,cg18529251,cg03825238,cg05644735,<br>cg11890795,cg25922355,cg03672499,cg10113618,cg22723802,cg00528779,<br>cg04551318,cg25958062,cg02813542,cg24845763,cg00481415,cg13464157,<br>cg18975690,cg07798407,cg25851488,cg18280933,cg07834934,cg26148377,<br>cg07913806,cg04940526,cg26585326,cg20252517,cg07790939,cg03132202,<br>cg07232688,cg14222630,cg23003872,cg23081488,cg24690069,cg18467041,<br>cg11644123,cg20891622,cg08489682,cg25666995,cg04935792,cg19515510,<br>cg19744051,cg12510108,cg03201367,cg11852073,cg24451888,cg01779076,<br>cg11165316,cg05689028,cg13046801,cg23684316,cg02554626,cg14582970,<br>cg02561720,cg04307553,cg03502656,cg07283616,cg07444152,cg05796504,<br>cg19265948,cg02528862,cg14156768,cg24421839,cg00974107,cg01634102,<br>cg14567260,cg06341189,cg09073441,cg26020008,cg24741793,cg13278241,<br>cg16946007,cg24763467,cg10615065,cg05045104,cg26664737,cg14231674,<br>cg04090794,cg11401986,cg04892998,cg09067747,cg25564733,cg08097057,<br>cg09096967,cg11409139,cg01905618,cg00215922,cg13210820,cg27633762,<br>cg00236077,cg01998262,cg10496659,cg03199084,cg22271515,cg18085869,<br>cg03039797,cg09387867,cg19644336,cg17653190,cg22574586,cg01881525,<br>cg09595245,cg13431883,cg00110039,cg20994227,cg16402142,cg01403607,<br>cg02290197,cg07028249,cg09399225,cg06692606,cg10197405,cg03036510,<br>cg09551645,cg24091975,cg26447146,cg02089795,cg27440751,cg10620911,<br>cg19453353,cg02603868,cg13279713,cg17761225,cg04960798,cg24334230,

Intersections	No. of overlapping DMPs	List of overlapping DMPs
		cg19554235,cg18428201,cg03188356,cg12014368,cg01816714,cg20609972, cg16381523,cg00699996,cg05435581,cg15085431,cg23210127,cg03408497, cg21696677,cg04671614,cg13511067,cg04947561,cg01864295,cg14711592, cg20047924,cg09344219,cg17875584,cg08094135,cg19689922,cg02507657, cg10897631,cg13660998,cg16884295,cg18002519,cg17117697,cg22692843, cg27078727,cg24375690,cg11466485,cg06970744,cg15288420,cg14035828, cg06560422,cg12483682,cg17435182,cg11320544,cg08342092,cg06846186, cg23287661,cg00458652,cg10455503,cg14043752,cg26453295,cg20959851, cg03539808,cg09608716,cg12757890,cg13471254,cg25676890,cg06113303, cg03316030,cg17445557,cg07188198,cg10140613,cg08254733,cg24304733, cg09952674,cg11973716,cg06025981,cg22993400,cg00473045,cg09150309, cg23477940,cg10214968,cg03113740,cg00380846,cg18150687,cg26385011, cg08928569,cg26986620,cg02094071,cg19227511,cg00863716,cg13649036, cg07257190,cg26318321,cg01824349,cg20627903,cg02610070,cg12699127, cg02241111,cg25428494,cg05371599,cg21160906,cg17457379,cg27324781, cg05153057,cg21096345,cg19528371,cg03537567,cg06101841,cg15611936, cg03012728,cg19410143,cg25758137,cg00534626,cg24509398,cg19840093, cg22681114,cg12556608,cg26670636,cg14427563,cg13282547,cg27162642, cg05904806,cg03115746,cg10877458,cg07282454,cg13362404,cg06124110, cg02496065,cg17795265,cg05042213,cg27165687,cg27234747,cg12459603, cg25030549,cg26922625,cg24700515,cg03530260,cg14061886,cg14038618, cg08492178,cg00808352,cg09908014,cg23219808,cg20836546,cg03373960, cg19350479,cg12048617,cg25644323,cg17001868,cg02923669,cg14421779, cg11564268,cg07780534,cg00066512,cg07222908,cg20311065,cg06957677, cg03767562,cg26269630,cg02360478,cg10747603,cg13551841,cg01106989, cg03045014,cg15480962,cg02432009,cg20675440,cg12903648,cg14404146, cg04546557,cg15238664,cg14216331,cg00002473,cg02773399,cg03410632, cg09660211,cg07398558,cg14220634,cg24139720,cg01843804,cg09991426, cg06945747,cg22924269,cg18344056,cg10041379,cg06182770,cg16077168, cg21213571,cg27170159,cg23171636,cg19239930,cg22482048,cg07762785, cg10326403,cg06323624,cg19647197,cg16786721,cg00292073,cg26933865, cg10108539,cg12800145,cg00015373,cg16578802,cg20559736,cg04194066, cg18659028,cg15894467,cg26663742,cg01185138,cg09474476,cg02238387, cg08045489,cg17178777,cg06698828,cg14641774,cg17862468,cg16558333, cg02232667,cg09739850,cg13957124,cg04851992,cg22037121,cg11721273, cg02424995,cg12224855,cg24630782,cg19269323,cg13832059,cg23099740, cg06737484,cg07598324,cg13597865,cg23989712,cg20197694,cg04224084, cg21255826,cg21771473,cg16518825,cg03697411,cg25187622,cg05105016, cg07756562,cg03551378,cg04943440,cg15076820,cg04900179,cg01456626, cg07034700,cg16134501,cg11105927,cg14199837,cg10047762,cg00934997, cg20803076,cg14263351,cg07378778,cg04130408,cg02918786,cg04831589, cg19350469,cg11041239,cg21616720,cg12514654,cg17145652,cg15543199, cg19069283,cg25211348,cg14831653,cg14485581,cg11361658,cg22573590, cg03792839,cg09096476,cg01166159,cg25435714,cg05638883,cg26731834,

Intersections	No. of overlapping DMPs	List of overlapping DMPs
		cg06250423,cg01531551,cg08159831,cg04084052,cg03612700,cg04757410, cg27022602,cg00935351,cg18401511,cg20022118,cg15155568,cg01006587, cg26701810,cg08356248,cg14827285,cg08137638,cg22355895,cg05323272, cg04181696,cg02964359,cg19566878,cg23960922,cg03523434,cg14754243, cg24279701,cg27372181,cg23877512,cg18021100,cg09158651,cg27421105, cg03014011,cg18553202,cg09739912,cg25916771,cg27095983,cg16427530, cg04677937,cg01766505,cg11136406,cg01925662,cg14134015,cg13675624, cg05329180,cg03560936,cg22236073,cg10677688,cg00644922,cg20667822, cg08983259,cg03640443,cg11489821,cg13221681,cg20389750,cg10515950, cg19665882,cg02397533,cg01783051,cg13024585,cg27392815,cg18135704, cg26638920,cg22825550,cg22731215,cg03770187,cg03849728,cg04098176, cg16792978,cg06868699,cg21185320,cg03654080,cg14093289,cg09259004, cg21835298,cg14185323,cg15648790,cg23406815,cg06970454,cg23615248, cg08831522,cg20416574,cg12707723,cg25047058,cg02290855,cg12576523, cg13637899,cg07240043,cg05310646,cg14321777,cg07162124,cg13877944, cg22871659,cg10631684,cg05933901,cg08459182,cg06084379,cg08751025, cg09497124,cg10045446,cg25039902,cg26902504,cg14844138,cg13036878, cg10295126,cg08371086,cg15132055,cg00702837,cg01323706,cg25552017, cg23402397,cg01579172,cg06745965,cg14394011,cg04434491,cg15623444, cg02462962,cg15278957,cg13154024,cg26001590,cg00418479,cg20836993, cg01348594,cg14470762,cg12470721,cg11443973,cg24300962,cg01079307, cg18296448,cg09592546,cg10146330,cg24542200,cg24792360,cg01772945, cg24182328,cg20895092,cg23423607,cg05073382,cg10059324,cg11226289, cg08329561,cg12478809,cg06988547,cg03710889,cg25283432,cg01270736, cg11438448,cg02298570,cg08947084,cg06670380,cg15027630,cg22683036, cg05798318,cg05640721,cg09168728,cg13749477,cg11859345,cg24082680, cg08516792,cg20237264,cg22062537,cg21300361,cg07518330,cg06366791, cg26408612,cg17951488,cg07628769,cg26701510,cg15726387,cg13606720, cg26824826,cg14570121,cg11111835,cg24505687,cg01810763,cg16678686, cg03601368,cg00053536,cg17129519,cg17484267,cg07730946,cg04102427, cg12810701,cg13770865,cg00837103,cg02169692,cg15501415,cg09536337, cg10211626,cg20468462,cg04730459,cg10202544,cg23014672,cg24351819, cg25513090,cg27547695,cg23806014,cg08428949,cg18699242,cg03085932, cg20255560,cg16452272,cg26075252,cg04072156,cg25151803,cg23087661, cg11379605,cg21515494,cg18920490,cg03567106,cg10945028,cg08136070, cg00445013,cg06438114,cg00590330,cg25964019,cg18183019,cg24337318, cg18822580,cg18758405,cg10071848,cg09425611,cg20465830,cg00975912, cg07020453,cg05444548,cg24803614,cg19075506,cg01864361
- ASD+16p11.2 del	39	cg09592546,cg10146330,cg24542200,cg24792360,cg01772945,cg24182328, cg20895092,cg23423607,cg05073382,cg10059324,cg11226289,cg08329561, cg12478809,cg06988547,cg03710889,cg25283432,cg01270736,cg11438448, cg02298570,cg08947084,cg06670380,cg15027630,cg22683036,cg05798318, cg05640721,cg09168728,cg13749477,cg11859345,cg24082680,cg08516792,

Intersections	No. of overlapping DMPs	List of overlapping DMPs
		cg20237264,cg22062537,cg21300361,cg07518330,cg06366791,cg26408612, cg17951488,cg07628769,cg26701510
- ASD+CHD8	101	cg15726387,cg13606720,cg26824826,cg14570121,cg11111835,cg24505687, cg01810763,cg16678686,cg03601368,cg00053536,cg17129519,cg17484267, cg07730946,cg04102427,cg12810701,cg13770865,cg00837103,cg02169692, cg15501415,cg09536337,cg10211626,cg20468462,cg04730459,cg10202544, cg23014672,cg24351819,cg25513090,cg27547695,cg23806014,cg08428949, cg18699242,cg03085932,cg20255560,cg16452272,cg26075252,cg04072156, cg25151803,cg23087661,cg11379605,cg21515494,cg18920490,cg03567106, cg10945028,cg08136070,cg00445013,cg06438114,cg00590330,cg25964019, cg18183019,cg24337318,cg18822580,cg18758405,cg10071848,cg09425611, cg20465830,cg00975912,cg07020453,cg05444548,cg24803614,cg19075506, cg01864361,cg03514928,cg09421589,cg03798915,cg20723844,cg07236884, cg08889636,cg08005007,cg09041268,cg12190125,cg26171505,cg19717943, cg26795352,cg10805511,cg22706070,cg22993878,cg16773563,cg15564547, cg26953096,cg00984715,cg18823499,cg17837127,cg27072545,cg06385000, cg04154142,cg02401554,cg20033808,cg06421197,cg03331853,cg16608772, cg07131553,cg18221988,cg04161784,cg17291922,cg16436164,cg12315011, cg14206365,cg03870862,cg17122301,cg14115346,cg19201533

Supplementary Table S7. Utilized gene expression microarrays/RNA-sequencing for the differential gene expression analysis of the target genes of ASD with 16p11.2del.

Methylome data				Transcriptome data				
ProbeID	Elements	Delta	P_{FDR}	GSE	Gene ID	Gene	log_2FC	q-value
cg22062537	L1MB3	-0.079	1.52E-04	GSE25507	1553515_at	*MYEOV2*	0.107	0.0496
cg13749477	L1MB3	-0.038	3.58E-04	GSE25507	1553515_at	*MYEOV2*	0.107	0.0496
cg07628769	L1MB3	-0.101	2.76E-03	GSE25507	1553515_at	*MYEOV2*	0.107	0.0496
cg01772945	AluSq	-0.048	2.81E-04	GSE18123	205739_x_at	*ZNF107*	0.382	0.0017
cg09168728	HAL1	0.042	3.94E-03	GSE18123	202651_at	*LPGAT1*	0.160	0.0038
cg21300361	AluY	0.024	1.39E-02	GSE18123	1553640_at	*XKR6*	-0.949	0.0206
cg21300361	AluY	0.024	1.39E-02	GSE25507	1553640_at	*XKR6*	0.134	0.0112
cg01270736	AluJb	-0.037	1.67E-02	GSE25507	207289_at	*MMP25*	0.193	<0.0001
cg26620682	L1PA2	-0.058	2.06E-02	GSE28521_FC	ILMN_1765641	*SEMA3A*	-0.200	0.0343
cg26620682	L1PA2	-0.058	2.06E-02	GSE59288	10371	*SEMA3A*	-0.590	0.0139
cg05073382	L1MA7	0.153	2.90E-02	GSE89594	A_23_P258912	*MYOM2*	-1.611	0.0114
cg11438448	AluSx4	-0.014	3.64E-02	GSE18123	233429_at	*SPEF2*	-0.285	0.0484
cg10059324	L1MC4a	0.050	3.68E-02	GSE59288	8863	*PER3*	0.296	0.0269
cg11859345	AluSp	-0.031	4.74E-02	GSE18123	228766_at	*CD36*	0.171	0.0249
cg11859345	AluSp	-0.031	4.74E-02	GSE18123	206488_s_at	*CD36*	0.273	0.0076
cg11859345	AluSp	-0.031	4.74E-02	GSE25507	209554_at	*CD36*	0.099	0.0131
cg25283432	L1MA8	0.004	4.82E-02	GSE25507	232421_at	*SCARB1*	0.116	0.0189

Supplementary Table S8. Utilized gene expression microarrays/RNA-sequencing for the differential gene expression analysis of the target genes of ASD with *CHD8* variant.

Methylome data				Transcriptome data				
ProbeID	Elements	Delta	P_{FDR}	GSE	Gene ID	Gene	log_2FC	q-value
cg10071848	AluJb	-0.033	4.39E-02	GSE59288	5826	ABCD4	0.39	0.0078
cg07730946	AluJb	-0.027	1.28E-02	GSE42133	ILMN_1684585	ACSL1	-0.24	0.0344
cg12934569	AluSx	-0.043	3.19E-02	GSE42133	ILMN_1684585	ACSL1	-0.24	0.0344
cg11111835	L1MA3	-0.020	3.59E-02	GSE18123	1553603_s_at	ATL2	0.28	0.0248
cg11111835	L1MA3	-0.020	3.59E-02	GSE18123	237968_at	ATL2	-0.25	0.0492
cg04154142	AluSx1	-0.063	1.10E-02	GSE42133	ILMN_1672596	BCAR1	-0.09	0.0127
cg04154142	AluSx1	-0.063	1.10E-02	GSE18123	223116_at	BCAR1	-0.26	0.0226
cg14115346	L1MB3	-0.038	2.01E-02	GSE18123	205750_at	BPHL	0.28	0.0097
cg10211626	AluSx1	-0.059	2.82E-03	GSE59288	79640	C22orf46	0.56	0.0002
cg17129519	AluSg	0.097	3.60E-02	GSE64018	ENSG00000204564	C6orf136	-0.18	0.0430
cg17129519	AluSg	0.097	3.60E-02	GSE28521_TC	ILMN_1813236	C6orf136	-0.30	<0.0001
cg06421197	AluJo	-0.034	2.66E-02	GSE18123	206011_at	CASP1	0.14	0.0122
cg06421197	AluJo	-0.034	2.66E-02	GSE42133	ILMN_2326509	CASP1	-0.16	0.0258
cg06421197	AluJo	-0.034	2.66E-02	GSE42133	ILMN_2326512	CASP1	-0.17	0.0213
cg06421197	AluJo	-0.034	2.66E-02	GSE42133	ILMN_1727762	CASP1	-0.25	<0.0001
cg06421197	AluJo	-0.034	2.66E-02	GSE59288	834	CASP1	0.61	0.0096
cg13606720	L1ME3F	-0.044	1.25E-02	GSE42133	ILMN_3248676	CBWD3	-0.11	0.0180
cg11379605	AluJb	-0.033	3.59E-02	GSE59288	965	CD58	0.47	0.0159
cg11379605	AluJb	-0.033	3.59E-02	GSE18123	222061_at	CD58	0.36	0.0256
cg11379605	AluJb	-0.033	3.59E-02	GSE64018	ENSG00000116815	CD58	0.50	0.0328
cg09425611	L1MB7	-0.028	3.60E-02	GSE18123	209616_s_at	CES1	0.44	0.0025
cg00053536	AluSx	-0.028	4.62E-02	GSE25507	206274_s_at	CROCC	0.12	0.0458
cg09604414	AluSx	-0.067	3.36E-02	GSE25507	229079_at	EHMT2	0.09	0.0471
cg22706070	L1MC5	-0.008	4.96E-02	GSE25507	229079_at	EHMT2	0.09	0.0471
cg14570121	FLAM_A	-0.031	3.75E-02	GSE59288	2068	ERCC2	-0.26	0.0149

cg24803614	AluSc8	0.051	3.41E-02	GSE59288	2495	FTH1	0.42	0.0045
cg03085932	L1PA6	-0.043	4.59E-02	GSE25507	220249_at	HYAL4	0.12	0.0179
cg08005007	L1HS	-0.031	9.50E-03	GSE18123	235111_at	LSAMP	-0.23	0.0386
cg08005007	L1HS	-0.031	9.50E-03	GSE18123	229244_at	LSAMP	-0.25	0.0303
cg22993878	L1MB3	-0.021	7.98E-03	GSE25507	1553515_at	MYEOV2	0.11	0.0496
cg03514928	L1PA17	-0.022	4.70E-02	GSE59288	4897	NRCAM	-0.31	0.0104
cg20723844	AluY	-0.061	1.63E-02	GSE42133	ILMN_1789616	NUPL2	-0.09	0.0221
cg00984715	AluSq	-0.049	2.24E-02	GSE59288	5465	PPARA	0.51	0.0149
cg00984715	AluSq	-0.049	2.24E-02	GSE18123	1560981_a_at	PPARA	-0.24	0.0256
cg00984715	AluSq	-0.049	2.24E-02	GSE18123	1558631_at	PPARA	-0.46	0.0091
cg24351819	AluSp	-0.026	4.74E-02	GSE59288	55170	PRMT6	-0.33	0.0057
cg09521141	AluSg7	-0.045	2.63E-02	GSE28521_FC	ILMN_1765641	SEMA3A	-0.20	0.0343
cg09521141	AluSg7	-0.045	2.63E-02	GSE59288	10371	SEMA3A	-0.59	0.0139
cg26620682	L1PA2	-0.041	3.46E-02	GSE28521_FC	ILMN_1765641	SEMA3A	-0.20	0.0343
cg26620682	L1PA2	-0.041	3.46E-02	GSE59288	10371	SEMA3A	-0.59	0.0139
cg18221988	L1PA2	-0.043	4.99E-02	GSE18123	210804_x_at	SLC8A1	-0.30	0.0392
cg18221988	L1PA2	-0.043	4.99E-02	GSE25507	1565306_a_at	SLC8A1	0.18	0.0189
cg18221988	L1PA2	-0.043	4.99E-02	GSE59288	6546	SLC8A1	-0.41	0.0245
cg18221988	L1PA2	-0.043	4.99E-02	GSE18123	235518_at	SLC8A1	0.29	0.0057
cg18221988	L1PA2	-0.043	4.99E-02	GSE64018	ENSG00000183023	SLC8A1	-0.26	0.0430
cg07020453	L1MDa	-0.009	4.57E-02	GSE18123	217968_at	TSSC1	-0.21	0.0185
cg08428949	AluSz	-0.028	1.24E-02	GSE64018	ENSG00000128881	TTBK2	-0.15	0.0352
cg01810763	AluSx1	-0.053	4.42E-02	GSE64018	ENSG00000198431	TXNRD1	0.30	0.0443
cg11191744	AluY	-0.050	4.57E-02	GSE64018	ENSG00000198431	TXNRD1	0.30	0.0443
cg02169692	AluSx	-0.109	2.75E-02	GSE42133	ILMN_3240420	USP18	-0.31	0.0250
cg02169692	AluSx	-0.109	2.75E-02	GSE42133	ILMN_3240420	USP18	-0.31	0.0250
cg18699242	AluSx	-0.106	4.48E-02	GSE42133	ILMN_3240420	USP18	-0.31	0.0250
cg18699242	AluSx	-0.106	4.48E-02	GSE42133	ILMN_3240420	USP18	-0.31	0.0250
cg15726387	AluSz6	-0.044	9.50E-03	GSE18123	220079_s_at	USP48	0.10	0.0053
cg15726387	AluSz6	-0.044	9.50E-03	GSE18123	225925_s_at	USP48	0.15	<0.0001

cg06385000	AluSc	-0.041	2.16E-02	GSE18123	227434_at	WBSCR17	-0.46	0.0392
cg06385000	AluSc	-0.041	2.16E-02	GSE28521_FC	ILMN_1701557	WBSCR17	-0.19	0.0402
cg06385000	AluSc	-0.041	2.16E-02	GSE28521_TC	ILMN_1701557	WBSCR17	-0.23	0.0485
cg03870862	L1MB3	-0.063	3.18E-02	GSE59288	23144	ZC3H3	0.27	0.0193

Supplementary Table S9. Characteristics of the Infinium450K datasets in the present study including ASD and non-ASD. The numbers of patients for each group are given for each gender. The ages for each group are given as means ± standard deviation.

Groups	Gender		Age
	Male	Female	
GSE113967: Blood			
ASD	45	7	10.45 ± 3.31
Non-ASD	33	15	10.22 ± 3.95
ASD+*CHD8*	11	4	8.97 ± 4.11
ASD+16p11.2 del	6	1	6.78 ± 4.05
GSE131706: Subventricular zone of the lateral ventricles			
ASD	17	-	28.85 ± 21.17
Non-ASD	17	-	27.37 ± 19.56
GSE80017: Prefrontal cortex			
ASD	6	3	NA
Non-ASD	5	4	NA
